# Extensive Recombination Suppression and Genetic Degeneration of a Young ZW Sex Chromosome System in Halfbeak Fish

**DOI:** 10.1093/molbev/msaf151

**Published:** 2025-06-24

**Authors:** Teng-Fei Xing, Yu-Long Li, Hao Yang, Deborah Charlesworth, Jin-Xian Liu

**Affiliations:** Key Laboratory of Marine Ecology and Environmental Sciences, Institute of Oceanology, Chinese Academy of Sciences, Qingdao 266071, China; Laboratory for Marine Ecology and Environmental Science, Qingdao Marine Science and Technology Center, Qingdao 266237, China; Key Laboratory of Marine Ecology and Environmental Sciences, Institute of Oceanology, Chinese Academy of Sciences, Qingdao 266071, China; Laboratory for Marine Ecology and Environmental Science, Qingdao Marine Science and Technology Center, Qingdao 266237, China; Key Laboratory of Marine Ecology and Environmental Sciences, Institute of Oceanology, Chinese Academy of Sciences, Qingdao 266071, China; Laboratory for Marine Ecology and Environmental Science, Qingdao Marine Science and Technology Center, Qingdao 266237, China; University of Chinese Academy of Sciences, Beijing 100049, China; Institute of Ecology and Evolution, School of Biological Sciences, University of Edinburgh, Edinburgh EH9 3FL, UK; Key Laboratory of Marine Ecology and Environmental Sciences, Institute of Oceanology, Chinese Academy of Sciences, Qingdao 266071, China; Laboratory for Marine Ecology and Environmental Science, Qingdao Marine Science and Technology Center, Qingdao 266237, China

**Keywords:** sex chromosomes, halfbeaks, recombination suppression, evolution, genome

## Abstract

Sex chromosome systems have evolved independently across the tree of life, at different times in the past, and the evolutionary consequences of lacking recombination in sex-linked regions have been characterized in many old-established systems. However, empirical studies of young sex chromosomes are still scarce, especially in vertebrates. Integrating whole-genome sequencing data of two species of halfbeak fish, *Hyporhamphus sajori* and *Hyporhamphus intermedius*, we identified the sex-determining system in *H. sajori* as female heterogamety, involving a large fully sex-linked ZW region (∼26 Mb) on chromosome 5. The closest relative, *H. intermedius*, has a small sex-linked region on a different chromosome and shows male heterogamety, suggesting at least one turnover in this fish genus. The *H. sajori* sex-linked region includes two evolutionary strata, but the estimated Z-W divergence times are small, less than 3 million years for the older stratum, which is less than between the two species. Nevertheless, this evolutionarily young W-linked region is enriched with repetitive sequences, differs from the ancestral state by five inversions, and about one-third of its protein-coding genes have already become nonfunctional. Transcriptomic analysis suggests that some form of dosage compensation may already be evolving for some sex-linked genes.

## Introduction

Species with separate sexes are found across the entire eukaryote tree of life, and sex determination is frequently genetic and often associated with regions of rare or no recombination, termed sex-linked regions (SLRs) or sex chromosomes (reviewed by Bergero and Charlesworth 2009; Furman et al. 2020). It has long been understood that sex chromosomes (such as XY chromosomes of mammals or ZW chromosomes of birds and snakes) can evolve from a chromosome pair that differs only at a single, sex-determining locus. The loss of recombination between sex chromosomes, including subsequent sequence divergence and genetic degeneration of the Y (or W), is a case of convergent evolution across a variety of animals and plants (Bachtrog et al. 2014; Charlesworth 2015). While the effects of lacking recombination have been well characterized, the evolution of full sex-linkage is still not fully understood. Empirical data can help to test between different possible evolutionary causes of a lack of recombination and establish the mechanisms involved.

Several nonmutually exclusive hypotheses have been developed to explain why recombination does not occur between sex chromosomes. When suppressed recombination has evolved (rather than a sex-determining locus evolving within a previously nonrecombining region), this may be the consequence of establishment of a sexually antagonistic polymorphism (with one allele increasing male fitness and reducing female fitness) in a genome region linked to a sex-determining locus. Selection then favors the suppression of recombination between the two loci (Charlesworth and Charlesworth 1978 ; Charlesworth and Charlesworth 1980; Rice 1987). A recently proposed alternative possibility is that recombination is suppressed through neutral sequence divergence (variants in linkage disequilibrium [LD]) between the X and Y chromosomes, gradually expanding the fully SLR (Jeffries et al. 2021). Another proposal is that, although inversions arise in all genome regions, and may spread, causing local suppression of recombination in heterozygotes, these will often become fixed in populations and no longer suppress recombination because heterozygotes become rare; as inversions that arise in regions closely linked to a sex-determining polymorphism will remain heterozygous in the heterogametic sex, like the sex-determining alleles themselves, this might potentially lead to suppressed recombination evolving most often in such regions (Jay et al. 2024). This process might involve either inversions on Y (or W) chromosomes or other factors (reviewed in Jay et al. 2024) that similarly dominantly suppress recombination in heterozygotes including spread of DNA methylation (reviewed in Kent et al. 2017) and cis-acting recombination modifiers or trans-modifiers that recognize specific sequences and target them for double-strand breaks (Catcheside 1981). These ideas have also been combined with the idea that deleterious mutations in Y-linked genes might favor their down-regulation accompanied by up-regulation of expression of their functional X-linked alleles (Charlesworth 1978). It has been shown that, if the inversions causing suppressed recombination can revert to the standard arrangement, this can lead to suppressed recombination evolving most often in formerly partially Y-linked regions (Lenormand and Roze 2022), as reviewed by Muralidhar and Veller (2022). It is currently unclear whether this can act in biologically plausible situations (Charlesworth and Olito 2024), as it requires high recessivity of the deleterious mutations.

Once an initial nonrecombining region forms, it may expand under similar selective situations to those just outlined. This can generate so-called “evolutionary strata”; this term describes genomic regions that ceased recombining at distinct evolutionary times. The ages of nonrecombining regions can be estimated using phylogenies and sequence divergence estimates (Charlesworth 2021; Zhang et al. 2022; Hibbins et al. 2024).

Although inversions may sometimes be involved in the evolution of nonrecombining regions, as has been inferred in humans (Lemaitre et al. 2009) and *Carica papaya* (Gschwend et al. 2012), inversions are not essential for recombination suppression, as noted above. Also, other chromosomal rearrangements, including translocations between autosomes and sex chromosomes, can create linkage to SLRs. However, unless one sex is recombinationally inactive, most of the neo-sex chromosome (the former autosome) will usually continue to recombine with its homolog, even if the neo-sex chromosome is translocated to the nonrecombining part of the ancestral sex chromosome. Continued recombination across part of a neo-sex chromosome is observed in some stickleback species (Natri et al. 2013), though, in another fish, the spotted knifejaw (*Oplegnathus punctatus*), recombination is suppressed in a roughly 3 Mb neo-Y region near the centromere involved in a Robertsonian translocation that fused an autosome to the ancestral Y chromosome (Li et al. 2021).

Furthermore, even if recombination suppression has evolved, the inversions observed in several systems may not have caused it, as they may be consequences of a lack of recombination (which removes deleterious fitness effects of recombination between rearranged chromosomes), as in the case of some fungal mating-type regions (Sun et al. 2017).

The evolution of a nonrecombining region, by whatever process it originated, affects the evolution of the genes and sequences within it. Suppressed recombination reduces the efficacy of purifying selection, allowing an increase in deleterious substitutions in genes on Y and W chromosomes (reviewed by Charlesworth 2017). This will lead to accumulation of loss-of-function mutations and the deletion of entire Y/W-linked genes, explaining the high degeneration of heteromorphic old sex chromosomes like those in birds and mammals. However, in some amphibians, recombination patterns depend on phenotypic sex, rather than on genotypic sex. Recombination between X and Y chromosomes in occasional sex-reversed XY females prevents Y chromosome degeneration (Perrin 2009; Rodrigues et al. 2018), showing that degeneration requires complete absence of recombination, consistent with theoretical modeling results. Another, probably earlier, consequence of recombination suppression is that repetitive sequences, including transposable elements, are predicted to accumulate rapidly. This is not expected in recombining regions because repetitive sequences cause chromosome rearrangements that are disfavored by selection (Charlesworth et al. 1994). Accumulation of repetitive sequences may explain why Y chromosomes of some species are larger than their X counterparts, as in the plant *Silene latifolia* (Bergero and Charlesworth 2009).

Thus, although sex chromosome evolution has been studied over the past ∼50 years (see reviews by Charlesworth 1996; Charlesworth and Charlesworth 2000), alternative hypotheses for the evolution of complete sex-linkage still require empirical testing, and studies of the time-course of degeneration are still needed. Ancient systems like mammal XY or bird ZW chromosomes are unsuitable for such studies because their Y or W chromosomes stopped recombining and lost most of their genes tens or hundreds of millions of years ago, and degeneration is no longer evolving or is now a very slow process (Bellott et al. 2014). Studies of the early stage of sex chromosome evolution are needed to reveal the mechanistic details of how Y or W chromosomes became nonrecombining (for example, detecting inversions contributing to recombination suppression), and test whether genes in these regions are degenerating, and, if so, the time-course of degeneration (Charlesworth 2019, 2021). Young systems with incomplete degeneration, where X(Z)- and Y(W)-linked coding sequences can still be compared, are most informative for studying degeneration (Darolti et al. 2019) and can be used to test whether individual genes degenerate independently and whether dosage compensation for low expression evolves as they degenerate.

Here, we describe such details in two halfbeak fish species, in the Beloniformes genus *Hyporhamphus* (Hemiramphidae). Based on morphological characteristics, 18 different halfbeak species live in coastal waters of the western North Pacific (Collette and Su 1986). Two species studied here, *Hyporhamphus intermedius* and *Hyporhamphus sajori*, share most characters but differ from all western North Pacific species by having longer upper jaws and more vertebrae and predorsal scales, and are thought to be more closely related than either is to other congeneric species. To date, no secondary sexual characteristics or overt sexual dimorphisms in body size or coloration have been described for these or other Hemiramphidae species. In contrast, a related family Zenarchopteridae (often called viviparous halfbeaks) shows sexual dimorphism, as the anal fin of male adults is modified into a specialized male structure, termed an “andropodium” (Reznick et al. 2007). Sex determination and possible sex chromosome evolution have not previously been studied in Hemiramphidae species. In some other taxa, species diversity has been hypothesized to be associated with frequent turnovers of sex chromosome, which might be involved in speciation (El Taher et al. 2021). *Hyporhamphus* is the most species-rich genus in the Hemiramphidae and might therefore undergo turnovers. Comparisons between close relatives also provide good outgroups for inferring evolutionary changes in the sex chromosomes.

Our results revealed that *H. sajori* has a physically large SLR that is heterozygous in females (a female heterogametic or ZW system), while its close relative, *H. intermedius*, has male heterogamety (an XY system), indicating a turnover event. The *H. intermedius* physically very small SLR is surprising, as it is often thought that ZW systems generally evolve from XY systems, which might suggest that the XY system should have been evolving for longer and might evolved a larger nonrecombining region, as outlined above. We describe evidence confirming that the *H. sajori* system is evolutionarily young, and that recombination was recently suppressed, and that this has already led to some genetic degeneration. We evaluate the mechanism that caused this suppressed recombination, and ask whether degeneration was accompanied by dosage compensation of genes in the newly W-linked region.

## Results

### Phylogenetic Relationships of the Species Studied

**Fig. 1.**
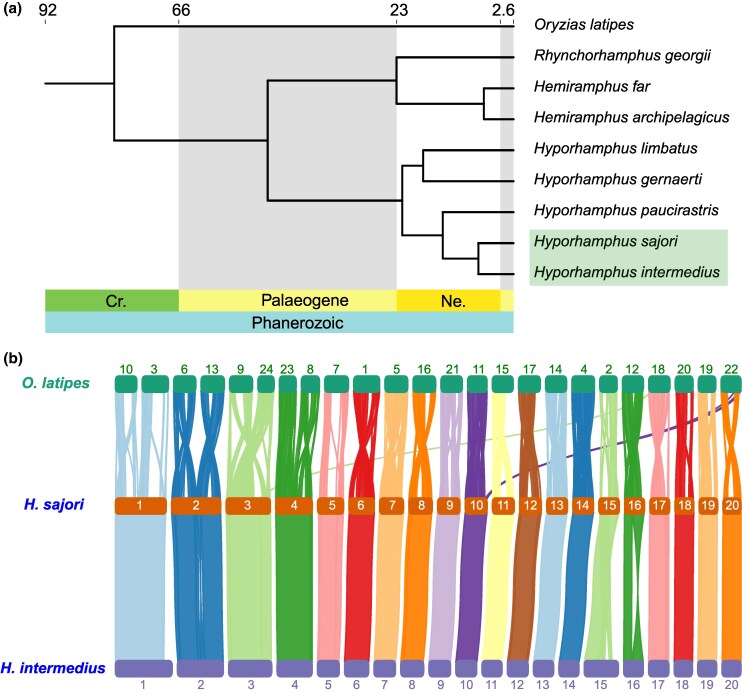
Phylogenetic relationships and genomic synteny for *H. sajori*, *H. intermedius*, and some related species. a) Phylogenetic tree estimated from single-copy genes of eight halfbeak species and *O. latipes*. b) Collinearity analysis of genes among *O. latipes*, *H. sajori*, and *H. intermedius* genomes.

We first constructed two independent phylogenetic trees to infer the relationships among *H. intermedius*, *H. sajori*, and other halfbeak species (Hemiramphidae). Using 13 mitochondrial protein-coding genes (PCGs) of seven species, a maximum likelihood tree (see Methods) suggested that, consistent with the morphological differences just mentioned, the two halfbeaks on which we focus here are close relatives, and the other *Hyporhamphus* species are outgroups (supplementary fig. S1, Supplementary Material online). We also collected tissue samples and generated whole-genome resequencing data with mean coverage of 13 × for each of six other halfbeak species (supplementary table S1, Supplementary Material online). The whole-genome phylogenetic tree constructed from single-copy genes (Fig. 1a) in all these species (see Methods) also suggested the closest relationship between *H. intermedius* and *H. sajori* (mean *K*s: 0.036) and shows that the medaka, *Oryzias latipes*, is a moderately close outgroup to the halfbeaks (though the mean *K*s between *H. sajori* and *O. latipes* are high, and the estimate using Nei and Gojobori 1986 correction for saturation is 0.776; supplementary fig. S2, Supplementary Material online).

### Karyotypes and Genome Assemblies of the Two Focal Species

The karyotype formula of *H. intermedius* is 2m + 38t, where m indicates metacentric and t telocentric; only one chromosome (one of the smallest chromosomes) is metacentric; of the telocentric chromosome group, t1–t4 are large, and the smaller chromosomes, t5–t19, are indistinguishable (Hong and Zhou 1984).

To assemble chromosome-level genomes of *H. intermedius* and *H. sajori*, we collected single female *H. intermedius* from Weishan Lake of Shandong Province in January 2021 and a male *H. sajori* individual from the nearshore waters of Qingdao, Shandong Province, China (see Materials and Methods). Using 29.04 Gb of PacBio HiFi reads (supplementary tables S2 and S3, Supplementary Material online), we assembled the *H. intermedius* female’s genome into 923 contigs, with a total length of 883.59 Mb and contig N50 of 3.98 Mb (supplementary table S4, Supplementary Material online). Combined with 76.46 Gb of Hi-C data, these contigs were anchored to 20 chromosomes, with a length totaling 883 Mb. A total of 24.52 Gb of HiFi reads and 114.11 Gb of Hi-C data were generated for the *H. sajori* male (supplementary tables S2 and S3, Supplementary Material online). The final assembly was 817 Mb, with a contig N50 of 14.94 Mb, again assembled and anchored to 20 chromosomes, with lengths ranging from 28.03 Mb to 79.08 Mb (supplementary table S4, Supplementary Material online). Totals of 12.48 Gb and 11.07 Gb of transcriptome sequences (extracted from multiple tissues including muscle, liver, heart, gill, eye, and brain) were generated for the same female *H. intermedius* and male *H. sajori* individuals (supplementary table S3, Supplementary Material online), and used for gene annotation by combined homologous protein alignment, ab initio prediction, and RNA-seq data prediction (see Methods), yielding 26,589 and 24,319 protein-coding sequences for the two species, respectively. The quality of the genome assemblies was assessed with the benchmarking universal single copy orthologs (BUSCO) pipeline, using the coverage of Actinopterygii core genes; the two assemblies retrieved 98.1% and 99.3% of the conserved single-copy orthologous genes, respectively (supplementary fig. S3, Supplementary Material online).

HiC analysis of *H. sajori* and *H. intermedius* also anchored both assemblies to 20 chromosomes (supplementary table S5, Supplementary Material online). We numbered the *H. sajori* chromosomes according to their assembly lengths, from the longest (HsaChr1) to the shortest, and those of *H. intermedius* according to their homologies with *H. sajori* chromosomes (Fig. 1b). The four largest chromosomes in both species (HsaChr1-4 and HinChr1-4) are homologs, based on their gene contents, and correspond to the four large telocentric t1–t4 chromosomes in the karyotype (supplementary fig. S4 and supplementary tables S5 and S6, Supplementary Material online). The chromosome numbered 16 is the only metacentric chromosome in our assemblies of both species, HsaChr16 and HinChr16. The remaining 15 chromosomes are acrocentric. All these results support the accuracy of our genomic assembly. Given the close relationship between *H. sajori* and *H. intermedius*, their ancestor probably shared the same karyotype.

### SLRs in the Two Halfbeak Species

**Fig. 2.**
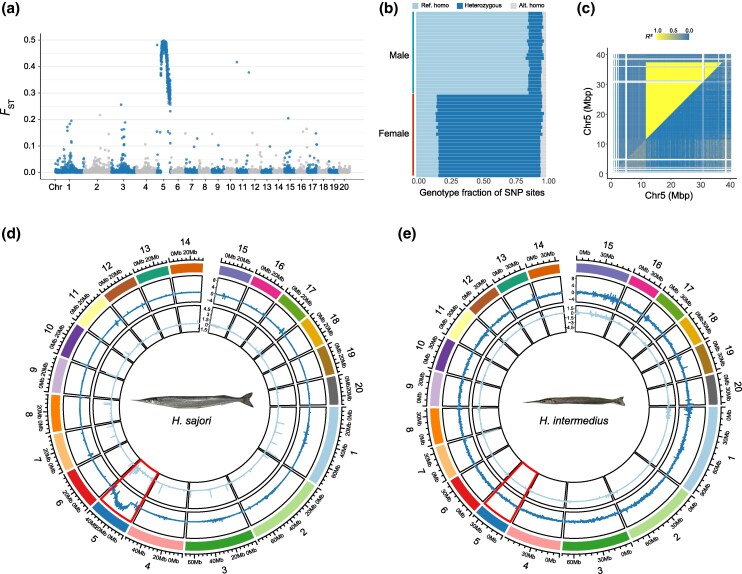
Evolution of sex chromosome in *H. sajori*. a) Mean *F*_ST_ values between females and males in 50-kb window across each chromosome. b) The proportion of three genotypes for SNP sites in SLR on chromosome 5 of *H. sajori*. Each row corresponds to one female or male individual. In females, most SNP sites were heterozygous. c) LD plot for chromosome 5. Cells above the diagonal show results for all *H. sajori* individuals, including both males and females; below the diagonal only male individuals were included. The colors represent the second highest *R*^2^ values in 100-kb windows, in order to indicate regions of the chromosome in which LD values differ. d and e) Circos plot showing chromosomal and whole-genome features for *H. sajori* (d) and *H. intermedius* (e). From outer to inner, the tracks indicate: (I) chromosome sizes in Mb; (II) mean log2 F:M SNP densities (calculated in 50-kb sliding windows); (III) mean log2 F:M coverage ratios (also in 50-kb sliding windows) across each chromosome.

We mapped whole-genome resequencing data from 24 *H. sajori* individuals of each sex, sampled from two natural populations (detailed in supplementary table S8, Supplementary Material online) to our male genome assembly for this species (supplementary table S9, Supplementary Material online). This revealed a large genome region in which many variants show high *F*_ST_ between the two sexes (Fig. 2a). In this region, most single nucleotide polymorphisms (SNPs) were heterozygous in female individuals but homozygous in males (Fig. 2b). These results suggest that *H. sajori* has female heterogamety with a W-linked region on HsaChr5. LD was also pronounced and decayed very slowly across this region (Fig. 2c; supplementary fig. S7, Supplementary Material online), consistent with a low recombination rate in both sexes, or possibly just in females, the heterogametic sex. This extensive region, from 11.5 Mb to 37.2 Mb (about two-thirds of chromosome 5, whose total assembly length is 40 Mb), can therefore be described as a ZZ/ZW region, hereafter referred to as the SLR. In female heterogametic systems, highly degenerated W regions have genomic coverage half that in males, but our coverage analysis (using 50 kb windows) detected no such regions (Fig. 2d; supplementary fig. S8, Supplementary Material online). Furthermore, SNP densities were higher in females than males in the SLR (supplementary fig. S9, Supplementary Material online). Both these results suggest that most Z-linked genes are still present in the W haplotype (Fig. 2d). The SNPs in individuals of known sex were used for phasing and other analyses described below, including analysis of degeneration.

The SLR is very different in *H. intermedius*. Whole-genome association analysis of females and males from natural populations (12 individuals of each sex, sampled from two natural populations, see supplementary tables S8 and S9, Supplementary Material online) detected no SLR (or variants) on HinChr5, using our female *H. intermedius* reference genome sequence for mapping. *F*_ST_ analysis between the sexes showed no large region of male–female differentiation anywhere in the genome (supplementary fig. S10, Supplementary Material online), and a search for strongly sex-associated variants found only eight SNPs, two insertions, and one deletion; these 11 variants were all within a 140 kb region of chromosome 14 (HinChr14; supplementary fig. S11, Supplementary Material online). This species probably has male heterogamety, as these variants are homozygous in all 12 females, and heterozygous in most males (supplementary fig. S11, Supplementary Material online). Given the incomplete male heterogamety observed, we also applied a kmer-based approach, using the raw sequencing reads. We identified 31-bp kmers that were present in most males (≥11 of 12 individuals) but absent in all 12 females. The reads containing these male-specific kmers (172 kmers in total) were assembled into nine contigs, with a mean length of 842 bp (they ranged from 489 to 1,029 bp). Alignment to our female *H. intermedius* reference genome yielded five male-associated contigs covering a 4,494 bp region on HinChr14 (positions: 12,078,875–12,216,651), within which the 11 most strongly sex-associated SNP/indels were also located (supplementary fig. S12, Supplementary Material online). This region includes five genes. Neither coverage nor SNP density analyses detected any genomic regions differing between males and females (Fig. 2e).

As *H. intermedius* and *H. sajori* are more closely related than either is to other species in the same genus, the large W-linked region on HsaChr5 in *H. sajori* could have evolved after its split with *H. intermedius*, and the small HinChr14 Y-linked region may be the ancestral state, or the derived and ancestral states could be the other way around. To distinguish between these possibilities, we compared W-Z sequence divergence with that between the Y and X-linked alleles, as described in the section below, entitled “Sex chromosome evolutionary strata”, after first describing the phased assembly of the W-linked region. Comparing their levels of genetic degeneration is less helpful, because the HinChr14 region includes so few Y-linked genes that degeneration is not expected. However, the partial degeneration of the HsaChr5 W-linked region described above suggests that it evolved recently.

### Assembly of the W Chromosome

As described above, the *H. sajori* SLR of chromosome 5 (HsaChr5) proved to be heterozygous in females. To study this region, we de novo assembled 24.71 Gb of PacBio HiFi reads from a female (supplementary table S2, Supplementary Material online) into partially phased contigs. This involved using female-specific SNPs (the sex-linked SNPs described above) to classify haplotigs (contigs that come from the same haplotype; in an unphased assembly, a contig can include alleles from different parental haplotypes in a diploid or polyploid genome). To identify HsaChr5 contigs as either Z- or W-linked group, we excluded contigs whose phase average confidence was less than 99.77% (see Methods). We could then anchor the contigs in a “pseudo-W chromosome” with sequences ordered as in the Z chromosome assembled from the male *H. sajori* genome described above.

### Candidate Sex-Determining Genes

Members of transforming growth factor-β (TGF-β) signaling pathway, such as *amh*, *amhr2*, and *gsdf*, are known to induce gonadal masculinization and are frequently found as master sex-determining genes across teleost fishes (reviewed by Kitano et al. 2024). In the ∼140 kb *H. intermedius* Y-linked region, the anti-Müllerian hormone, *amh*, gene is annotated at HinChr14:12,194,785–12,197,067, and two of the 11 sex-linked variants in this species are located upstream of *amh* (between 809 and 4,546 bp from its start). In *H. sajori*, the anti-Müllerian hormone receptor type II gene, *amhr2*, is located in the middle of the sex chromosome, between HsaChr5 19,993,620 and 20,000,291 bp. We retrieved Z- and W-linked alleles corresponding to protein-coding *amhr2* gene sequences in *H. sajori*, and its ortholog in its close outgroup species, *H. intermedius*, which differs by 11 amino acid changes from both, while the *H. sajori* Z- and W-linked alleles differ at five amino acids (supplementary fig. S13, Supplementary Material online).

### Chromosome Rearrangements and Centromeric Positions

Chromosome rearrangements, including inversions and sex chromosome-autosome translocations, can cause expansion of nonrecombining regions. Both sequenced halfbeak species have 20 chromosomes, fewer than the 24 in the medaka (*O. latipes*) karyotype. After the whole-genome duplication event and major inter-chromosomal rearrangements in the teleost ancestor, *O. latipes* is thought to preserve its ancestral karyotype with 24 chromosome pairs for more than 300 M year (Kasahara et al. 2007; Nakatani and McLysaght 2017). To test whether the extensive nonrecombining region on the *H. sajori* sex chromosome (HsaChr5) was formed by chromosome rearrangements, relative to this karyotype, we compared the locations of all protein sequences annotated in *H. sajori* with those in the medaka to find collinear blocks (Fig. 1b). Our collinearity analysis indicated four major inter-chromosomal rearrangements in *H. sajori*. The four largest *H. sajori* chromosomes, HsaChr1–HsaChr4, each appear to be derived from translocations involving two chromosomes. In contrast, the HsaChr5 chromosome is syntenic with OryChr7, with no sign of translocations (Fig. 1b).

As pericentromeric regions with rare recombination are sometimes associated with evolution of SLRs, we used QuarTeT analysis (see Methods) of our whole-genome assembly data to predict centromere positions (supplementary fig. S4 and supplementary table S6, Supplementary Material online) and to test whether HsaChr05 is (sub)metacentric. This analysis suggested that the centromere on HsaChr5 is located at the left of this chromosome’s assembly, between 1.18 Mb and 2.33 Mb (supplementary fig. S4 and supplementary tables S5 and S6, Supplementary Material online). CentIER analysis detected an overlapping region, from 1.05 to 5.54 Mb (supplementary table S6, Supplementary Material online). Both analyses indicate that HsaChr5 is acrocentric, and its SLR (starting at 11.5 Mb) is clearly not pericentromeric.

### Accumulation of Repetitive Sequences

**Fig. 3.**
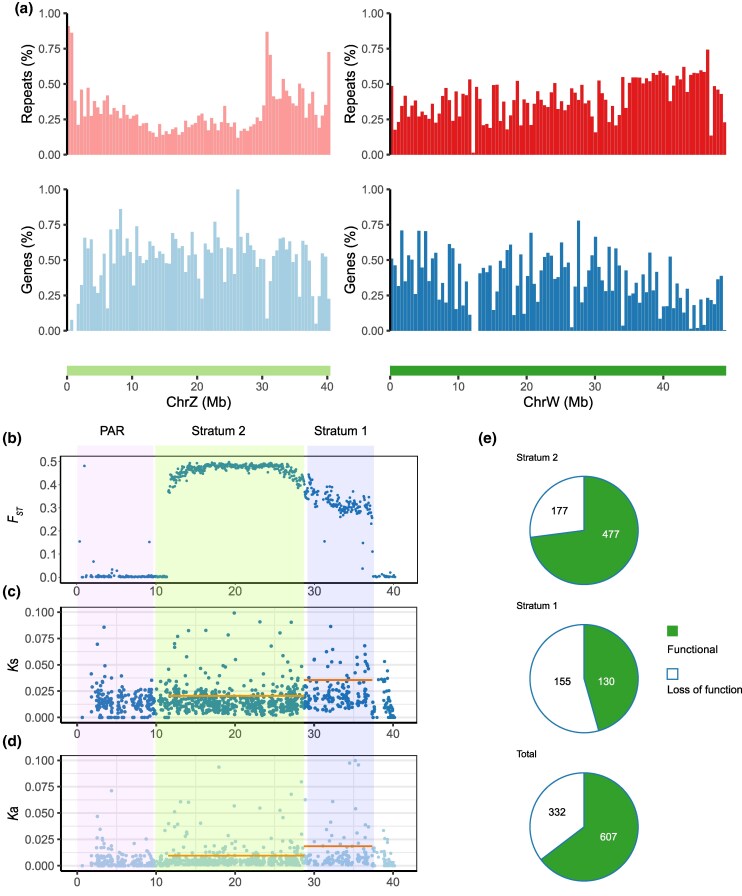
Genomic features of Z/W chromosome and evidence for two evolutionary strata in the *H. sajori* ZW pair. a) The gene and total repetitive sequence densities of the *H. sajori* Z and W chromosomes. b) Mean *F*_ST_ values between females and males in 50-kb window across HsaChr5. c and d) Divergence between protein-coding sequences in the Z- and W- linked regions, and the other parts of chromosome 5, quantified by synonymous site divergence (*K*s, (c)) and nonsynonymous site divergence (*K*a, (d)). *K*a and *K*s values are shown for each gene and their positions in the Z chromosome haplotype of chromosome 5 are shown in megabases (Mb). e) Classification of genes in the *H. sajori* W-linked region by their inferred functionality, Stratum 1, Stratum 2, and the total counts for SLRs were displayed separately (the blank-filled section, genes showed loss of function features; the solid-filled section, genes still functional in ChrW).

Highly degenerated W/Y chromosomes often show size differences from their Z/X counterparts (heteromorphism), and such size differences tend to reflect their evolutionary age. While mammalian Y chromosomes such as the human Y have lost many genes (Skaletsky et al. 2003) and have often shrunk (as this degeneration permits deletions), some plant Y chromosomes have not reached this profound degeneration stage, but have expanded by accumulating repetitive sequences, including transposable elements (as reviewed by Bergero and Charlesworth 2009; Rodríguez Lorenzo et al. 2018; Sacchi et al. 2024 ). The W chromosome in our female *H. sajori* assembly has a total length of 49,214,037 bp, more than 9 Mb larger than the Z (40 Mb). This probably reflects its recent evolution (see also below), with early rapid accumulation of repetitive sequences after recombination stopped. Repeats have indeed accumulated on the W (Fig. 3a). Its estimated repeat sequence densities (proportions occupied by repetitive sequences) in 500 kb sliding windows average 40.56% versus 30.97% for the Z (supplementary table S10, Supplementary Material online), which is similar to the autosomal value of 29.82%. Of the sequences accumulated most highly on the W, the predominant types are DNA transposable elements and long terminal repeat (LTR) retrotransposons, respectively, constituting 19.50% and 12.5% of the entire W. Both strata of the W (defined by the W-Z sequence divergence estimates described in the next section) have higher repeat densities than those of the Z (though the older Stratum 1 has considerably higher values on both sex chromosomes: W 52.27% and Z 42.63% vs. W 35.83% and Z 19.93% in Stratum 2). For Stratum 1, the W accumulated more LTR retrotransposons (17.82%) than the Z (11.36%), while the DNA transposon proportions are similar (Z: 23.64%; W: 23.59%). For Stratum 2, both LTR retrotransposons and DNA transposons are enriched on the W. In *H. intermedius*, HinChr5 has an estimated 33.05% repetitive sequence content, no higher than the autosomal mean (36.35%), but similar to the Z of *H. sajori*.

### Sex Chromosome Evolutionary Strata

To distinguish whether the HinChr14 Y-linked region or the large HsaChr5 W-linked region is most likely to be ancestral, we estimated synonymous site divergence (*K*s) between the Z- and W-linked haplotypes in *H. sajori*. Across the region inferred above to be sex linked (the SLR), the females have much higher SNP densities in unphased data than males in our natural population samples (supplementary fig. S9, Supplementary Material online), and the pattern of density values across the assembly suggested that this species might have two evolutionary strata, requiring separate *K*s estimates. Changepoint analysis of these SNP data indeed detected a change at 28.72 Mb. The mean W-Z *K*s values estimated from our two phased haplotypes confirm two strata (supplementary fig. S15, Supplementary Material online; Table 1). The older stratum (Stratum 1) occupies the region from 28.72 to 37.32 Mb and contains an estimated 285 PCGs (gene density 33 per Mb), while the younger stratum (Stratum 2, which includes 654 PCGs) spans the region between 11.55 and 28.72 Mb and has a slightly higher gene density (38.1 genes per Mb); although it is closer than Stratum 1 to the centromere, these gene densities are both high, consistent with the conclusion above that neither region is a pericentromeric region with low gene content. The W-Z *K*s values of Stratum 1 genes are low (mean = 0.035; see supplementary fig. S15, Supplementary Material online), but significantly larger than for Stratum 2 genes (mean = 0.020; Mann–Whitney *U* test, *P* = 4.8e−11; see supplementary fig. S15, Supplementary Material online; Fig. 3c and Table 1). Pseudo-autosomal region (PAR) genes are expected to have zero divergence (or, in a single pairwise comparison, as with our two phased haplotypes, a low value reflecting the population’s nucleotide diversity). Our estimated value of 0.016 for PAR genes is significantly lower than in Stratum 1 (by a Mann–Whitney *U* test, *P* = 1.7e−11), but did not differ significantly from that for Stratum 2 genes. Nonsynonymous divergence (*K*a) of Stratum 1 genes (mean = 0.018) is significantly higher than in Stratum 2 (mean = 0.009; Mann–Whitney *U* test, *P* = 0.00024) or the PAR (mean = 0.005; Mann–Whitney *U* test, *P* = 2.7e−10). The Stratum 2 value is also significantly higher than for PAR genes (by a Mann–Whitney *U* test, *P* = 1.6e−8) (supplementary fig. S15, Supplementary Material online; Fig. 3c and Table 1). Despite the modest W-Z *K*s values, high LD across both strata indicates that SNPs in both are completely sex linked (supplementary fig. S16, Supplementary Material online). The high *F*_ST_ between the sexes also supports complete sex-linkage and is consistent with a young system, as pronounced differentiation evolves more quickly than high divergence (Toups et al. 2019).

**Table 1:** The *H. sajori* chromosome 5, showing boundaries between the different regions, and their overall properties, including W-Z divergence estimates (*K*a and *K*s) for the two inferred evolutionary strata in the fully SLR

Region	Position in the *H. sajori* Z assembly		Gene density (genes per Mb)	Number of genes compared^a^	Mean divergence (W-Z or inter-species)		Nonfunctional on W (LoF)	
	Start (bp)	End (bp)			*K*a	*K*s	Number of genes	Percentage
PAR	0	11,556,404	24	258(226)	0.005	0.016		
SLR								
Stratum 2	11,556,405	28,723,220	38	654(553)	0.009	0.020	177	27.06
Stratum 1	28,723,221	37,328,662	33	285(154)	0.018	0.035	155	54.39
Inter-species divergence values for the two halfbeaks	–	–	–	1,228(935)	0.018	0.048	–	–

The inferred position of the centromere is within the region where the PAR is assigned, although part of this should be nonrecombining; the centromere lacks genes, and is thus not included in the divergence estimates shown. Five large inversions were detected in the SLR, at the positions given in supplementary table S11, Supplementary Material online. Four are within Stratum 2, and one (much larger than the others) spans the Stratums 1–2 boundary.

^a^The divergence values were estimated using gene pairs identified as RBHs (also remove any many-to-one reciprolog relationships) in protein sequence comparisons for *H. intermedius* versus *H. sajori* or ChrZ-W. Numbers indicate genes used for protein sequence comparisons. Values in parentheses indicate gene that were identified as RBH and used to calculate the *K*a and *K*s. Nucleotide sequence of homolog gene pairs were aligned using MACSE while allowing for the occurrence of frameshifts. Hence, both intact and defective sequences were used here.

The mean *K*s value between *H. sajori* and *H. intermedius* is 0.048, distinctly higher than between the W and Z in the older *H. sajori* stratum (Table 1; supplementary fig. S17, Supplementary Material online). Where the same genes could be compared, most individual genes had lower *K*s values for W-Z divergence than for inter-species divergence (supplementary figs. S18 and S19, Supplementary Material online). In sliding windows of 50 genes, the W-Z divergence was consistently lower than the inter-species value across the HsaChr5 chromosome (supplementary fig. S20, Supplementary Material online). To further assess the evolutionary history of the *H. sajori* sex chromosomes, we examined the relationship of Z/W gametolog pair sequences in *H. sajori* to their orthologs in *H. intermedius*, using other species to root the tree (see Methods). If the *H. sajori* ZW system evolved after the split with *H. intermedius*, as suggested by the *K*s estimates, its Z and W sequences should cluster with each other, with *H. intermedius* as a close outgroup. Of the 190 orthologous single-copy genes shared by the ZW of *H. sajori*, *H. intermedius*, and other six halfbeaks, the topologies of 164 (86%) gene trees support this scenario. Among the genes supporting alternative scenarios with an ZW system present in the ancestor of *H. sajori* and *H. intermedius*, 20 (11%) *H. intermedius* sequences coalesce with those of the *H. sajori* Z, suggesting loss of the W allele, while 6 (3%) are consistent with loss of an ancestral Z allele (supplementary fig. S21, Supplementary Material online).

The estimated divergence time between *H. sajori* and *H. intermedius* is 4.2 Mya, using a phylogenetic tree based on four-fold degenerate sites of single-copy genes with six other teleost species (calibrated using the following divergence time estimates: *Oreochromis niloticus—O. latipes* 81–96 Mya and *Danio rerio—Cynoglossus semilaevis* 180–250 Mya) (supplementary fig. S22, Supplementary Material online). The older *H. sajori* Stratum 1 is estimated to have evolved about 3 Mya. As noted above, the inferred XY-linked region of *H. intermedius* includes very few completely sex-linked gene pairs (gametolog pairs). Therefore, a reliable divergence estimate could not be obtained to compare its age with that of the ZW system.

### Sex Chromosome Inversions

Mapping phased HiFi long reads from the female *H. sajori* genome assembly to that of the male detected five inversions >1 kb within the W-linked region (supplementary table S11, Supplementary Material online). A 12.83 Mb inversion (from 23,178,158 to 36,004,388 bp using HsaChr5 as the reference) was supported by five PacBio HiFi reads. The other inversion larger than 1 Mb spans 18,807,482–20,518,460 bp of HsaChr5; this 1.71 Mb inversion is supported by eight high-quality reads. To infer the ancestral order of chromosome 5, we compared the genome sequences of the HsaChr5 Z with its homolog in the outgroup, *H. intermedius* (HinChr5). A synteny plot (supplementary fig. S23, Supplementary Material online) indicated that the *H. sajori* Z arrangement is ancestral and the W has undergone inversions. These W inversions, especially the large ones, may have contributed to suppressing recombination with the Z or could have evolved on the W after it stopped recombining with the Z. The largest one spans the Stratum 1–2 boundary, consistent with the possibility that it established complete linkage between the two strata. The other four are all within Stratum 2; they cover most of this 17 Mb region and could have been involved in its suppressed recombination that created complete sex-linkage of genes in the region.

### Degeneration of Protein-Coding Sequences in the *H. sajori* W-Linked Region

To evaluate the extent of W chromosome degeneration in *H. sajori*, we combined phased PacBio HiFi reads with our short-read data from multiple individuals of known sex to ascertain a large set of W-specific variants (SNPs, small indels, and structural variants (SVs)). Within the SLR, 190,203 SNPs and 30,522 small indels (≤30 bp) were detected exclusively on the W, consistent with a lack of recombination, while 39,062 SNPs and 6,794 small indels (≤30 bp) were detected on the Z. The phased HiFi long reads for the W yielded 6,192 W-specific large deletions or insertions (between 30 bp and 500 kb), suggesting the possibility of degeneration allowing large deletions in some parts of the SLR (supplementary fig. S24, Supplementary Material online).

We also mapped the annotated Z-linked protein-coding sequences to the W chromosome reference genome to estimate the proportion of Z-linked genes that still have intact open reading frames on the W. This revealed considerable degeneration. Of 939 genes identified in the HsaChr5 Z SLR, 332 (35.4%) are nonfunctional on the W (Fig. 3e and Table 1); of these, the coding sequences of 80 genes exhibit significant sequence loss (query coverage <0.5 or/and identity <0.6), 30 have lost more than one exon, 151 have lost their start codons and/or are disrupted by premature stop codons or frame-shift mutations; the remaining 71 have mutations in splice donor or/and acceptor regions, and their W-linked alleles may also have lost their functions (supplementary fig. S24 and supplementary table S12, Supplementary Material online). Consistent with two strata of differing ages, the proportion of nonfunctional genes is higher in the older Stratum 1 (54.4%) than the younger one (27.1%) (Fig. 3e and Table 1).

### Testing for Dosage Compensation in *H. sajori*

To investigate whether Z-linked genes show dosage compensation in response to the loss of function of their W-linked counterparts in *H. sajori* females (which will sometimes be detrimental when hemizygous, or functionally so), we obtained RNA-seq data from two somatic tissues, muscle and liver (from two females and two males for each tissue; supplementary table S13, Supplementary Material online). In the absence of dosage compensation, the Zfemale/ZZmale expression ratio is expected to be 0.5. If dosage compensation has evolved, this is likely to initially involve higher expression of Z-linked genes, either specifically in females or in both sexes. Non-sex-specifically increased expression, affecting Z-linked genes in both sexes, would leave the Zfemale/ZZmale expression ratio unchanged at 0.5, but result in males’ Z-linked expression being higher than that of autosomal genes; males (with the ZZ AA genotype) would therefore have Z:A expression ratios above 1. In contrast, female-specific doubling of expression from females’ single Z-linked alleles would leave males’ ZZ and AA total expression levels unchanged.

**Fig. 4.**
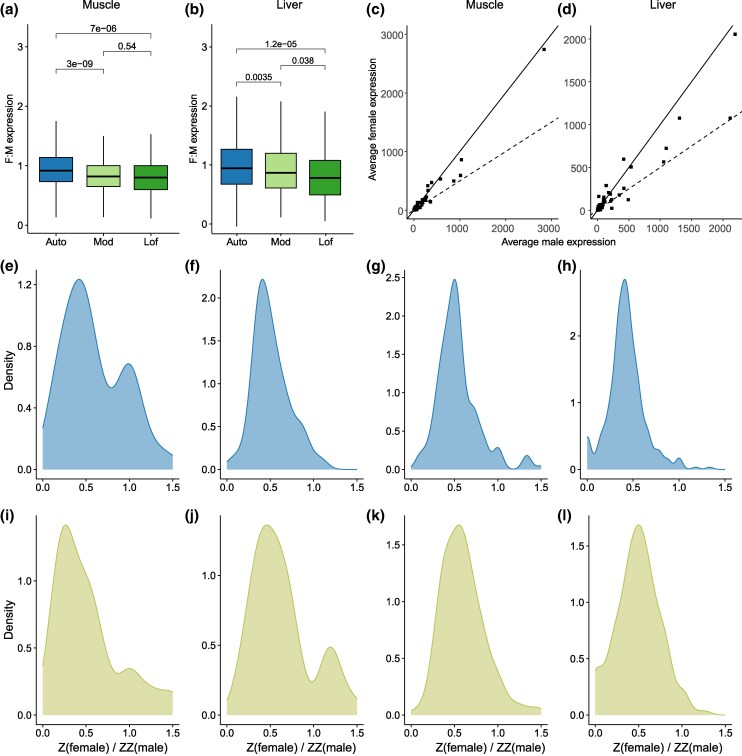
Gene expression evidence for dosage compensation of a minority of sex-linked genes in *H. sajori*. a and b) Comparison of gene expression level estimates according to their chromosome and mutation effect in two tissues. Autosomal (Auto) genes are shown, and genes within the SLR that displayed loss of function effect (Lof) and the remaining sets (Mod) are shown separately. c and d) Average normalized gene expression values (TPM) in females versus males (two of each sex) for sex-linked genes that displayed Lof. The solid line shows the expectation under equal female and male expression, and the dashed line shows the expectation for female expression being equal to one-half of male expression. e–l) Frequency density plots of transcript abundances of single Z-linked alleles in females (labeled Z female) compared with the two copies in males (ZZ male) for sex-linked genes in muscle (e–h) and liver (i–l). The data are partitioned according to the relative transcript abundances of W compared with Z copies in females, using the same W:Z in female values as those in the *x* axis of the plots above. Four categories are shown: (e) and (i) show genes with W/Z values = 0, (f) and (j) show values >0 but <0.256, (g) and (k) show values defined by 0.256 ≤ W/Z < 0.667, and (h) and (l) show the highest W/Z values ≥0.667.

In both somatic tissues of *H. sajori* that we studied, overall expression levels of autosomal genes were almost equal in females and males, while the mean female:male (F:M) expression ratio estimates were slightly, but significantly, below 1 for sex-linked genes, consistent with the evidence above for some genes being degenerated, and suggesting that deleterious mutations in the W-linked alleles might have reduced overall expression levels in females. Figure 4a and b shows the results for the Z-linked genes with nonfunctional W copies, and for other, still apparently functional, ones, respectively. We found no significant difference in expression levels in males between autosomal genes and SLR ones (Mann–Whitney *U* tests, *P* = 0.54 in muscle and *P* = 0.31 in liver), indicating that the Z-linked genes are not upregulated in males (supplementary fig. S25, Supplementary Material online).

Testing for compensation yields the clearest conclusions if one studies W-linked genes with loss of function variants, because there is no possibility that transcript abundance differences between the sexes might simply reflect genes with sex-biased expression. Our analysis of such genes suggests that a few genes may be dosage compensated in both tissues (Fig. 4c and d). As most Z-linked genes in our systems have counterparts on the W, we distinguished Z- and W-specific RNA reads in females using the sex-specific SNPs ascertained as described above. We compared Z-linked expression values in females (with a single Z) with those from the ZZ males’ two Z-linked alleles. The overall ratio (Z female/ZZ male) for sex-linked genes was near 0.5 for both functional genes and those with loss-of-function mutations, and there was no significant difference between these two categories (supplementary fig. S27, Supplementary Material online). Then we divided the sex-linked genes into four categories according to their W:Z expression ratios in females (Fig. 4). Most genes showed unimodal distributions of the Zfemale/ZZmale ratio, with the peak near 0.5, consistent with being uncompensated (Fig. 4f–h, k, and l). However, the categories of genes with extremely low W-linked allele expression (Fig. 4j), or no detectable expression (Fig. 4e and i), showed a “shoulder” suggesting some bimodality, with a clear second peak value near 1 (Hartigan’s dip test of unimodality, *P* < 0.05 for Fig. 4e and i; supplementary tables S14–S16, Supplementary Material online). Thus, within this recently evolved W-linked region, a few Z-linked genes appear to show upregulated Z-linked expression specifically in females; the W-linked alleles of these genes also have very low expression (supplementary fig. S28, Supplementary Material online). As discussed below, this might reflect an early stage in the evolution of dosage compensation.

## Discussion

### Evolution of a Young Sex Chromosome Involving a Turnover Event in Which Chromosome 5 Acquired a Femaleness Factor De Novo

Sex chromosome turnover events, such as the change in the sex chromosome documented in these fish, offer clear examples in which a new sex chromosome must have evolved from an autosome pair. Studies of such changes in fish (reviewed by Pan et al. 2019) show that such turnovers often involve insertion of a duplicate gene copy into an autosomal region, though the progenitor and duplicate may sometimes both be in different regions of the ancestral sex chromosome; alternatively, mutations can create new sex-determining genes from pre-existing genes. Such events must often create a small fully SLR, or single sex-linked gene, and the surrounding genome region might be expected to continue to recombine. It is therefore surprising that the young sex chromosome in *H. sajori* (that our results suggest evolved within the past 3 or 4 million years) has a large fully SLR. Moreover, this region has clearly evolved by recombination suppression events, as it includes inversions and two evolutionary strata (see above), the older of which (Stratum 1) is separated from the centromere by about 27 Mb (Table 1; supplementary table S11, Supplementary Material online), making it unlikely to be a pericentromeric region that might have formerly recombined very rarely and subsequently completely stopped recombining.

Although some other young SLRs have been well studied, these are often situations in which sex-linkage arose as a consequence of a sex chromosome-autosome fusion in a species in which one sex does not undergo meiotic recombination, so that the former autosome immediately becomes completely sex linked. Such fusions have repeatedly occurred in *Drosophila* species, creating neo-sex chromosomes, such as the *Drosophila miranda* neo-Y, which evolved only approximately 1.5 million years ago (Bachtrog 2005; Zhou and Bachtrog 2012). Most genes on this neo-X are still detectable on this neo-Y, but about half are estimated to have lost their functions (Nozawa et al. 2021). In species in which both sexes undergo recombination, however, much of the former autosome may continue to recombine, except near the fusion point, where recombination may stop, as reported for the XYY cytotype of *Rumex hastatulus* (Rifkin et al. 2021; Sacchi et al. 2024). After a Y-autosome fusion in the stickleback species *Gasterosteus nipponicus* about 1.5–2 Mya, no substantial neo-Y chromosome degeneration is yet detectable, though nucleotide sequences have started to diverge, particularly near the fusion (Kitano et al. 2009; Yoshida et al. 2014). In *Gasterosteus wheatlandi*, recombination suppression after a fusion about 1 Mya is more extensive (covering a region of ∼16.4 Mb) and neo-Y chromosome degeneration is greater than in *G. nipponicus* (Sardell et al. 2021). The HsaChr5 sex chromosome, however, is syntenic with OryChr7 and shows no signs of a chromosomal translocation that could have been involved in the evolution of suppressed recombination.

Our W-Z *K*s estimates and gene tree analysis suggest that the 26 Mb fully W-linked region in *H. sajori* evolved after the split from *H. intermedius*, with Z-W divergence in the older *H. sajori* stratum only 73% of the inter-species divergence. It is therefore unlikely that the *H. sajori* ZW chromosome pair represents the ancestral state of the two species studied here. Moreover, if it does, and the small male-determining region in *H. intermedius* evolved in a very recent turnover event, its homologous chromosome might be expected to retain signs of having formerly been a sex chromosome, such as high repeat-richness; accumulated repetitive sequences will disappear after a turnover in which a different chromosome gains a new sex-determining factor, at a speed depending on how deleterious the sequences are, but should remain for a considerable time. However, our results showed that HinChr5 had a similar repetitive sequence density (33.05%) to the estimates for the *H. sajori* autosomes (36.35%) or Z (30.97%).

Sex chromosome turnovers have been found in many taxa, either maintaining the ancestral heterogamety or changing it (XY to ZW, or vice versa; see Bachtrog et al. 2014; Gammerdinger and Kocher 2018; Jeffries et al. 2018), as in the species studied here. Our divergence estimates suggest that the *H. sajori* ZW state is recently derived. The state in *H. intermedius*, with a maleness determiner defining a Y-linked region on a different chromosome, could reflect the ancestral state, which would be consistent with the idea that ZW systems generally evolve from male heterogamety, and more rarely the other way around (Bull 1983; Sardell and Kirkpatrick 2020), or it could reflect a turnover from an ancestral XY or ZW state; gene trees including the other species might shed light on this, though the unknown ancestor may no longer exist. At present, we cannot distinguish between these possibilities.

### Candidate Sex-Determining Genes

Master sex-determining genes have now been identified in many teleost fish. Among them, genes related to TGF-β signaling are widespread across teleost groups, as reviewed by Pan et al. 2021, and are not carried on homologous chromosomes (Kitano et al. 2024). Genes belonging to the TGF-β superfamily have become involved in sex determination through different mutational changes, including SNP mutations, such as a missense SNP mutation of *amhr2* in tiger pufferfish, *Takifugu rubripes* (Kamiya et al. 2012), or copy number changes such as a tandem duplication of *amhy* in the Nile tilapia, *O. niloticus* (Li, Sun, et al. 2015), or duplication of the autosomal *amh* gene in northern pike, *Esox lucius* (Pan et al. 2019). The sex-determining regions are on nonhomologous chromosomes in *H. sajori* and *H. intermedius*, but we found TGF-β signaling pathway candidate sex-determining genes in the SLRs of both species. One candidate sex-determining gene encodes the *anti-Müllerian hormone*, *Amh*. This glycoprotein member of the TGF-β superfamily plays an important role in Müllerian duct regression during male sexual differentiation in tetrapod vertebrates (Münsterberg and Lovell-Badge 1991; Josso et al. 2006). Part of *amh* becomes active after proteolytic cleavage and binding to the Anti-Müllerian hormone receptor type II, *amhr2* (Oliveira et al. 2023).

In *H. sajori*, both the Z and W carry intact *amhr2* alleles, with no copy number difference. The W copy has five missense mutations compared with the Z and 12 missense mutations compared with the *H. intermedius* sequence (also on chromosome 5), which may be a suitable outgroup to infer the functional state. These changes could affect sex determination, with the W-linked copy possibly being nonfunctional, but it is difficult to test this in these nonmodel species. However, *H. sajori* has female heterogamety and must therefore differ from the system in *H. intermedius*, and its chromosome 5 does not carry an *Amh* gene, so its sex determination system cannot be derived simply from that in *H. intermedius*, even though the TGF-β signaling pathway appears to be involved in both species.

### Spreading of Recombination Suppression

A lack of recombination is not a direct consequence of carrying sex-determining genes and has no essential role in sex determination (reviewed in Charlesworth 2023). Compact sex-determining regions can be maintained within recombining genomic segments, as in *T. rubripes* and several other fish species (Reichwald et al. 2015; Ieda et al. 2018; Nacif et al. 2023). *H. intermedius* may be in this category. How and why the large region with recombination suppression arose in *H. sajori* is thus likely to help understand such changes. As already mentioned, our findings exclude cessation of recombination due to a chromosome fusion, or a location in a pericentromeric rarely recombining region, as inferred in the plants *R. hastatulus* (Rifkin et al. 2021), *S. latifolia* (Bergero et al. 2013; Filatov 2024), and *Vasconcellea parviflora* (Iovene et al. 2015), and possibly *C. papaya* (Gschwend et al. 2012). More likely, one or more of the five inversions larger than 1 kb that we detected within the SLR, two of which were larger than 1 Mb, was involved in recombination suppression. Using *H. intermedius* as an outgroup shows that ChrZ has the ancestral arrangement, while the W has undergone inversions. Although these may have suppressed recombination between the W and Z, especially the largest one, a ∼12.86 Mb inversion which spans the Stratum 1-2 boundary (Table 1; supplementary fig. S23 and supplementary table S11, Supplementary Material online), the four within Stratum 2 could have fixed in the W chromosome population after complete W-linkage evolved. The *amhr2* gene is within a ∼1.71 Mb inversion within Stratum 2.

### Partial W Chromosome Degeneration in *H. sajori*

Genetic degeneration appears to have started evolving in *H. sajori* (Table 1). Based on the estimated divergence times between the Z and W for the two strata (3 Mya for Stratum 1, and 1.8 for Stratum 2), we estimate that 18.1% and 15.1% of genes have degenerated per MY, respectively. For comparison, ∼40% of the genes on the *D. miranda* neo-Y have lost their functions within 1 million years (Zhou and Bachtrog 2012), or, assuming 10 generations per year, 4% per million generations. In the tongue sole (*C. semilaevis*), 64.9% of genes have been lost in the past 30 MY, a degeneration rate of 2.2% per MY (Chen et al. 2014) and 5.5% per million generations, assuming a generation time of 2.5 years (Song et al. 2021). The generation time of *H. sajori* is about 1.5 years (Chen 1984), suggesting a much faster degeneration rate of 27.2% per million generations in Stratum 2 and 22.7% in Stratum 1. The rate of degeneration is expected to be fastest early in the degeneration process and to decrease over evolutionary time (Bachtrog 2008; Engelstädter 2008), and our study species may be in the early, rapid phase.

### Evolution of Dosage Compensation in a Young ZW System

Given the evidence for degeneration, we tested whether dosage compensation occurs in this young system. Complete dosage compensation may not always evolve, even in species in which high degeneration has been documented, such as the W chromosomes of birds reviewed by Mullon et al. 2015. Whether, as Y- or W-linked genes degenerate, their X- or Z-linked alleles have become upregulated in the heterogametic sex is unclear in many species, and it is often unclear whether any up-regulation is specific to the heterogametic sex versus non-sex-specific (Livernois et al. 2012). Incomplete dosage compensation of sex-linked genes has been claimed to be common in fish (Chen et al. 2014; White et al. 2015; Hale et al. 2018). However, it is important to establish whether there is evidence for degeneration, and to assess dosage compensation using genes that are hemizygous in the heterogametic sex (to avoid confounding with sex differences in expression), and these criteria are not satisfied for all species.

As overall mean expression levels of sex-linked genes in female *H. sajori* (including genes with W-linked alleles) are slightly lower than in males in both the somatic tissues studied (Fig. 4a and b), we infer that W-linked alleles at some loci have lower expression than their Z-linked counterparts. This could reflect degeneration caused by the loss-of-function mutations such as frameshifts or premature stop codons in coding sequences; it could also reflect deleterious mutations in promoters and cis-regulatory elements in the W-linked copies (Charlesworth and Charlesworth 2000; Muyle et al. 2012). Moreover, the sex-linked genes with no detectable W expression include genes in which loss of function mutations have been identified, as well as ones with mutations that will render them nonfunctional (supplementary tables S15 and S16, Supplementary Material online); both these categories are functionally hemizygous in females, and expression of just these Z-linked alleles gives a Zfemale/2Zmale ratio close to 0.5, similar to the overall female/male ratio for all fully sex-linked genes. However, a few sex-linked genes with extremely low or undetectable W-linked allele expression show upregulated Z-linked expression, leading to Zfemale/2Zmale ratios close to 1. This was also observed in two unrelated plants *S. latifolia* and *Coccinia grandis* (Papadopulos et al. 2015; Fruchard et al. 2020). It might reflect a nascent form of dosage compensation system of individual sex-linked genes, or simply transcriptional buffering in gene networks, which may maintain transcript levels despite low genomic dosage or alleles with impaired expression. For example, partial dosage compensation for gene duplications or deletions has been documented for autosomal genes in *Drosophila* (Malone et al. 2012), maize (Guo and Birchler 1994), and yeast (Muenzner et al. 2024). However, this process appears not to explain the findings in expression studies in *S. latifolia*, and the nature of any dosage compensation in this plant remains uncertain (Muyle et al. 2012; Krasovec et al. 2019). Our study suggests that compensation of some degenerated genes may occur in the young *H. sajori* ZW system. Unlike examples of complete and chromosome-wide dosage compensation in placental mammals and *Caenorhabidtis elegans*, this appears to be a gene-by-gene process, as expected for a partially degenerated sex-determining region. If up-regulation automatically occurs when the copy number or expression level of a gene is reduced, as suggested above, it may be difficult to test for dosage compensation in young systems with incomplete degeneration. This will apply to the suggestions outlined in the Introduction that up-regulation of X- or Z-linked genes may occur immediately in the heterogametic sex, as the Y or W alleles degenerate. Tests of the effects of small deletions (as implemented by Krasovec et al. 2019, for example) are required in order to determine whether or not degeneration precedes compensation. Such experiments in *D. miranda* suggested that neo-X-linked genes are unlikely to have been compensated immediately after their neo-Y-linked gametologs were pseudogenized (Ogawa et al. 2023).

## Conclusions

Our study of the sex chromosome system in *H. sajori* shows that the young W-linked region has already undergone several inversions, one or more of which may have led to its extensive fully SLR. The study also confirms the conclusion from studying the *D. miranda* neo-Y chromosome that genetic degeneration of W chromosome can occur rapidly, in a number of generations corresponding to synonymous site divergence of only a few percent, with genes losing function without being lost. Such systems are important for testing the ideas outlined in the Introduction about active processes that involve the simultaneous evolution of genetic degeneration and for testing between the possible forms of nascent dosage compensation. It remains unclear why the sister species, *H. intermedius*, has only a small Y-linked region, and whether the ZW system in *H. sajori* evolved from an ancestral XY system like that in *H. intermedius*. Further studies of the sex-determining systems of other suitable outgroup species are needed to test how many turnover events have occurred among halfbeak species, whether other sex chromosome systems exist in the genus, and, if so, which of these have evolved extensive fully SLRs, and undergone genetic degeneration, and the degeneration rates.

## Materials and Methods

### Sample Collection and Sequencing

This study was done after our serendipitous discovery of the sex-determining region during population genetic work on *H. sajori*. As part of routine genomic screening for SVs (e.g. inversions) and their potential adaptive significance, we employed indirect detection methods such as LDna (Kemppainen et al. 2015) to identify regions of high LD. This revealed an extensive LD block on chromosome 5 in which principal component analysis clustered the individuals into two distinct groups (unlike the three expected under an inversion model). The size of this recombination-suppressed region led us to hypothesize sex-linkage. To test this, we collected and sexed adults during the breeding season and performed whole-genome resequencing, using male and female individuals collected from the nearshore waters of Qingdao, Shandong Province, China, in 2022 September and 2023 April. The results confirmed sex-linkage of this region and revealed female heterogamety (as described in subsection “Identification of SLRs” below). *H. intermedius* was selected as an outgroup for further study of the SLR, because taxonomic keys assigned it as the closest relative of *H. sajori*. As the initial reference individuals of these two species were chosen without considering their sexes, the genome of an *H. sajori* female was sequenced after confirming its heterogamety. A female *H. intermedius* was collected from Weishan Lake, Shandong Province, in 2021 January.

Multiple fresh tissues including muscle, heads, gills, eyes, liver, and hearts were immediately frozen in liquid nitrogen for 30 min and stored at −80 °C until DNA and RNA extraction. High-molecular-weight (HMW) genomic DNA of these three individuals was extracted using the standard phenol-chloroform method from muscles and purified by AMPure XP beads (Beckman Coulter, High Wycombe, UK) for PacBio HiFi read generation. DNA integrity was verified by 1% agarose gel electrophoresis (EPS-600 system, Tanon, Shanghai, China), and concentrations were quantified using a Qubit 3.0 Fluorometer (Thermo Fisher Scientific, Waltham, MA, USA). HMW DNA (>23 kb) was sheared to 15 kbp using the Megaruptor 2.0 system (Diagenode, USA) and processed with the SMRTbell™ Express Template Prep Kit 2.0 (PacBio, 100-938-900, CA, USA) for PacBio HiFi library construction. Libraries underwent blunt-end repair, adapter ligation (SMRTbell Overhang Adapters), and size selection using BluePippin (Sage Science, BLF7510) before sequencing. The libraries were sequenced in circular consensus sequencing (CCS) mode on the PacBio Sequel II system (for one female of *H. intermedius* and one *H. sajori* male) or the PacBio Revio platform (for the *H. sajori* female). HiFi reads were generated from the raw subreads using the CCS workflow (v4.2.0). After removing low-quality reads and adaptors from the raw data, 29.04, 24.52, and 24.71 Gb of clean HiFi data with mean read lengths of 15.09, 15.28, and 14.51 kb were obtained from the male *H. intermedius*, male *H. sajori*, and female *H. sajori*, respectively (supplementary table S2, Supplementary Material online).

For Hi-C library construction, the *MboI* restriction enzyme was used to digest the cross-linked HMW genomic DNA. After 5′ overhang biotinylation and blunt-end ligation with T4 DNA ligase (Thermo Fisher), the DNA was physically sheared into 300–500 bp fragments using the Covaris S220 sonicator (Covaris, USA). The biotinylated DNA fragments were captured using streptavidin-coated magnetic beads (AMPure PB, PacBio, 100-265-900) and processed into Illumina-compatible libraries using the Mate-pair Kit (Illumina). The library quality and concentration were assessed using the Agilent 2100 Bioanalyzer (Agilent, Waldbronn, Germany) and the Qubit 3.0 Fluorometer (Invitrogen, USA), followed by paired-end 150 bp sequencing (PE150) on the Illumina NovaSeq 6000 platform (Illumina, USA). A total of 76.46 Gb and 114.11 Gb of paired-end Hi-C clean reads were generated for *H. sajori* and *H. intermedius*, respectively. Paired-end libraries with a 350 bp insert size were constructed for genome surveys using the VAHTS^™^ Universal Plus DNA Library Prep Kit for Illumina (Vazyme, ND617-022). Genomic DNA was fragmented with FEA Enzyme Mix (Vazyme), end-repaired, and ligated with Illumina-compatible adapters, and the short-fragment library was sequenced on the Illumina NovaSeq 6000 platform (Illumina, USA) to generate PE150 raw data.

RNA sequencing was conducted to generate transcriptome data to predict gene models. Libraries were prepared using the same VAHTS™ kit (ND617-022), which involves RNA fragmentation, cDNA synthesis, and adapter ligation, followed by sequencing on an Illumina NovaSeq 6000 System (Illumina, USA) to generate PE150 raw data.

To ascertain sex-linked variants and regions, 24 males and 24 females of *H. sajori* were collected from nearshore waters of Qingdao and Weihai, Shandong Province, China, and 12 males and 12 females of *H. intermedius* from Weishan Lake and Fuxian Lake, China. Muscle tissue samples from these individuals were stored in anhydrous ethanol for DNA extraction using the standard phenol–chloroform method. DNA extracts were visualized on 1% agarose gels to assess quality (EPS-600 system, Tanon, Shanghai, China) and quantified using a Qubit 3.0 Fluorometer (Thermo Fisher Scientific, Waltham, MA, USA). Whole-genome sequencing libraries with insert size ∼350 bp were constructed, involving end repair, A-tailing, adapter ligation, and polymerase chain reaction (PCR) amplification. The final libraries were sequenced on the DNBSEQ-T7 System (BGI, China) using PE150 chemistry with a mean coverage of ∼10× for each individual.

### Phylogenetic Relationships of the Two Focal Halfbeaks and Their Close Relatives

To infer the phylogenetic relationships among *H. sajori*, *H. intermedius*, and other halfbeak species (Hemiramphidae), the mitochondrial genomes of seven species (*Hemiramphus far*, *H. sajori*, *H. intermedius*, *Hemiramphus dussumieri*, *Hemiramphus limbatus*, *Hemiramphus paucirastris*, and *Hemiramphus quoyi*) plus the zebrafish (as an outgroup) were downloaded from National Center for Biotechnology Information (NCBI) and used to construct a phylogenetic tree. The coding sequences of 13 mitochondrial PCGs from each species were individually aligned using MACSE v10.02 (Ranwez et al. 2011). The optimal partitioning schemes and best-fitting model for each PCG were obtained by PartitionFinder2 v 2.1.1 (Lanfear et al. 2017). A maximum likelihood phylogenetic tree was constructed using IQ-TREE v2.1.3 (Minh et al. 2020) with 1,000 ultrafast bootstraps. Phylograms were modified and visualized using FigTree v 1.4 (http://tree.bio.ed.ac.uk/software/figtree/).

We also estimated phylogenetic relationships using nuclear PCGs. The genomes of our two focal halfbeak species, *H. sajori* and *H. intermedius*, were assembled using PacBio long reads and Hi-C data (as described in the next section). Genome sequences of six other halfbeak species (including *Rhynchorhamphus georgii*, *H. far*, *Hemiramphus archipelagicus*, *Hyporhamphus gernaerti*, *H. limbatus*, and *H. paucirastris*) were assembled based on whole-genome short-read resequencing data. Raw reads were processed to remove adaptors and low-quality reads using Fastp with default parameters. The reads were then assembled into contigs and scaffolds using MEGAHIT v1.2.9 (Li, Liu, et al. 2015) with default parameters and SSPACE v3.0 (Boetzer et al. 2011) with the following parameters “min pairs of 6 to join contigs”. Contigs were anchored into pseudo-chromosomes using RAGTAG, based on chromosome-level assemblies of the male *H. sajori*. These six genomes were further annotated using LIFTOFF v1.6.3 (Shumate and Salzberg 2021) with the annotation of male *H. sajori* as the reference. Orthologs among these halfbeaks (with *O. latipes* as outgroup) were identified using OrthoFinder v.2.3.4 (Emms and Kelly 2019). Coding sequences of those single-copy genes were then concatenated to estimate the phylogeny.

### Genome Assembly

Genomes of female *H. intermedius* and male *H. sajori* were assembled as follows. PacBio HiFi reads were assembled into contigs with Hifiasm v0.18.2 (Cheng et al. 2021) using default parameters. The primary assembly was further purged to remove haplotypic duplication using PURGE_DUPS v1.2.5 (https://github.com/dfguan/purge_dups). Contigs were anchored into chromosomes using JUICER v1.6 (Durand et al. 2016) and 3D-DNA v180114 (Dudchenko et al. 2017). Chromosome-level super-scaffolds were manually checked and corrected by Juicebox v2.17 (https://github.com/aidenlab/Juicebox).

For the female *H. sajori* individual, the PacBio HiFi reads were assembled into two sets of partially phased contigs with Hifiasm’s default parameters, as a contig in the unphased assembly may mix alleles from different parental haplotypes in a diploid or polyploid genome. The resulting haplotigs (contigs from the same haplotype) of Chr5 chromosome (identified as putative sex chromosome described below) were separated into Z or W groups using SNPs showing sex-linkage in the natural population sample of sexed individuals, based on a custom script and Longphase v1.5.1 (Lin et al. 2022). The phased contigs of W contigs for chr5 chromosome were anchored into chromosome-level assemblies using RAGTAG v2.1.0 (Alonge et al. 2022) based on the chromosome-level assembly of the *H. sajori* male.

To evaluate the quality of the assembled genome, the completeness and accuracy were assessed via short-read mapping using BWA v0.7.17 (Li 2013) and BUSCO v5.3.0 (Manni et al. 2021) using actinopterygii_odb10 gene dataset with default parameters.

### Gene Prediction and Annotation

Genes were predicted using evidence from three methods, including ab initio gene predictors, homology protein searching, and transcript alignments. First, a de novo repeat library was constructed using EDTA v2.0.1 (Ou et al. 2019) and repeats were identified using REPEATMASKER v4.1.2 (https://repeatmasker.org/RepeatMasker/). Repeat sequences excluding low-complexity components were soft masked (i.e. converted to lower case symbols). Next, BRAKER v2.1.6 (Brůna et al. 2021) was used to train the gene prediction tools GENEMARK v4.69 (Brůna et al. 2020) and AUGUSTUS 3.4.0 (Stanke et al. 2008) and generate ab initio predictions based on RNA-seq data and protein homology information of Actinopterygii from the UniProt proteomes database. In a third analysis, RNA-seq reads were assembled using TRINITY v2.11.0 (Grabherr et al. 2011), and the results were passed to PASAPIPELINE v2.4.1 (Haas et al. 2003) to generate high quality gene structures. Protein sequences of Actinopterygii from the UniProt proteomes database and RNA-seq reads were also extracted by alignment to the genomes using METAEUK v 57b63975a942fbea328d8ea39f620d6886958eca (Levy Karin et al. 2020), MINIMAP2 v2.17-r941 (Li 2018), and STRINGTIE v2.2.1 (Shumate et al. 2022). Finally, we combined the above ab initio gene predictions, protein and transcript alignments into weighted consensus gene structures using EVIDENCEMODELER v1.1.1 (Haas et al. 2008) accomplished by FUNANNOTATE v1.8.10 (https://github.com/nextgenusfs/funannotate). Protein functions were annotated using Funannotate from alignments on multiple databases including the Swiss-Prot/TrEMBL, Pfam-A, EggNOG, BUSCO, and InterProScan.

### Chromosomal Synteny and Karyotype

Both the halfbeaks studied here have 2*n* = 40 karyotypes. To identify genomic synteny among *H. sajori*, *H. intermedius*, and the distant outgroup, *O. latipes* and explore the possibility of chromosome fusions, we conducted chromosome pairwise synteny analyses with *H. sajori*, using the MCscan pipeline (implemented in JCVI python package) (Tang et al. 2008) with protein-coding sequences as input. To compare the two halfbeaks, we also used Minimap2 for pairwise whole-genome alignment, with the parameter “-cx asm10”. We numbered the chromosomes of *H. sajori* according to their assembly lengths, with the longest one named HsaChr1. The IDs of *H. intermedius* chromosomes were renamed to follow the same order as *H. sajori* chromosomes carrying homologous genes. Protein or whole-genome synteny results were visualized in the R package Circlize v 0.4.15 (Gu et al. 2014) or Ngenomesyn v1.41 (He et al. 2023).

As pericentromeric regions with rare recombination are sometimes associated with evolution of SLRs, we used the CentroMiner module in quarTeT v1.2.1 (Lin et al. 2023) to identify candidate centromeres and localize them on each chromosome, using genomic FASTA and transposable element annotation files as the input. Each chromosome yielded several candidate centromere regions, and we manually selected the most likely region based on high tandem repeat (TR) density and low gene density. To validate these locations, we used CentIER v3.0 (Xu et al. 2024) to identify centromere regions based on sequence specificity and analysis of long terminal repeat retrotransposons. The results allowed us to distinguish metacentric and acrocentric chromosomes. We then implemented TRASH v1.2 (Wlodzimierz et al. 2023) with default parameters to infer repetitive centromeric monomers from our assembly of *H. sajori*. Generally, the most abundant TR in a given genome is the candidate for centromere repeats. For the 10,277 TR monomers identified by TRASH, we retained sequences longer than 100 bp. The five most abundant monomers, termed CenHsa_1-5, were selected as centromeric repeat candidates. Screening these candidates using seqtk telo (https://github.com/lh3/seqtk) detected no potential telomeric repeats. Of these candidate centromeric monomers, CenHsa_1 and CenHsa_2 had the largest copy numbers (with slightly more CenHsa_1 than CenHsa_2 copies), making these the most likely centromeric monomers. Interestingly, we found a CENP-B box-like sequence in CenHsa_2, showing weak similarity to the human CENP-B domains. Only two candidate centromeric monomers longer than 100 bp were identified by CentIER. These two sequences are 97% identical and were similar to the CenHsa_1 sequence (identity >97%). The *H. sajori* CenHsa_1 and CenHsa_2 show low sequence identity to monomers of the medaka (*O. latipes*), threespine stickleback (*Gasterosteus aculeatus*), and Atlantic salmon (*Salmo salar*).

### Identification of SLRs

For both halfbeak species, our raw reads were quality-filtered and processed with Fastp v0.21.0 (Chen et al. 2018). The clean reads generated were mapped to the chromosome-level genome of the female *H. intermedius* and male *H. sajori* using BWA-MEM v0.7.17 (Li 2013) with default parameters. The alignment files were processed using SAMtools v1.9 (Li et al. 2009), and PCR duplications were marked using the *markdup* function in SAMtools. SNP calling was performed with BCFtools in SAMtools based on a Bayesian framework, and SNPs were stored in a VCF file. We used VCFtools v 0.1.17 (Danecek et al. 2011) to filter SNPs with parameters “–remove-indels –maf 0.05 –min-alleles 2 –max-alleles 2 –min-meanDP 5 –max-meanDP 50 –max-missing 0.6 –minQ 20 –minGQ 20 –minDP 3 –maxDP 70”. The criteria employed here were less stringent when using these SNPs for inferring sex-linked variants.

Fully sex-linked SNPs should be often heterozygous in individuals of one sex but homozygous in the other sex. We used a custom Perl script to identify sex-linked SNPs in both species. Allowing for possible missing genotypes in some individuals, for *H. sajori*, we used the criterion that, for each sex, at least 18 of our 24 individuals must be heterozygous in one sex but homozygous in the other. For *H. intermedius* (with 12 individuals of each sex in our sample), we required at least nine heterozygotes in one sex.

As male-specific variants were not identified across any extensive *H. intermedius* genome region, we conducted a kmer-based analysis, a reference-free approach, to avoid possible biases or limitations from the choice of reference genome. Kmers of 31 bp were counted for each of 12 individuals of each sex, using kmc v3.0.0 (Kokot et al. 2017). The sets of kmers used for analysis were combined and filtered following the kmersGWAS pipeline (Voichek and Weigel 2020). Using a table of the presence/absence pattern of each kmer for each individual, we selected male-specific kmers, defined as present in at least 11 of the 12 male individuals and absent in all 12 females. Raw reads containing these kmers were extracted using BBMap v39.19 (Bushnell 2014) and assembled into contigs using MEGAHIT with default parameters. The assembled contigs were mapped to the genome assembly using Minimap2 with the default parameters.

SNP densities and read coverage were calculated for each individual, first along the female *H. sajori* chr5 (Z). Then, in 50 kb windows, we computed female to male (F:M) fold difference of the values as log2 (mean female value)–log2 (mean male value). We also calculated genome-wide *F*_ST_ between males and females in 50 kb sliding windows using the VCFtools command “–fst-window-size 50000 –fst-window-step 50000 –weir-fst-pop female_individual_list –weir-fst-pop male_individual_list”.

### Linkage Disequilibrium

For the SLR identified in *H. sajori*, we computed LD across chromosome 5 using either all individuals or just males. We retained only SNPs with the criteria: “–maf 0.15 –max-missing 1” and thinned SNPs to at most one per 10 kb (−thin 10,000). Paired *r*^2^ values were then calculated with VCFtools “–geno-r2” for diploid data. We computed the second highest *r*^2^ across all pairwise SNP comparisons in 100 kb windows and displayed these values. PopLDdecay (Zhang et al. 2019) was used to compare LD decay on the autosomes, and in the PAR, Stratum1, and Stratum2 of the *H. sajori* SLR. The two natural populations were analyzed separately for each plot.

### Evolutionary Strata and Divergence Times

We extracted protein-coding sequences of SLR genes from the *H. sajori* ChrZ haplotype, and all annotated protein-coding sequences from the ChrW one. The inferred Z and W protein sequences were compared using Diamond v2.0.15.153 (Buchfink et al. 2021) with a query coverage cutoff of >0.6. Gene pairs identified as reciprocal best hits (RBHs) were retained for sequence divergence estimation (any many-to-one relationships were also removed). Then the *K*a and *K*s values between Z-W homolog pairs were calculated using KaKs_calculator 3.0 (Zhang 2022) with the Nei and Gojobori (1986) correction for saturation. Homologous gene pairs from the homologous chromosomes HsaChr5 and HinCh5 of *H. sajori* and *H. intermedius* were compared using the same methods to estimate the inter-species *K*s values and their mean. SNP density and sequence divergence (*K*a and *K*s) along the Z chromosome suggested two evolutionary strata in the non-PAR region of the *H. sajori* sex chromosome. We used the R package “changepoint” v 2.2.4 (Killick and Eckley 2014) to detect the boundary between strata based on SNP density differences between the sexes, with parameters such that one changepoint was allowed under the mean and variance methods.

To further investigate the evolutionary history of the *H. sajori* sex chromosomes, we examined the phylogenetic relationship of single-copy genes shared between *H. intermedius* and the Z/W region of *H. sajori*, including their orthologs in six other halfbeaks, as more distant outgroups (*R. georgii*, *H. far*, *H. archipelagicus*, *H. gernaerti*, *H. limbatus*, and *H. paucirastris*). We ran OrthoFinder on these nine data sets to identify orthogroups and extract coding sequences of single-copy orthogroups shared by these datasets. We then aligned the sequences of these single-copy orthogroups using MACSE and construct gene trees using IQ-TREE. Gene trees were rooted on *R. georgii*, the most distant relative of the two focal species. We then used the ete3 package (Huerta-Cepas et al. 2016) implemented in Python to classify the gene trees into three possible topologies: Z and W of *H. sajori* are sisters, the *H. intermedius* is sister to the *H. sajori* Z (loss of the W sequence in *H. intermedius*), and *H. intermedius* is sister to W (loss of Z).

To estimate the divergence time in years between Z- and W-linked genes, using the *K*s values, we selected six teleost species (including *O. latipes*, *D. rerio*, *C. semilaevis*, *T. rubripes*, *G. aculeatus*, and *O. niloticus*) to construct a phylogenetic tree including *H. sajori* and *H. intermedius*. The genome sequences and annotation files were downloaded from Ensemble. Orthologs were clustered using OrthoFinder v.2.3.4 (Emms and Kelly 2019). Four-fold degenerate sites in coding sequences were then extracted from single-copy genes and concatenated to estimate the phylogeny. We used mcmctree program in the PAML package v4.10 (Yang 2007) to estimate divergence times among these species, with 5,000,000 replicates and 1,000 burn in rounds. The estimated divergence times of *O. niloticus* and *O. latipes* (81–96 Mya), and *D. rerio* and *C. semilaevis*: (180–250 Mya), from the TIMETREE website (http://www.timetree.org), were used as calibration times. We also used the *K*s value for the divergence between the two halfbeaks; the estimated value of 0.048 yields a mutation rate of 1.14e−8/site/year. We assumed that the Z and W sequences diverge at the same rate as the inter-species to obtain the Z-W allele divergence times in years.

### Analysis of Sex Chromosome Degeneration

We first mapped short-read data from 24 males and 24 females of *H. sajori* to the male (ZZ) genome. Aligned bam files were sorted using SAMtools v1.18 and duplicates were marked using the MarkDuplicates module of Picard v 2.27 (https://broadinstitute.github.io/picard/). We further called SNPs and indels smaller than 30 bp in the HsaChr5 SLR using the HaplotypeCaller module of GATK4 v.4.2.6.1 (Van der Auwera et al. 2013). The vcf files generated were filtered using VCFtools with parameters “–maf 0.05 –min-alleles 2 –max-alleles 2 –min-meanDP 5 –max-meanDP 50 –max-missing 0.6 –minQ 20 –minGQ 20 –minDP 3 –maxDP 70”. Female-specific SNPs or indels (heterozygous in more than 18 females and homozygous in all males) were identified using a custom Perl script (https://github.com/lyl8086/find_sex_loci) based on variants generated by GATK4. For deletions or insertions of length >30 bp (termed “large”), we mapped PacBio HiFi reads of female *H. sajori* to the male genome using pbmm2 v1.12.0 (https://github.com/PacificBiosciences/pbmm2) with parameters “–sort –preset CCS”. Aligned reads were phased and tagged as either “HP:i:1” or “HP:i:2” using Longphase, based on the presence of the female-specific SNPs identified, with HP:i:2 being the W haplotype. We extracted aligned HiFi reads that were tagged as “HP:i:2” (long W reads) into a single bam file, which was used to call SVs using two pipelines, CuteSV v2.1.1 (Jiang et al. 2020) with parameters “–max_cluster_bias_INS 1000 –diff_ratio_merging_INS 0.9 –max_cluster_bias_DEL 1000 –diff_ratio_merging_DEL 0.5 –max_cluster_bias_TRA 1000 –diff_ratio_filtering_TRA 0.5 –min_support 3 –max_size 1000000 –genotype” and pbsv v2.9.0 (https://github.com/PacificBiosciences/pbsv) with “discover –ccs” and “call –ccs”. We combined SNPs and small indels from short reads with the large SVs ascertained in this way. The final VCF files were then annotated using SnpEFF v 5.1d (Cingolani et al. 2012) for analyses to infer how these variants are likely to affect the functions of genes in the W-linked region.

Female-specific variants identified as outlined above were all based on evidence of sex-linkage in population samples. We also evaluated the degeneration of W-linked regions using our assemblies of Z and W chromosomes (one W and one Z haplotype were used) as follows. We first retrieved the inferred protein sequences of genes within the Z-linked regions. These were used as queries of the W assembly using Miniprot v0.12-r237 (Li 2023) with parameters “-L 10 -J20 –outs = 0.8 –outc = 0.5 –gff -u”. Genes with query coverage lower than 0.5 or alignment identity less than 0.6 were treated as missing from the W. Genes whose sequences included frameshifts, in-frame stop codons, and splice donor or acceptor variants were marked and combined with the missing genes as reflecting W-linked loss of function.

### Dosage Compensation

Total RNA was extracted from fresh liver and muscle tissue of adult *H. sajori*, with two biological replicates for each tissue of each sex, resulting in eight sequencing libraries being constructed and sequenced on Illumina NovaSeq 6000 platform with PE150 chemistry. The raw reads were filtered with Fastp to remove adaptor sequences and end bases with low quality. The clean RNA reads were aligned to a reference genome using Hisats2 v 2.2.1 (Kim et al. 2019) and the transcript regions annotated. The BAM files were sorted and indexed with SAMtools. Together with the VCF file of sex-linked SNPs (to identify Z- or W-linked reads), W or Z-specific read counts for females were calculated using sorted bam files with ASEReadCounter with default parameters from GATK v4.1. Z or W-linked reads were assigned based on pre-identified female-specific SNP information. Genes’ expression levels were normalized by the transcripts per million (TPM) method (Li and Dewey 2011). To compare the distributions of Z-linked expression values in females (with a single Z) with those from the ZZ males’ two Z-linked alleles and test for dosage compensation in females of genes in different categories of W-linked expression, we divided the sex-linked genes into four categories according to their W:Z expression ratios in females and used Hartigan’s diptest to test for unimodality or multimodality using the R Package “diptest” (https://github.com/mmaechler/diptest). To detect groups of genes that are dosage compensated or uncompensated, we applied Gaussian mixed modeling (GMM) to the ratio of Z-linked expression in females to ZZ males expression using the R package *mclust* (Scrucca et al. 2016). Nonunimodality was detected only for genes with extremely low W-linked allele expression, or no detectable expression. The GMM analysis was therefore applied only for these genes.

## Supplementary Material

msaf151_Supplementary_Data

## Data Availability

The sequencing data that support the findings of this study are openly available in the NCBI Sequence Read Archive under BioProject Accession No. PRJNA1171142 and PRJNA1170067. Custom scripts are available on GitHub at https://github.com/xtf2020/HalfbeaksSCE.
