# Supplementary material for: Extensive Recombination Suppression and Genetic Degeneration of a Young ZW Sex Chromosome System in Halfbeak Fish
Source: Mol Biol Evol. 2025 Jun 24;42(7):msaf151. doi: 10.1093/molbev/msaf151 (PMC12258149; doi:10.1093/molbev/msaf151)
Supplement: msaf151_Supplementary_Data [file msaf151_supplementary_data.pdf]

Supplementary Materials for

Extensive recombination suppression and genetic degeneration of a young ZW sex chromosome system in halfbeak fish

Teng-Fei Xing [1,2]†, Yu-Long Li[1,2]†, Hao Yang[1,2,3], Deborah Charlesworth[4] *, Jin-Xian Liu[1,2, *]

[1] Key Laboratory of Marine Ecology and Environmental Sciences, Institute of Oceanology, Chinese Academy of Sciences, Qingdao 266071, China

[2] Laboratory for Marine Ecology and Environmental Science, Qingdao National Laboratory for Marine Science and Technology, Qingdao 266237, China

[3] University of Chinese Academy of Sciences, Beijing 100049, China

[4] Institute of Ecology and Evolution, School of Biological Sciences, University of Edinburgh, Charlotte Auerbach Road Edinburgh EH9 3FL, United Kingdom

† These authors contributed equally to this work

*Corresponding author

Email: Deborah.Charlesworth@ed.ac.uk; jinxianliu@gmail.com

The PDF file includes:

Figs. S1 to S30
Tables S1 to S16

Figs. S1 to S30

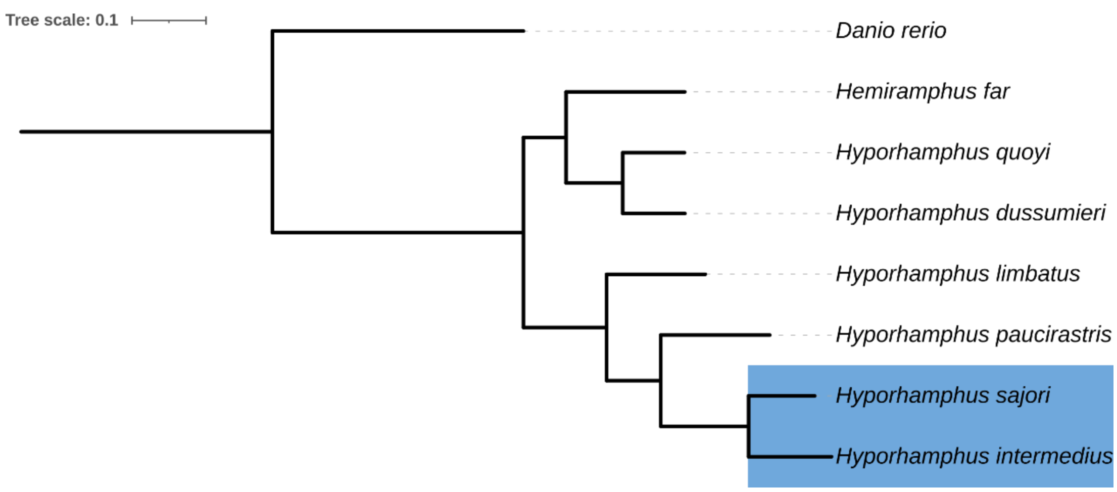

Fig. S1 ML tree of *H. sajori* and its close relatives using cds sequences of 13 mitochondrial protein-coding genes.

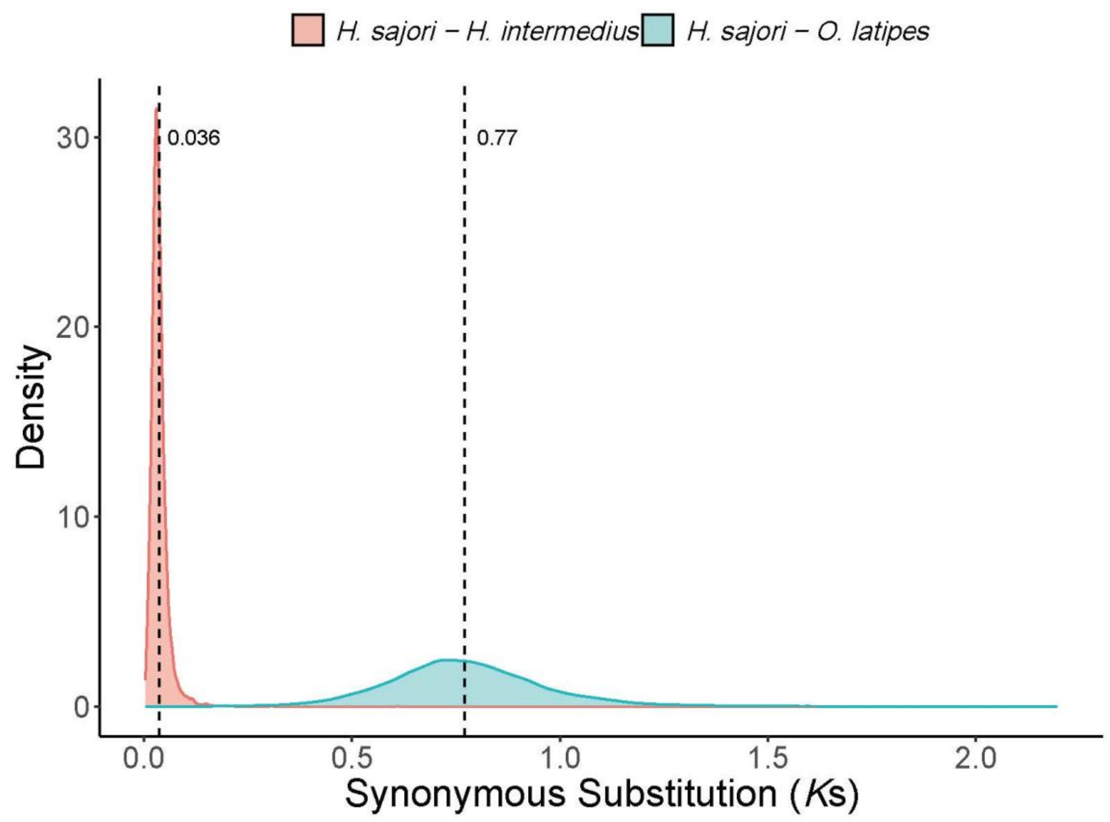

Fig. S2 Distribution of synonymous site divergence (*K*s) for *H. intermedius vs*. *H. sajori* and *H. sajori vs.* medaka, calculated using 7,240 genes that have single copies in all three species. Mean Ks values are indicated by vertical dotted lines.

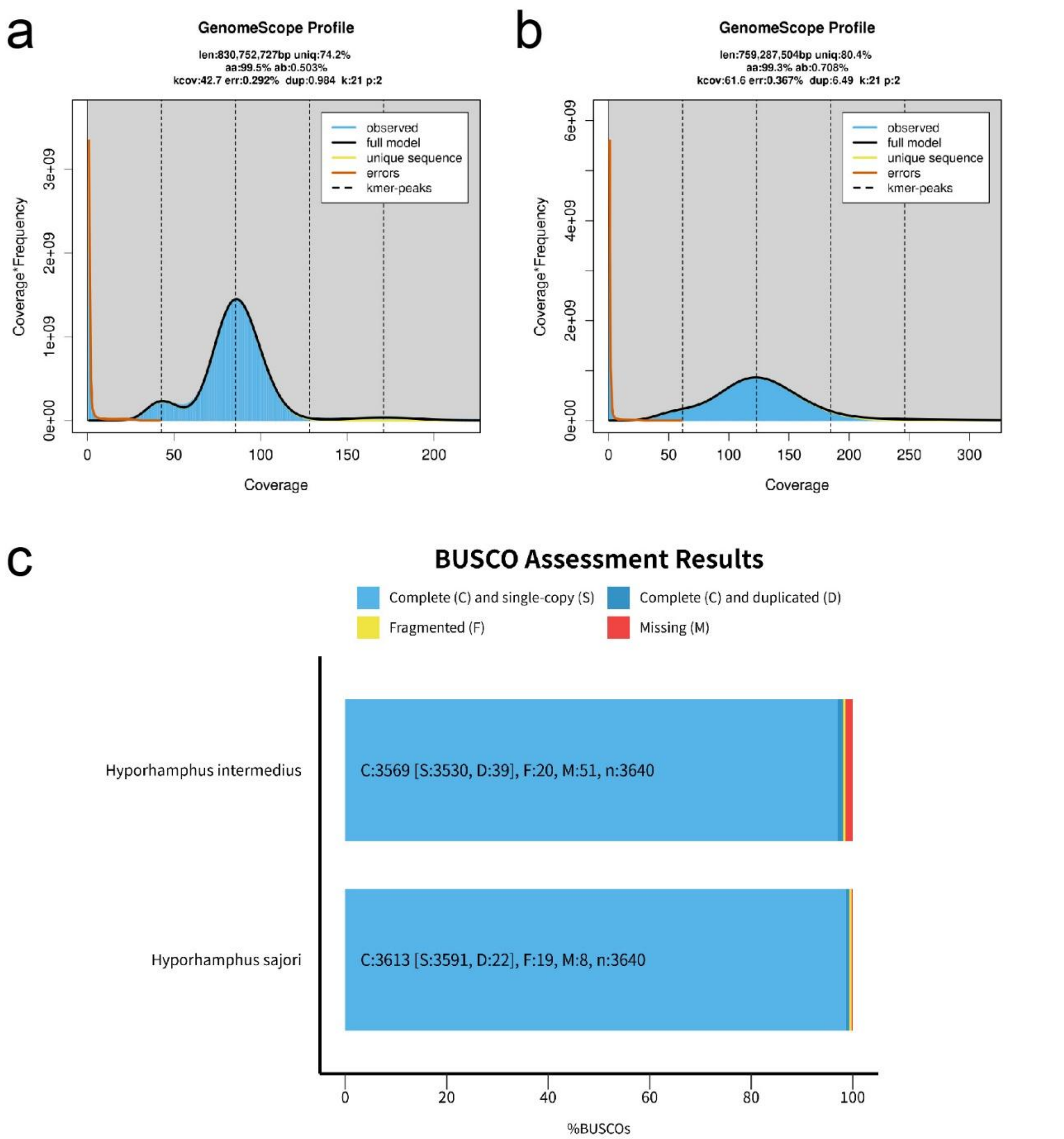

Fig. S3 The k-mer distribution of the short reads data using GenomeScope (Ranallo-Benavidez et al. 2020) based on a k value of 21 for (a) female *H. intermedius* and (b) male *H. sajori*. (c) Busco results for the assemblies of the two halfbeak species.

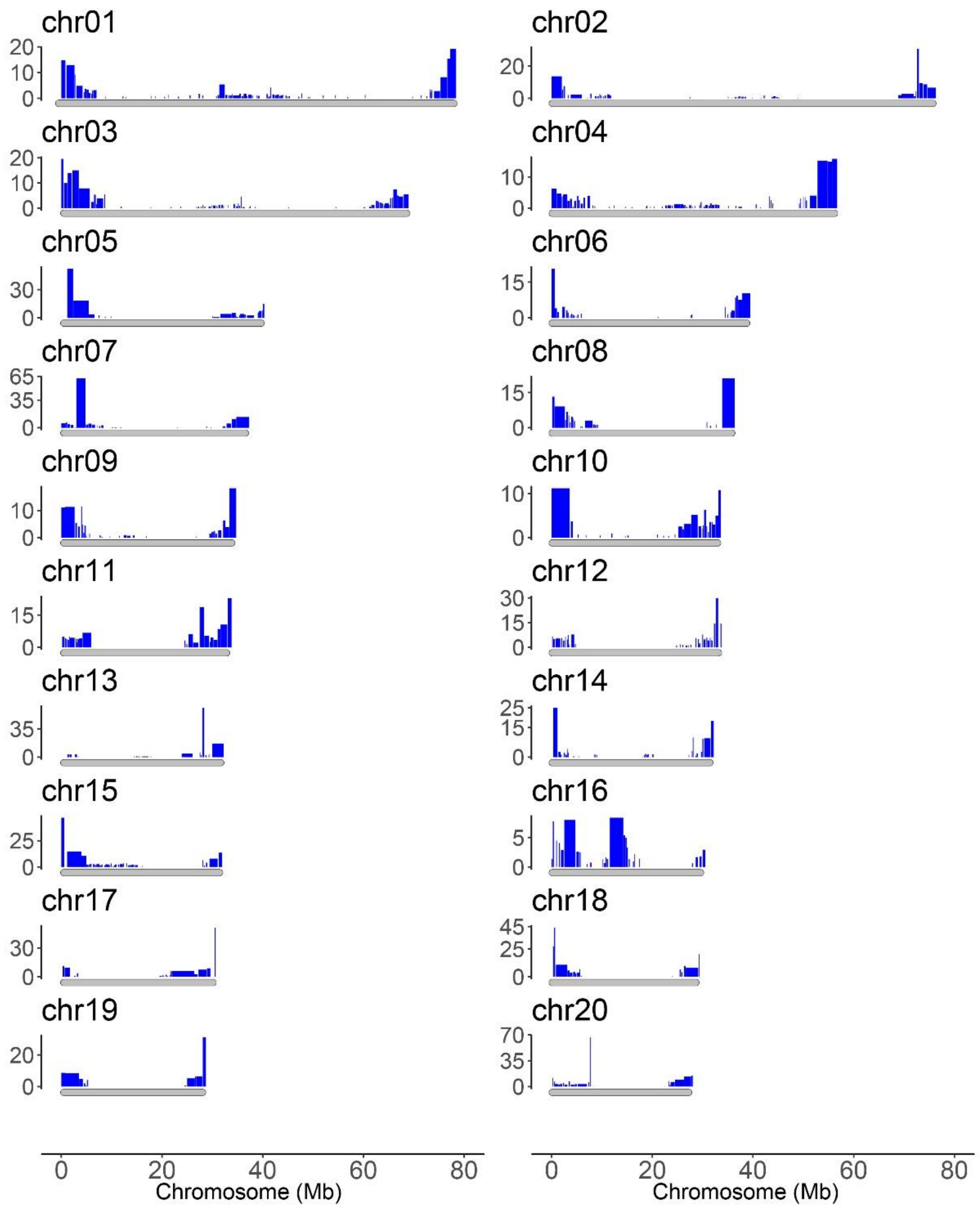

Fig. S4 Results of QuarTeT analysis to infer centromere positions in *H. sajori*. The analysis yielded several candidate centromere regions, and the figure shows the tandem repeat contents (%) of these candidate regions, in bar plots for each chromosome. The HsaChr5 centromere is probably located at the left of this chromosome's assembly.

a

IER_1

TTACTTTTTCTGTCCCAGCGTCCTTAATTCTTCATGTTGATGGCATAAAACAGTGTTAGAGA
GCAGTTTTAGGTGTTTTTGATTGAATTCCATAAGTTTTGCAAAAAGCCCCAAAATCAAGTTT
TCTGCCCGAGAAACGTCTAAATACAGCGTTTCCAGGCGCTTAAAGTGCATCCTTAGGTGTT
TTTGCTGGAAAGCTGTGTAAC

IER_2

GCAAAACTTATGGAATTCAATAAAAAACACCTAAAACTGCTCTCTAACACTGTTTTATGCC
ATCAACATGAAGAATTAAGGATGCTGGGACAGAAAATGCATGTTACACAGCTTTCCACCAA
AACCACCTAACGTTCCACTCTAAGCGTCTGAAGCCGCTGTATGTGGACGTTTCTCGGCCAG
AAAACATGATTTTGGGGCTTTTT

b

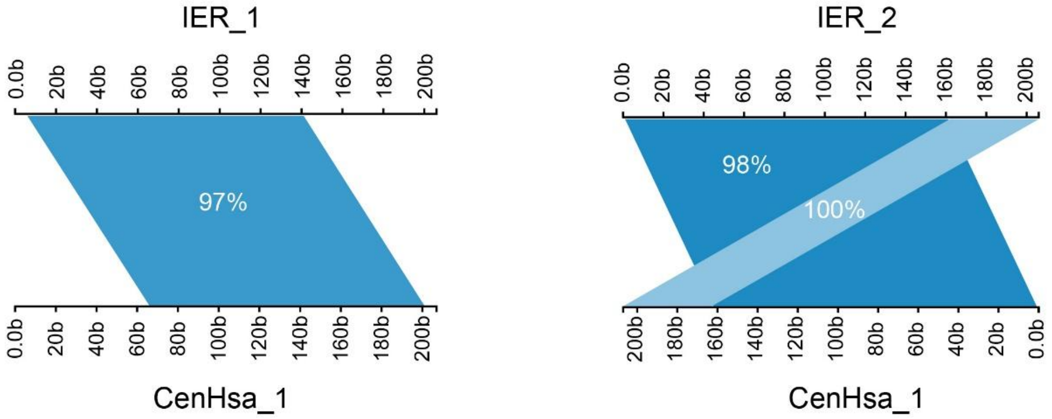

Fig. S5 Two monomers with lengths larger than 100 bp were identified by CentIER (termed IER_1 and IER_2). (a) Sequences of IER_1 and IER_2. (b) Blastn results between IER_1/2 and CenHsa_1. Sequence identity is annotated in the figure. Blast results were visualised by Kablammo (Wintersinger and Wasmuth 2015).

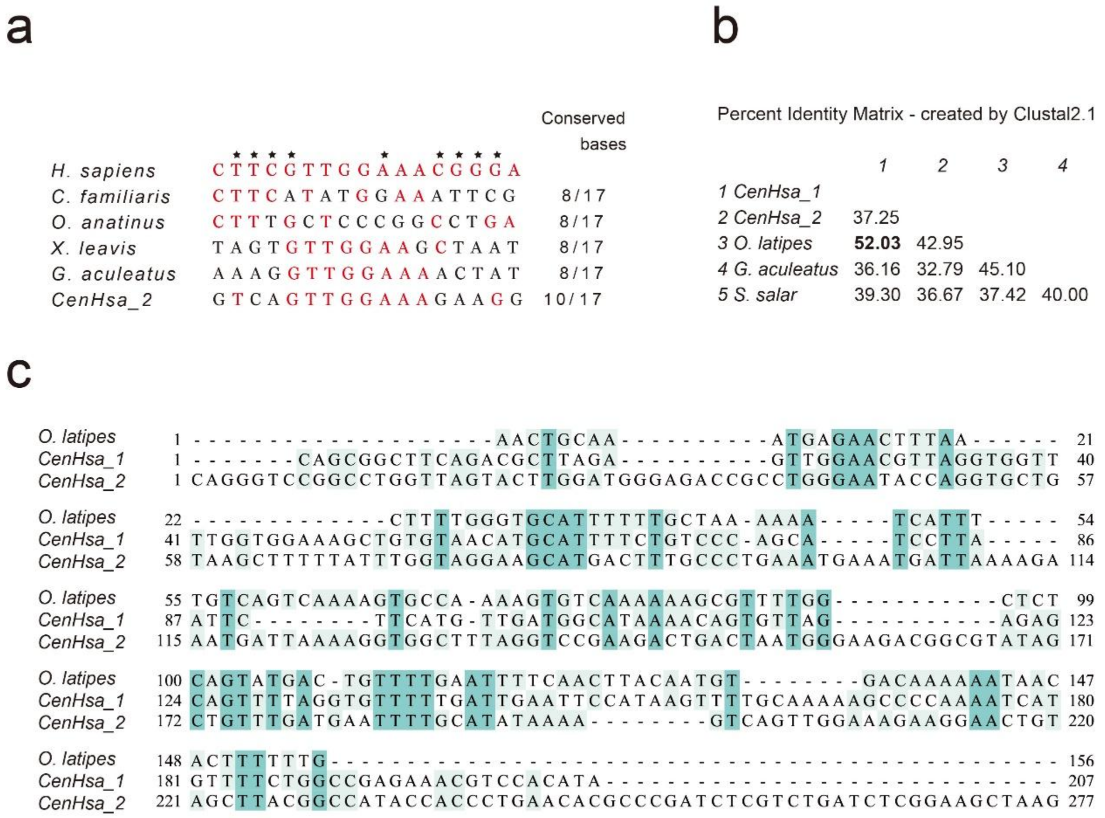

Fig. S6 The putative centromeric monomers CenHsa_1 and CenHsa_2. (a) The putative CENP-B box in CenHsa_2 shows sequence similarity to the CENP-B box in human (*Homo sapiens*), dog (*Canis familiaris*), platypus (*Ornithorhynchus anatinus*), and the African clawed frog (*Xenopus laevis*), threespine stickleback (*Gasterosteus aculeatus*). Identical nucleotides to the human CENP-B box are in red, and asterisks denote the evolutionary conserved domains in humans. This figure was created following (Cech and Peichel 2015). (b) Percent identity matrix among CenHsa_1 and CenHsa_2 of *H. sajori*, centromeric monomers of medaka (*Oryzias latipes*) (Melters et al. 2013), threespine stickleback (*G. aculeatus*) (Cech and Peichel 2015) and Atlantic salmon (*Salmo salar*) (Viñas et al. 2004; Melters et al. 2013). (c) Sequence alignment of centromeric monomer of medaka and the candidate centromeric monomers in *H. sajori* (CenHsa_1 and CenHsa_2).

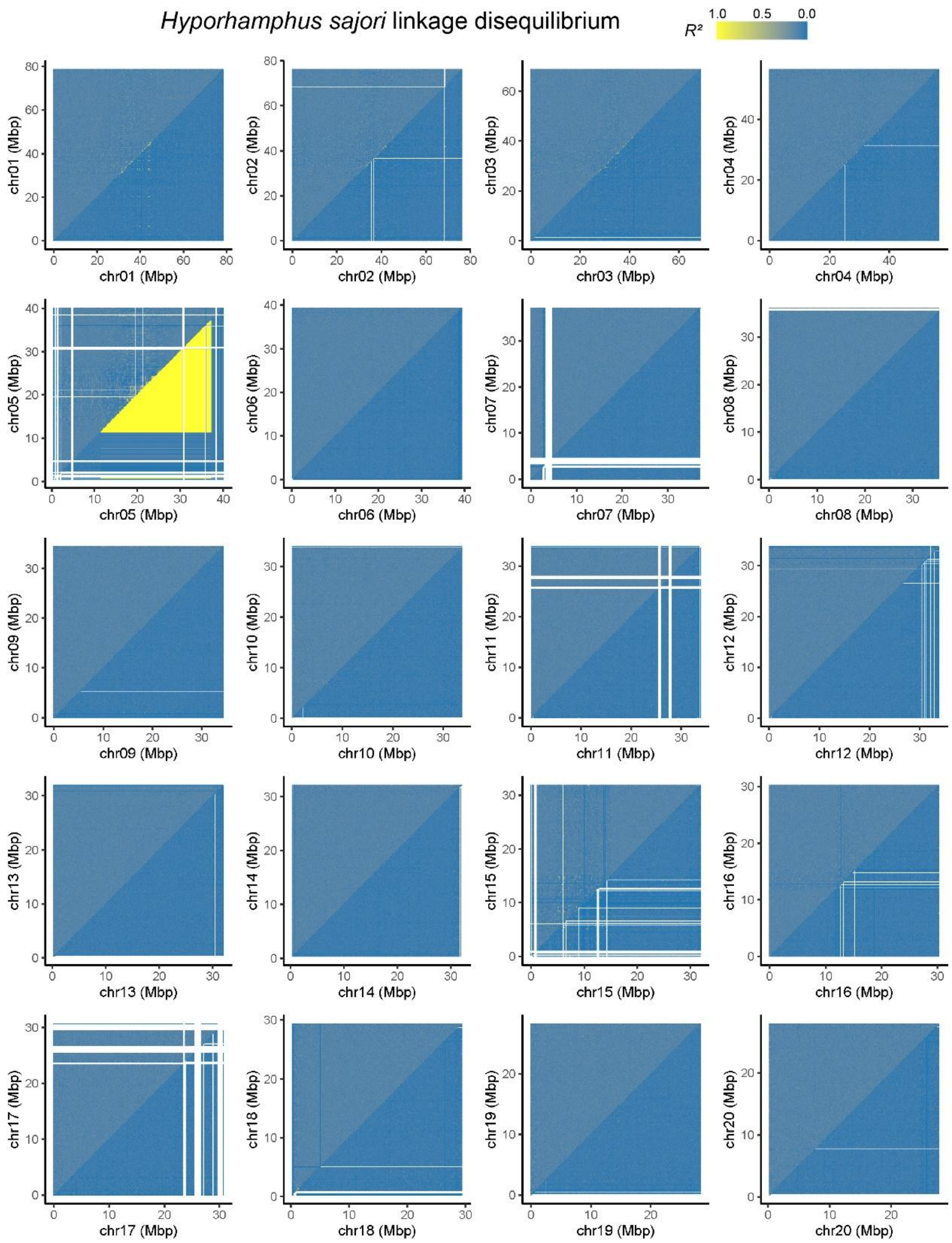

Fig. S7 Linkage disequilibrium (LD) plots for all *H. sajori* chromosomes. Cells below the diagonal show results for all *H. sajori* individuals, including both sexes; above the diagonal, only male individuals were analysed. The colors represent the second highest R^2 values in 50-kb windows, in order to indicate regions of the chromosome in which LD values differ.

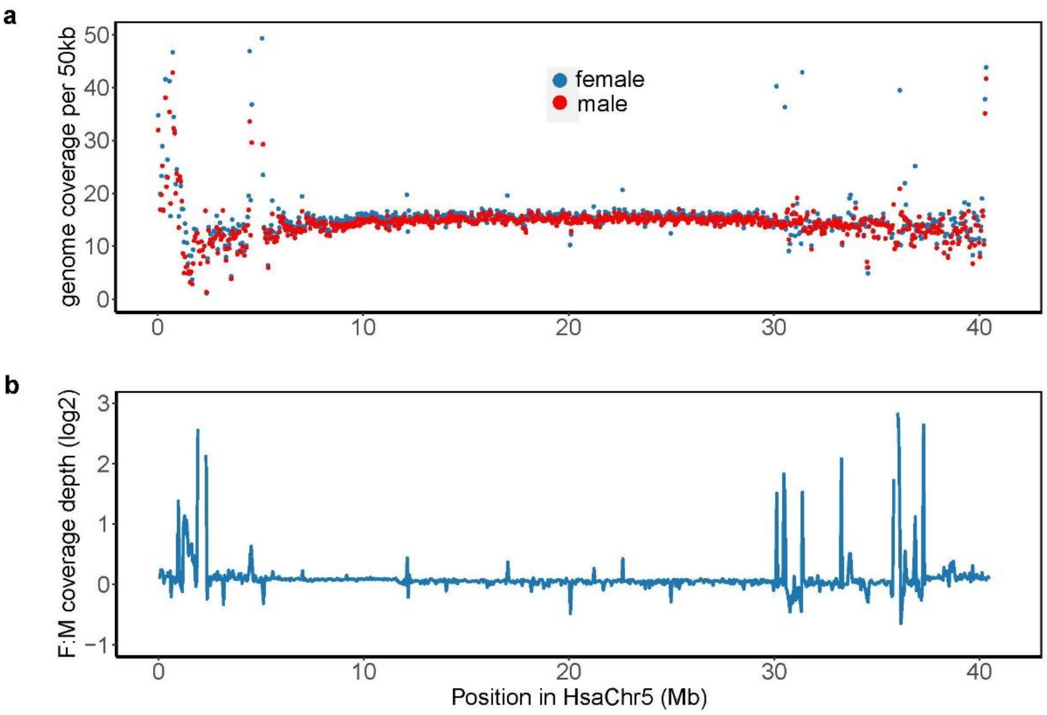

Fig. S8 Genomic depth of coverage values for females and males (a), as well as the Female:Male coverage depth ratio (using a log2 transformation) (b), both calculated in 50 kb non-overlapping windows of the HsaChr5 assembly.

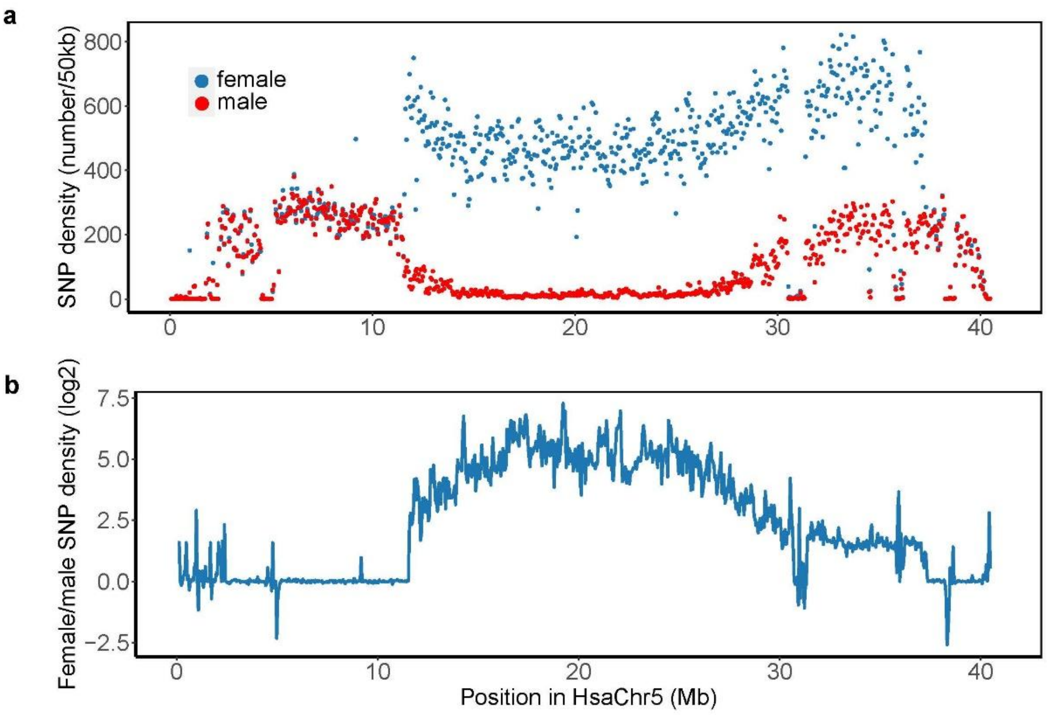

Fig. S9 SNP numbers in our natural population sample of females and males (a), as well as the Female: Male SNP density ratios (using a log2 transformation) (b), calculated in 50 kb non-overlapping windows on HsaChr5.

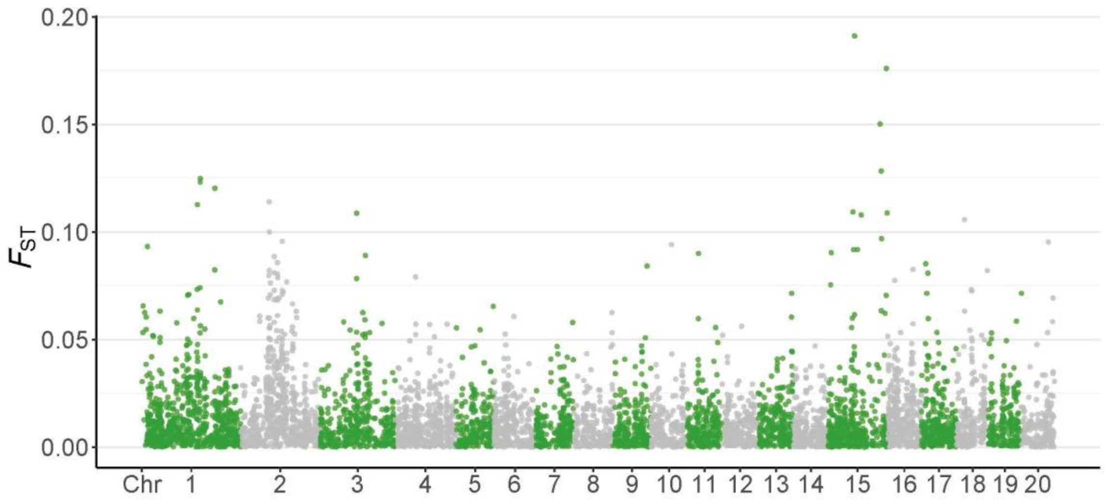

Fig. S10 Mean F_{ST} values between females and males of *H. intermedius* in 50-kb windows across all 20 chromosomes.

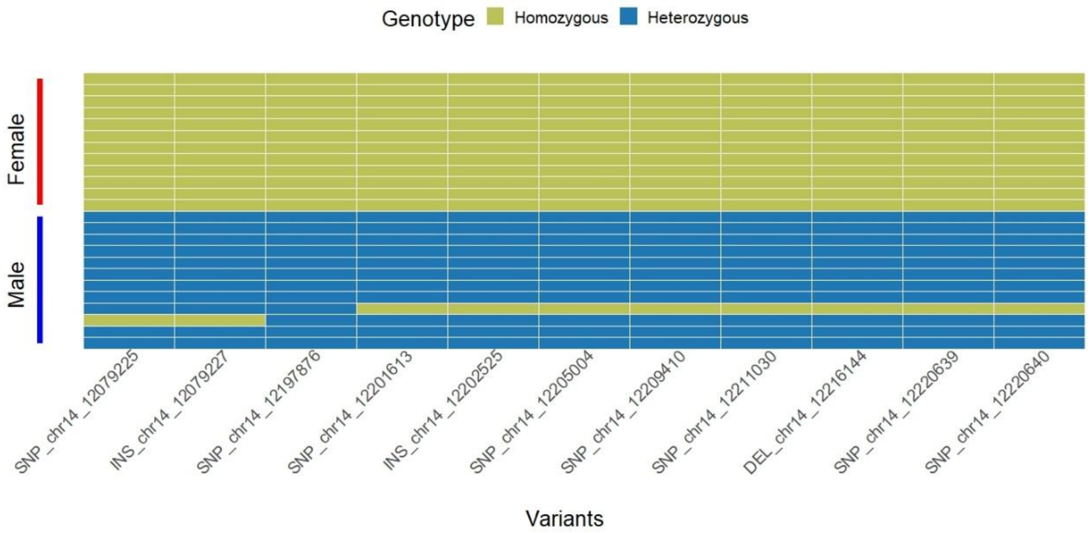

Fig. S11 Genotypes of the 11 most strongly sex-associated variants in chromosome 14 of *H. intermedius*. Each row corresponds to one female or male individual, and each column to a variant site, with their locations shown on the x axis. These sites were homozygous in all females but heterozygous in most males.

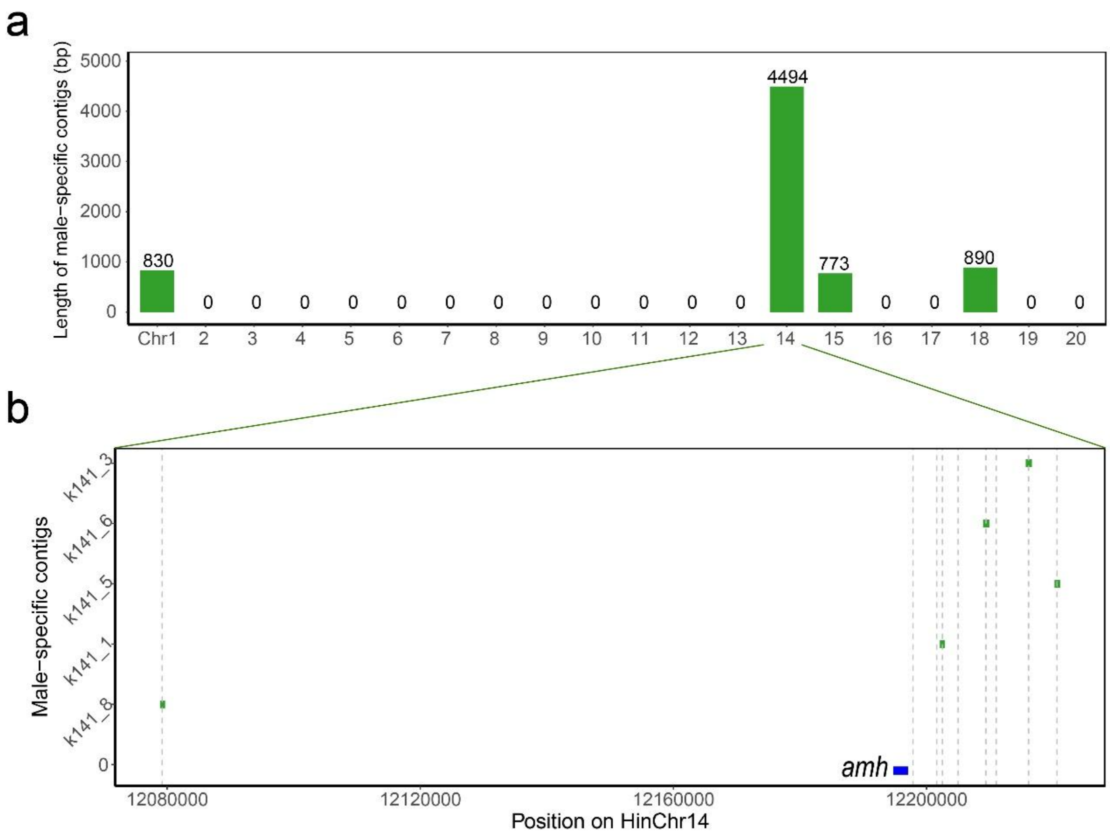

Fig. S12 Kmer-based approach of *H. intermedius*. (a) The plot shows the aligned length of 9 contigs assembled from reads containing male-specific kmers, with the cumulative aligned length (bp) showing on the top of bar for each chromosome of *H. intermedius* genome. (b) Mapping positions of the five male-specific contigs on HinChr14. Green rectangles infer the aligned range of each assembled contigs, with their ID showing on the X axis. Gray dashed lines show the positions of 11 most strongly sex-associated variants.

```
Hin/1-495     1 MQWPLVLVVECLFLCASEQSVFEKRQCVFQVTNPKNHRYAAAGNVSGSVQLCEKTRCCAGIY  62
Hsa_W/1-495   1 MQWPLVLVVECLFLCASEQSVFEKRQCVFQVTHPKNHRYAAAGNVSGSVQLCEKTRCCAGIY  62
Hsa_Z/1-495   1 MQWPLGLVVECLFLCASEQSVFEKRQCVFQVTHPKNHRYAAAGNVSGSVQLCEKTRCCAGIY  62

Hin/1-495    63 QIINGQPKIDTLACDMMETFCPDATCKAHQSLGNRFLRCVCNTDLCNNNITWAPEAEHPSHT 124
Hsa_W/1-495  63 QIKNGQPKVDTLACDVMETFCPDATCKAHQSLGNRFLRCVCNTDLCNNNITWAPEAEQPSHT 124
Hsa_Z/1-495  63 QIKNGQPKVDTLACDVMETFCPDATCKAHQSLGNRFLRCVCNTDLCNNNITWAPEAEQPSHT 124

Hin/1-495   125 HSHSAVGPLKMASVVIPSLCFVLIAAIWRCFCKEKKKELLSSRYDYSVQPAPSCQAKTSENY 186
Hsa_W/1-495 125 HSHSAVGPLKMASVVIPFLCFVLIAAIWRRFCKEKKKELLSSRHDYSVQPAPSCQAKTSENY 186
Hsa_Z/1-495 125 HSHSAVGPLKMASVVIPFLCFVVIVAIWRRFCKEKKKELLSSRQDYSVQPAPSCQAKTSENY 186

Hin/1-495   187 ITGTELQRVVGQGRFATVFQGKYKGSAVVVKMYHTGWSHIFTTEKEIYELPLMKHNGIAQFL 248
Hsa_W/1-495 187 ITGTELQRVVGQGRFATVFQGKYKGSAVVVKMYHAGWSHIFTTEKEIYELPLMKHNGIAQFL 248
Hsa_Z/1-495 187 ITGTELQRVVGQGRFATVFQGKYKGSAVVVKMYHAGWSHIFTTEKEIYELPLMKHNGIAQFL 248

Hin/1-495   249 GTGWKPDYDSWFIVLQHAKYGSLHSFLCKHTTSWMQSLKLCHSLSQGLSYLHSDIRSHDVHK 310
Hsa_W/1-495 249 GTGWKPDDDSWFIVLQHAKYGSLHSFLCKHTTSWMQSLKLCRSLSQGLSYLHSDIRSHDVHK 310
Hsa_Z/1-495 249 GTGWKPDDDSWFIVLQHAKYGSLHSFLCKHTTSWMQSLKLCRSLSQGLSYLHSDIRSHDVHK 310

Hin/1-495   311 PPVAHRDLSSSNVLVKGDGTCALCDFGCSTILHLGSGHHLWQHHDKNMKGHAQFGTLHYMSP 372
Hsa_W/1-495 311 PAVAHRDLSSSNVLVKGDGTCALCDFGCSTILHLGSGHHLWQHHDKNMKGHAQFGTLHYMSP 372
Hsa_Z/1-495 311 PAVAHRDLSSSNVLVKGDGTCALCDFGCSTILHLGSGHHLWQHHDKNMKGHAQFGTLHYMSP 372

Hin/1-495   373 EILEGSTNLNSSLYLMHGDIYSLGLVLWEIWMRCTDLFEGGIVPQHLLPYELELEANVTLER 434
Hsa_W/1-495 373 EILEGSTNLNSSLYLMHGDIYSLGLVLWEIWMRCTDLSEGGIVPQHLLPYELELEANVTLER 434
Hsa_Z/1-495 373 EILEGSTNLNSSLYLMHGDIYSLGLVLWEIWMRCTDLFEGGIVPQHLLPYELELEANVTLER 434

Hin/1-495   435 LVLYVSEMDKRPSIPESWEHLSQGSSMKELLTDCWDRDADARLTAACVVNRLVSLQASLSV  495
Hsa_W/1-495 435 LVLYVSEMDKRPSIPESWEHLSQGSSMKELLTDCWDRDADARLTAACVVNRLVSLQASLSV  495
Hsa_Z/1-495 435 LVLYVSEMDKRPSIPESWEHLSQGSSMKELLTDCWDRDADARLTAACVVNRLVSLQASLSV  495
```

Fig. S13 Comparison of amino acid sequences of *Amhr2* in the putative Z and W alleles of *H. sajori,* and the ortholog in the close outgroup species, *H. intermedius*. Derived amino acid changes were inferred in the *H. sajori* Z or W, based on comparing the state in the *H. intermedius* chromosome 5 sequence with the *H. sajori* W or Z sequences, and are marked by red boxes. The *H. sajori* Z includes 3 such changes, its W has one change, and both its Z and W differ from the *H. intermedius* sequence by 11 shared changes, suggesting that the Z and W started diverging long after the species split. There was one position in which all these three sequences are different.

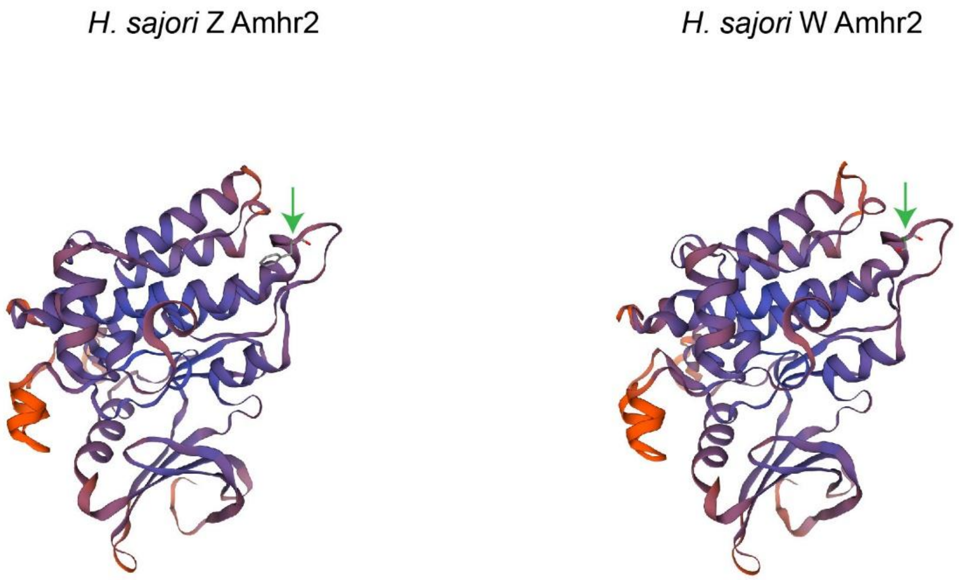

Fig. S14 The 3D structure of *Amhr2* gene for the copy on *H. sajori* Z and W, respectively. The predicted protein domain encompasses the amino acids from 184-493, within which only one amino acid change between Z-W is located (green arrow). The 3D model was predicted by Swiss-Model homology-modelling server (Waterhouse et al. 2018). The crystal structure of the kinase domain of human BMPR2 (PDB ID; 3g2f.1.A) was used as the template for homology modelling.

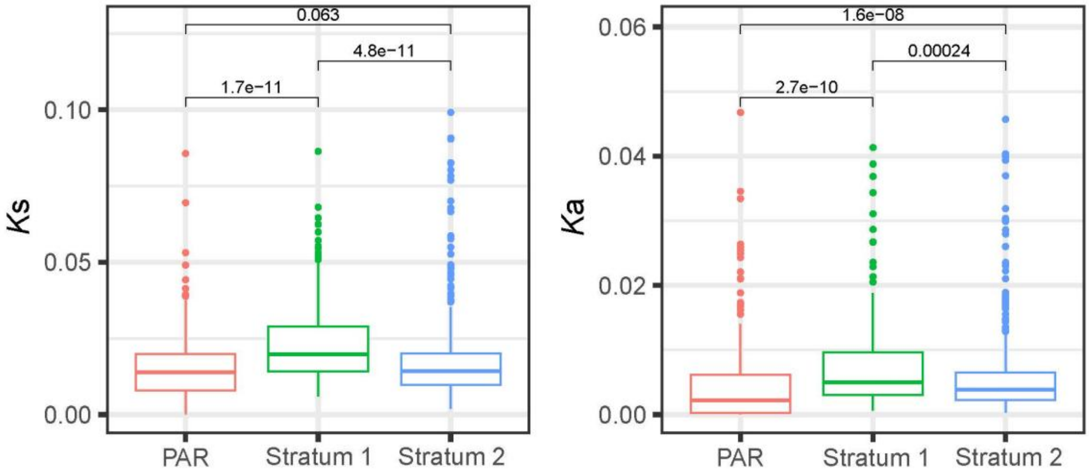

Fig. S15 The two evolutionary strata inferred in chromosome 5 of *H. sajori*. The strata differ in both synonymous site (*K*s) (left) and nonsynonymous site divergence values (*K*a) (right, note the smaller values of this scale on the y axis), and Stratum 1 is older than Stratum 2. The horizontal lines within each box indicate the median values. The top and bottom of the boxes indicate the 25th and 75th percentiles, respectively, and the coloured whiskers extend 1.5 times the interquartile ranges. The *P* values for *Mann–Whitney U* tests of significance are shown.

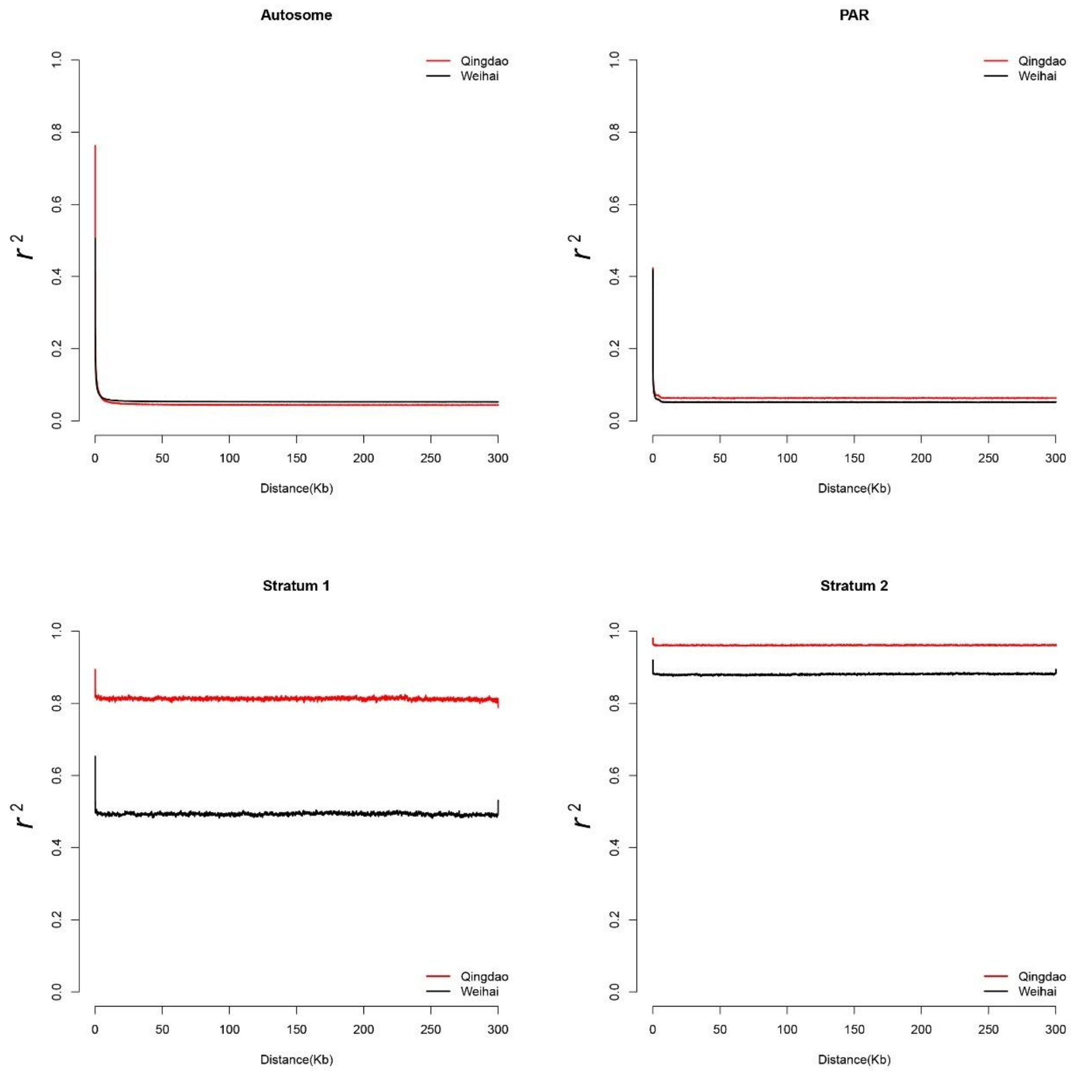

Fig. S16 Linkage disequilibrium (LD, estimated as r^2 values between pairs of SNPs whose distances apart are indicated on the x-axis). LD decay patterns are shown for autosomes, the sex-chromosome PAR, Stratum1, and Stratum2 of *H. sajori*, showing the high LD values across the sex-linked regions only, in two natural populations were analyzed separately (the red and black symbols).

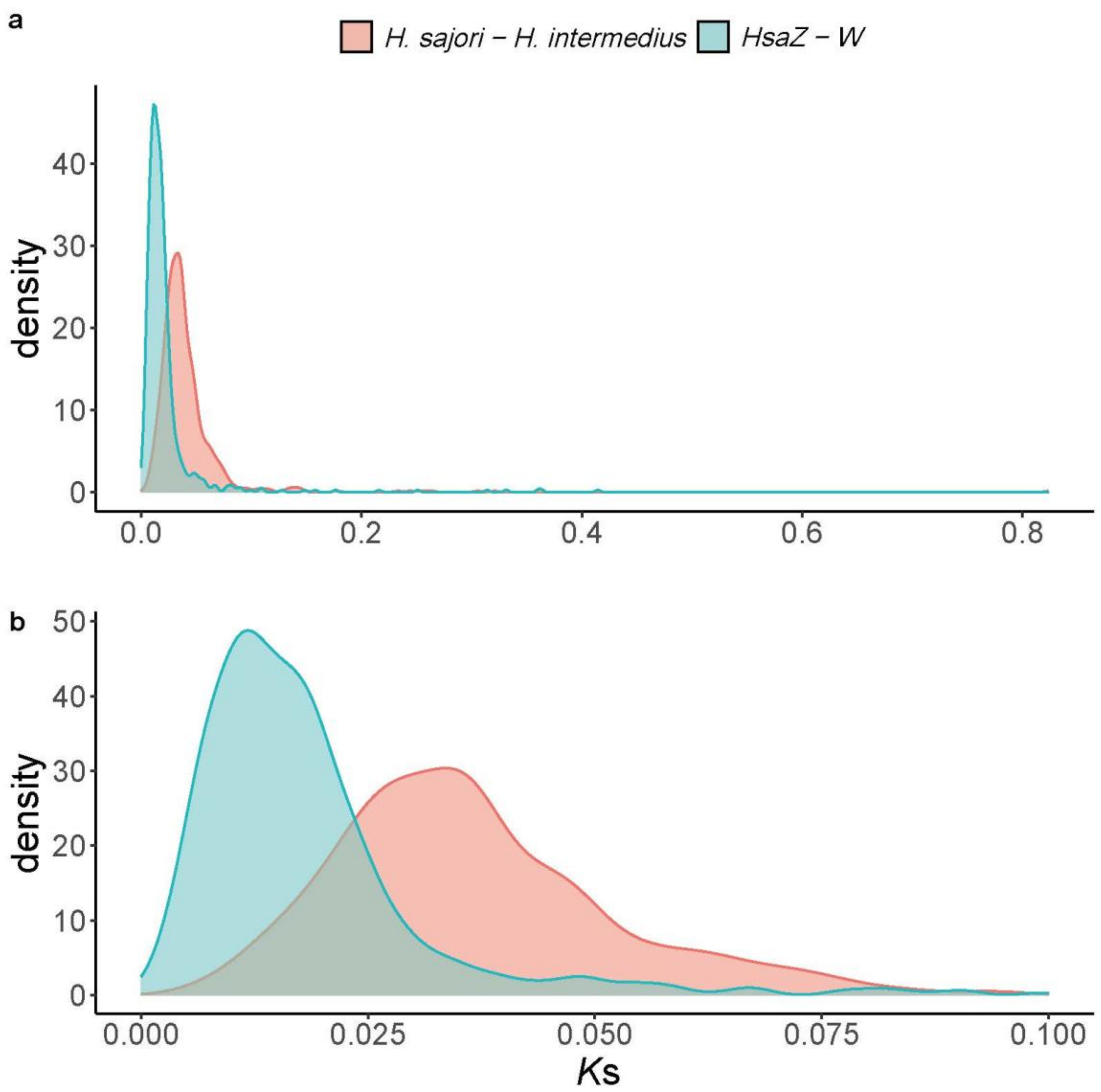

Fig. S17 Distribution of synonymous site divergence (*K*s) for gene pairs on the homologous chromosomes HinChr5 and HsaChr5 and of *H. intermedius vs*. *H. sajori* (red), and for homologous gene pairs on the *H. sajori* ChrZ and ChrW (green). The same gene set was used while calculating both sets of *K*s values. (a) shows all homologous gene pairs and (b) shows the distributions near the median values, after removing the few *K*s values larger than 0.1.

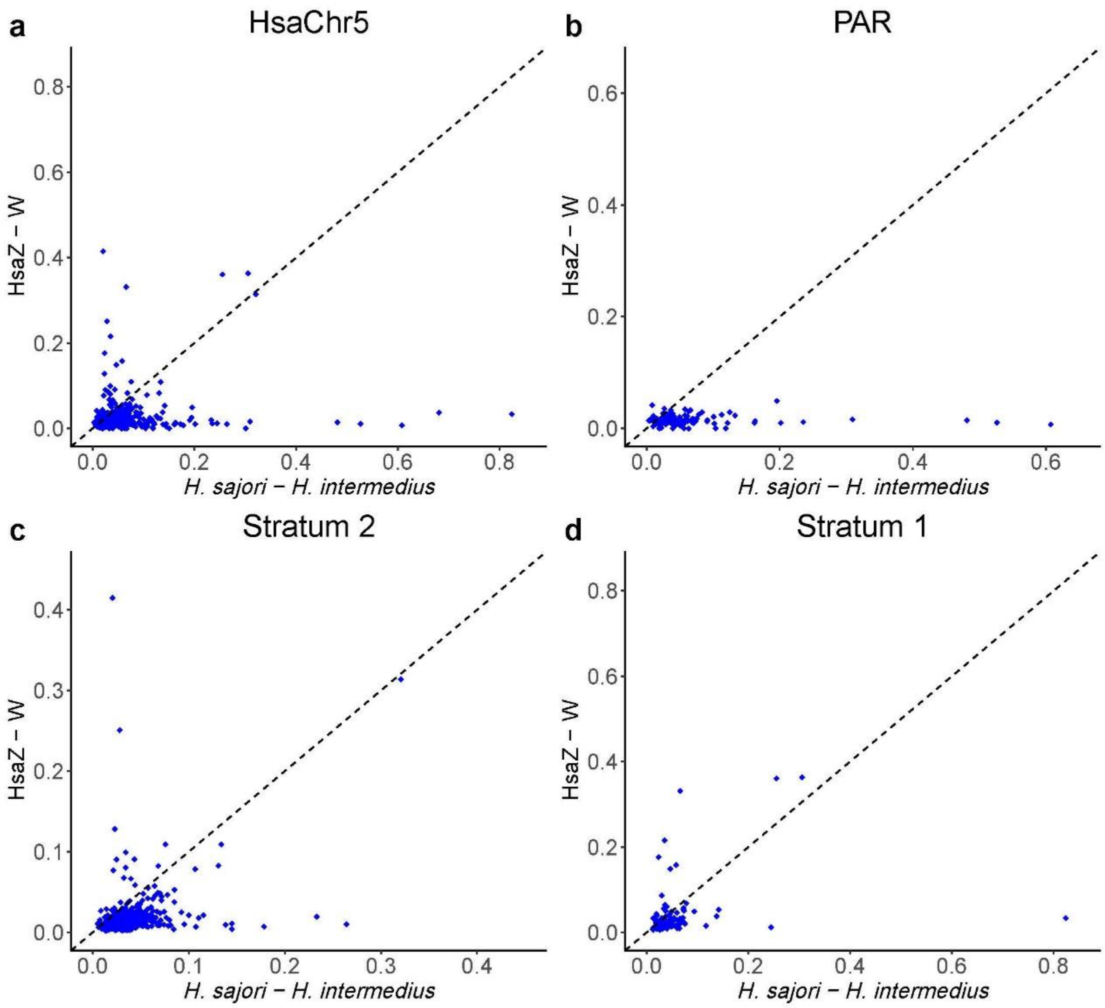

Fig. S18 Synonymous site divergence (*K*s) estimates for inter-species divergence Ks values (on the x axis), and the W-Z *K*s values (on the y axis). The results are shown for the whole chromosome HsaChr5(a) and for the three regions, PAR (b), Stratum2 (c), and Stratum1 (d). The dashed line shows the expectation if the inter-species *K*s and the W-Z values are equal, to highlight the consistently lower values of the latter.

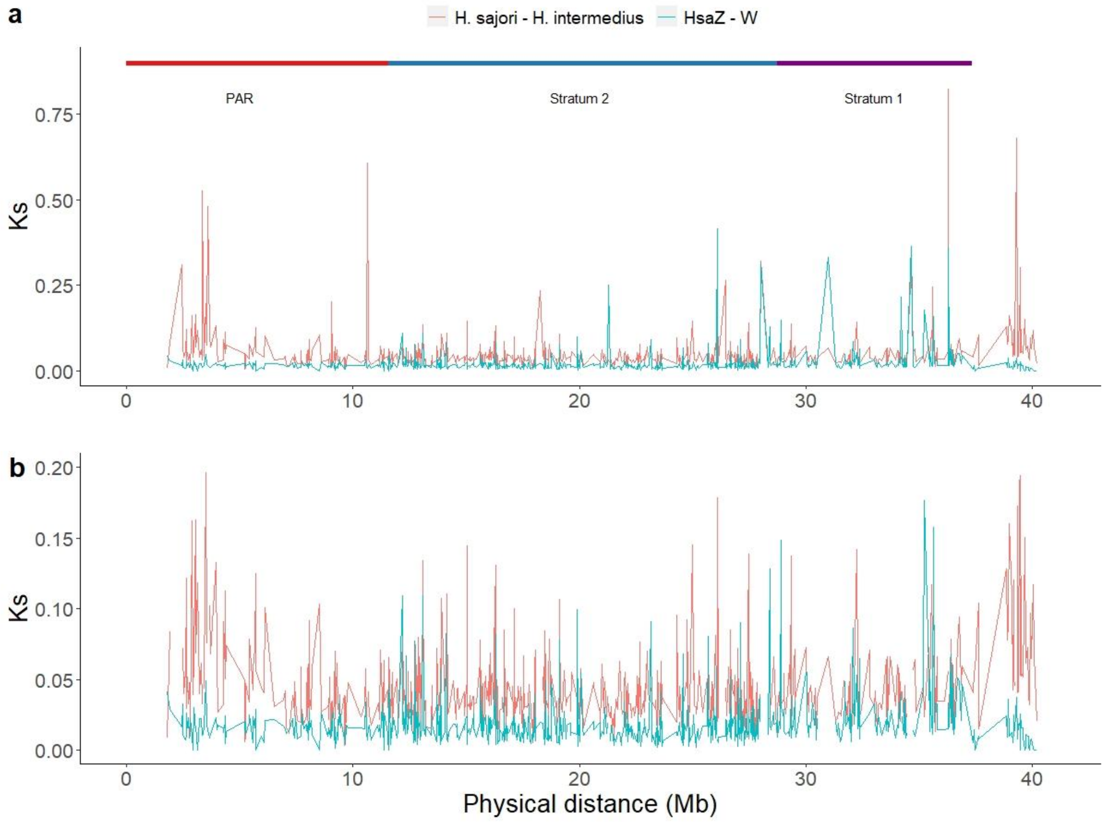

Fig. S19 Synonymous site divergence (*K*s) for inter-species comparisons (red lines) and the W-Z comparison within *H. sajori* (green lines), using homologous gene pairs for both comparisons. Part a shows all the data, and b shows the results for *K*s values < 0.2.

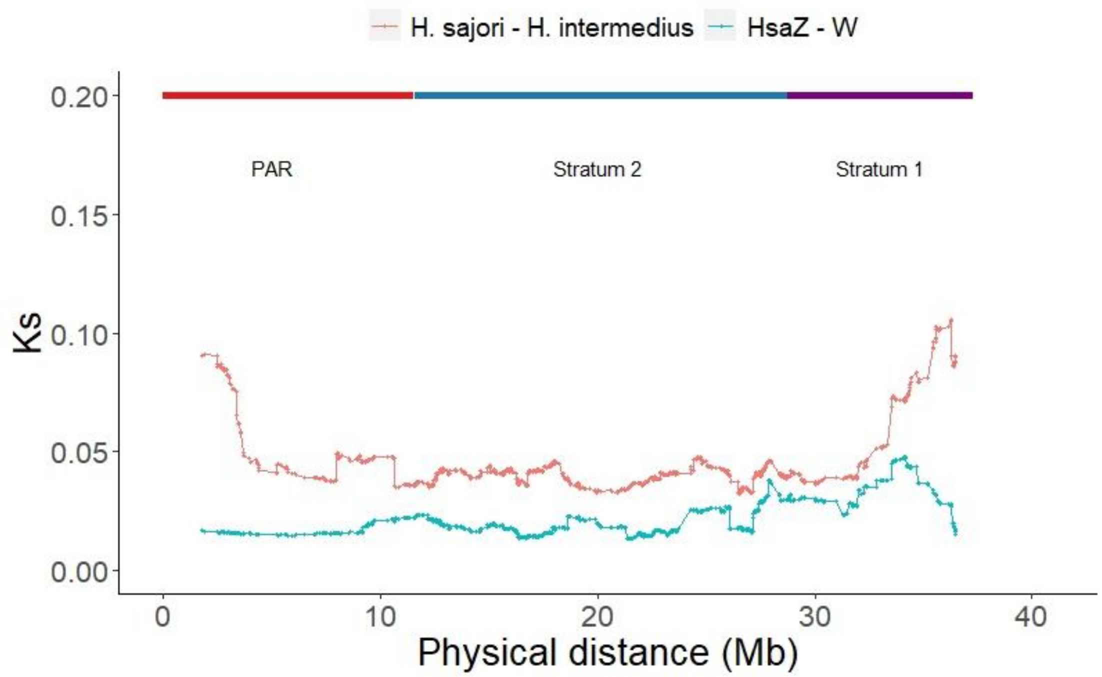

Fig. S20 Synonymous site divergence (*K*s) for the inter-species comparison (red dots) and the *H. sajori* W-Z comparison (green dots), calculated in sliding windows of 50 genes per window, for homologous gene pairs.

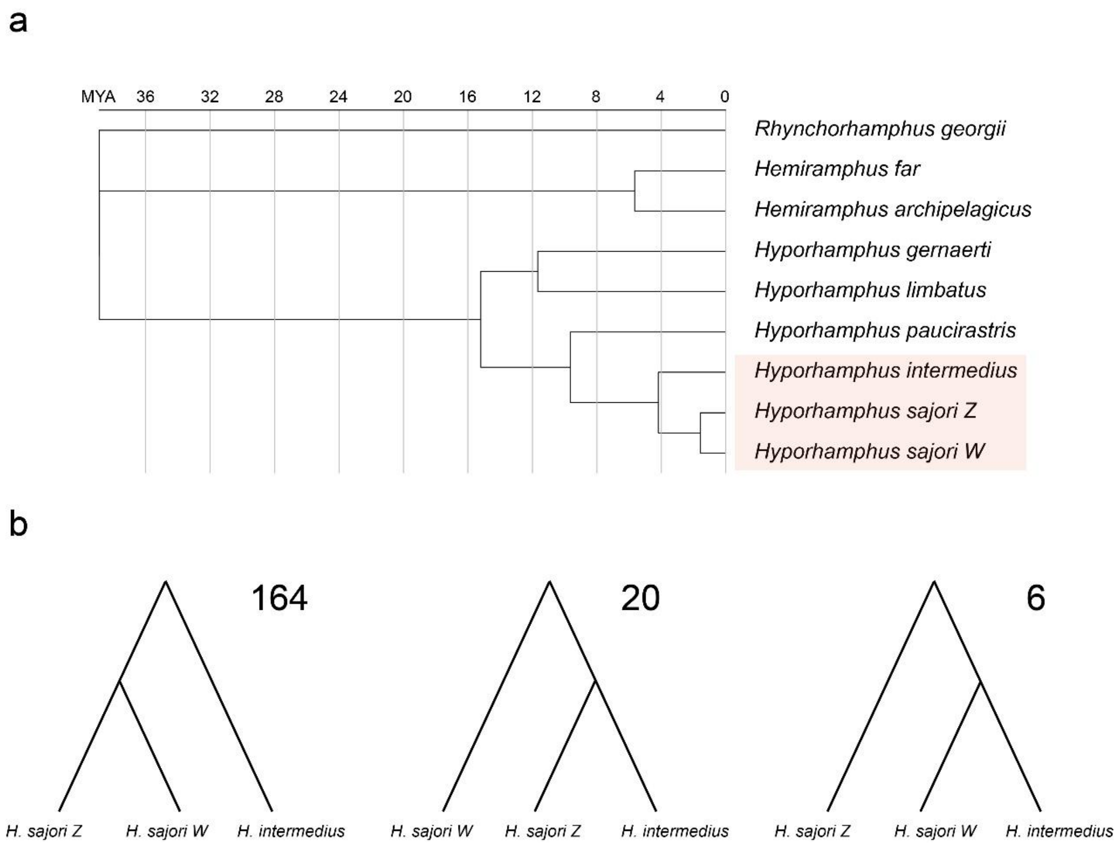

Fig. S21 Relationship of *Hyporhamphus sajori* Z/W gametologs to *H. intermedius*. (a) Phylogeny tree constructed from a concatenated alignment of 190 single copy genes that shared by Z and W of *H. sajori*, *H. intermedius*, *H. gernaerti*, *H. limbatus*, *H. paucirastris*, *Rhynchorhamphus georgii*, *Hemiramphus far* and *Hemiramphus archipelagicus*. (b) Three possible scenarios for the evolution of sex chromosome in *H. sajori*, with counts of three possible tree topologies showing in the upper right.

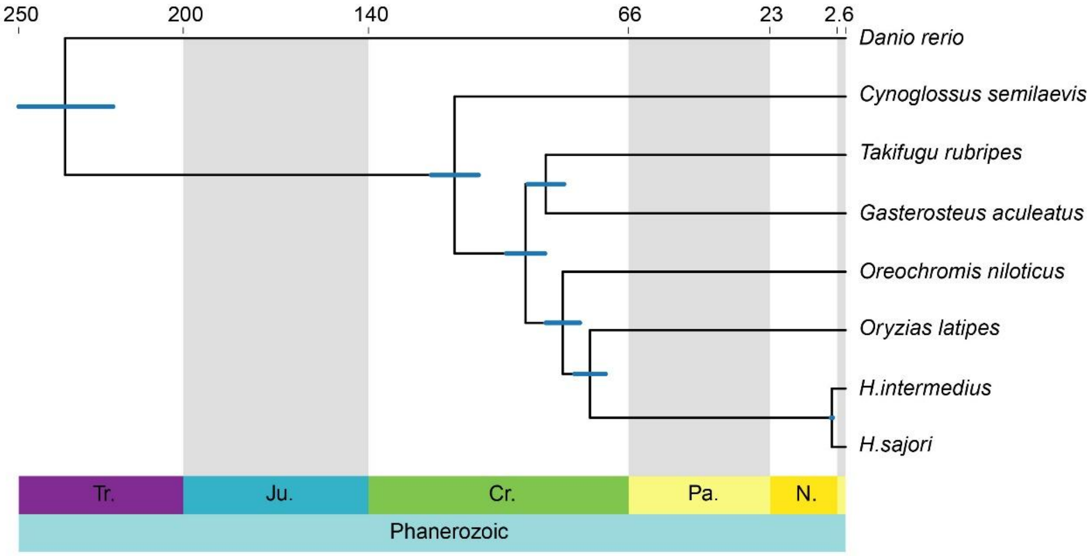

Fig. S22 Phylogenetic tree showing divergence times between *H. sajori*, *H. intermedius* and other six representative teleost species. The phylogenetic tree was constructed using four-fold degenerate sites extracted from single-copy gene families. Calibration point at *O. niloticus* and *O. latipes*: 81-96 Mya; *D. rerio* and *C. semilaevis*: 180-250 Mya.

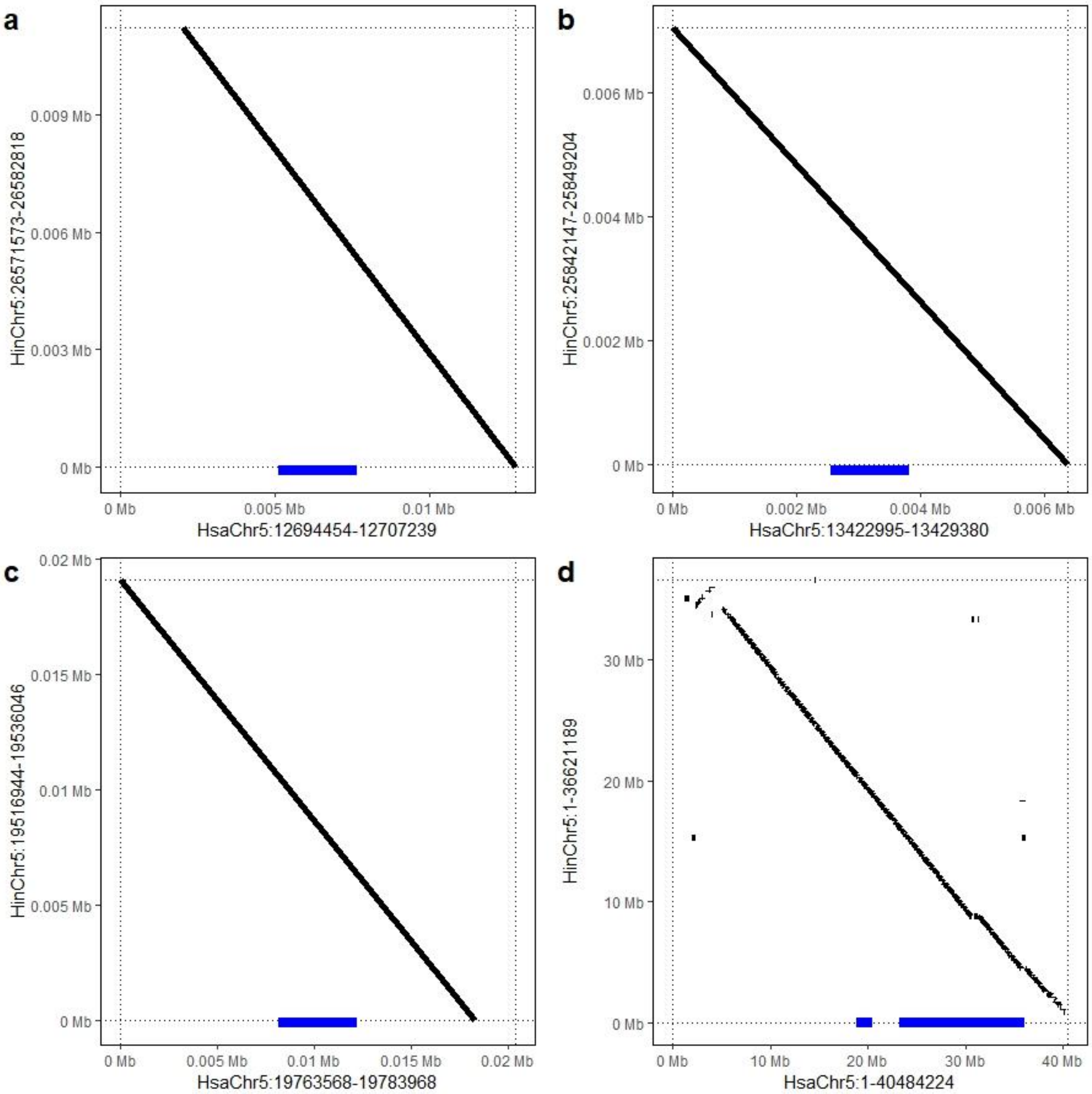

Fig. S23 Synteny plot showing good collinearity between *H. sajori* (HsaChr05) and *H. intermedius* (HinChr05) for the genomic regions around the five inversions that were detected on ChrW. (a), (b) and (c) correspond to the three relatively small inversions named Inv1, Inv2 and Inv4 in Table S11, while (d) corresponds to the two larger Inv3 and Inv5 inversions. Blue segments on the x axis indicate the genome regions spanned by each inversion.

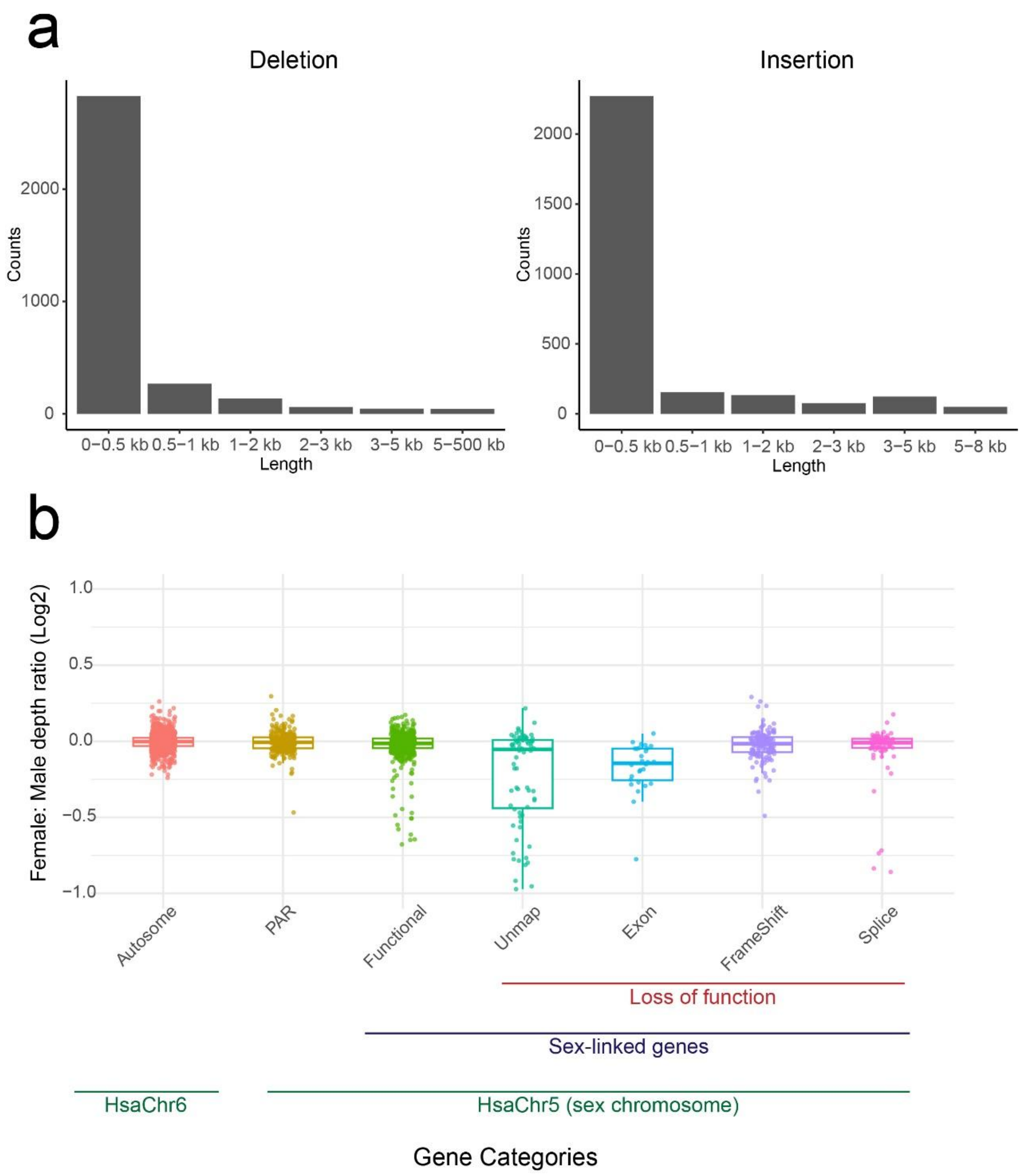

Fig. S24 (a) Length distribution of both deletions and insertions identified in the W of the sequenced female *H. sajori* individual, relative to its Z assembly, using phased PacBio HiFi reads. (b) Coverage depth ratio between females and males for different gene categories (using log2 transformation). Each small dot within the boxplots represents an individual gene. Categories abbreviation: Autosome: HsaChr6 was select as the represents of autosomes. Unmap: genes exhibit significant sequence loss (query coverage <0.5 or/and identity <0.6); Exon: lost more than one exon; FrameShift: genes have lost their start codons and/or are disrupted by premature stop codons or frame-shift mutations; Splice: mutations in splice donor or/and acceptor regions.

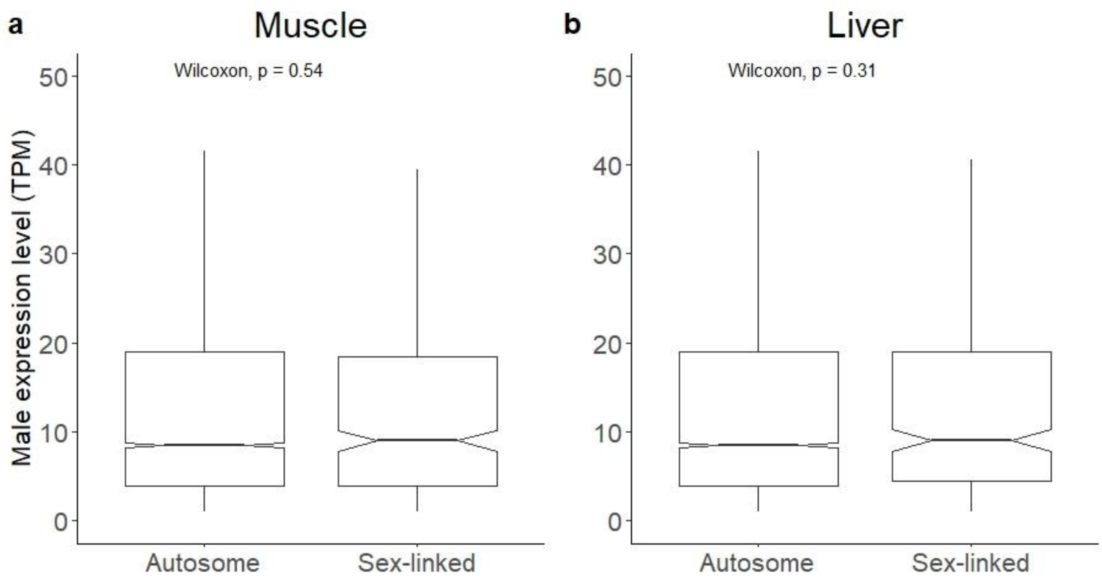

Fig. S25 Expression level estimates (TPM) in *H. sajori* for autosomal genes and genes in the fully sex-linked region, in two tissues of males: (a) muscle and (b) liver. The horizontal lines within each box indicate the median values. The top and bottom of the box indicate the 25th and 75th percentiles, respectively, and whiskers extend 1.5 times the interquartile ranges. Mann–Whitney U tests of the values in the two tissues are not significance (the *P* values are shown).

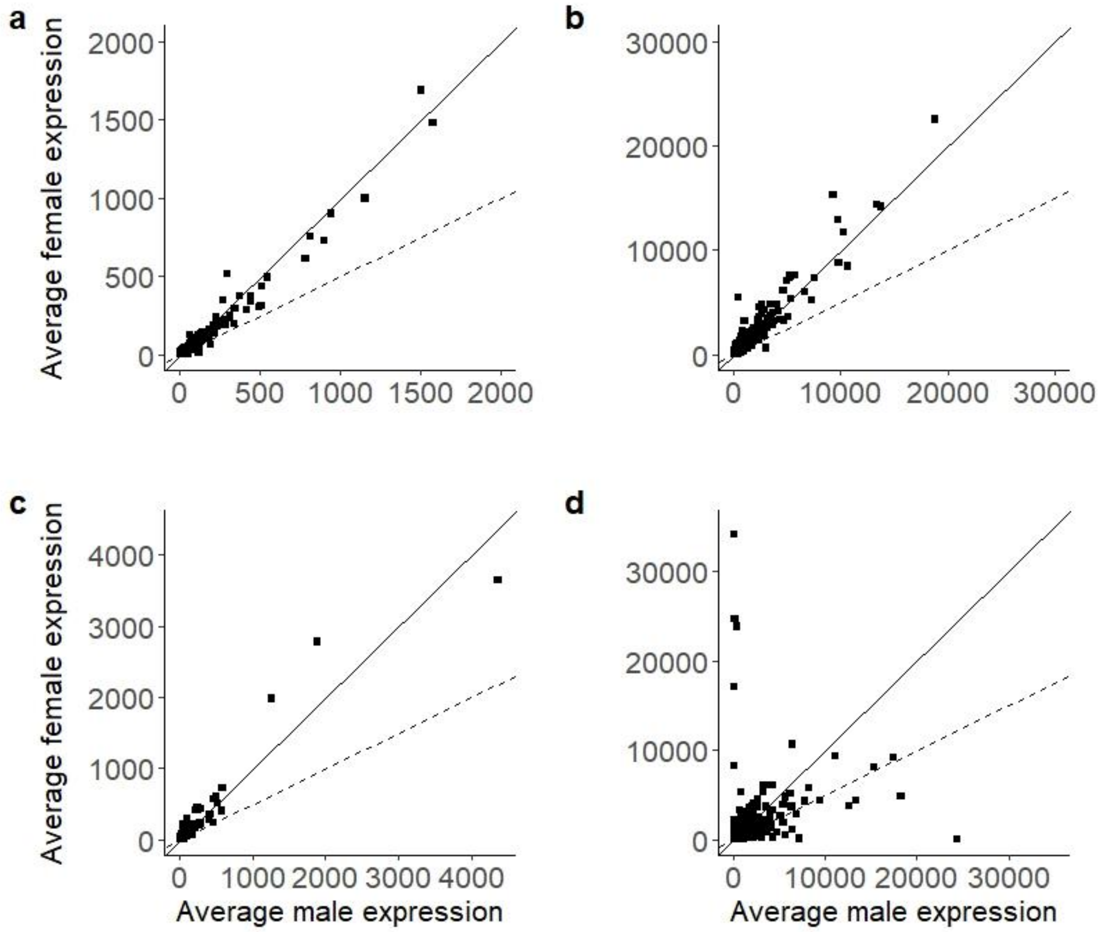

Fig. S26 Average normalized gene expression values (TPM) in females versus males (two of each sex) in muscle (a and b) and liver (c and d). (a, c) Sex-linked genes that with intact ORFs, and (b, d) autosomal genes. The solid line shows the expectation under equal female and male expression, and the dashed line shows the expectation for female expression being equal to one-half of male expression.

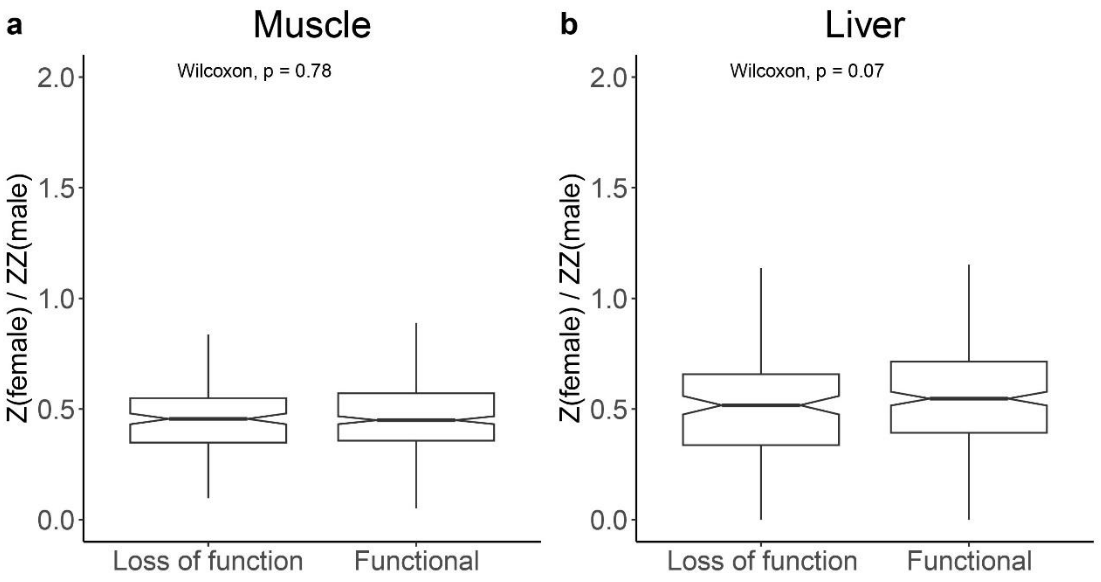

Fig. S27 Transcript abundances of single Z-linked alleles in females (Z female) compared with the two copies in males (ZZ male) for sex-linked genes in muscle (a) and liver (b). Genes with loss of function features, and genes still functional are displayed respectively. The horizontal lines within each box indicate the median values. The top and bottom of the box indicate the 25th and 75th percentiles, respectively, and whiskers extend 1.5 times the interquartile ranges. Mann–Whitney U tests of the values in the two tissues are not significance (the *P* values are shown).

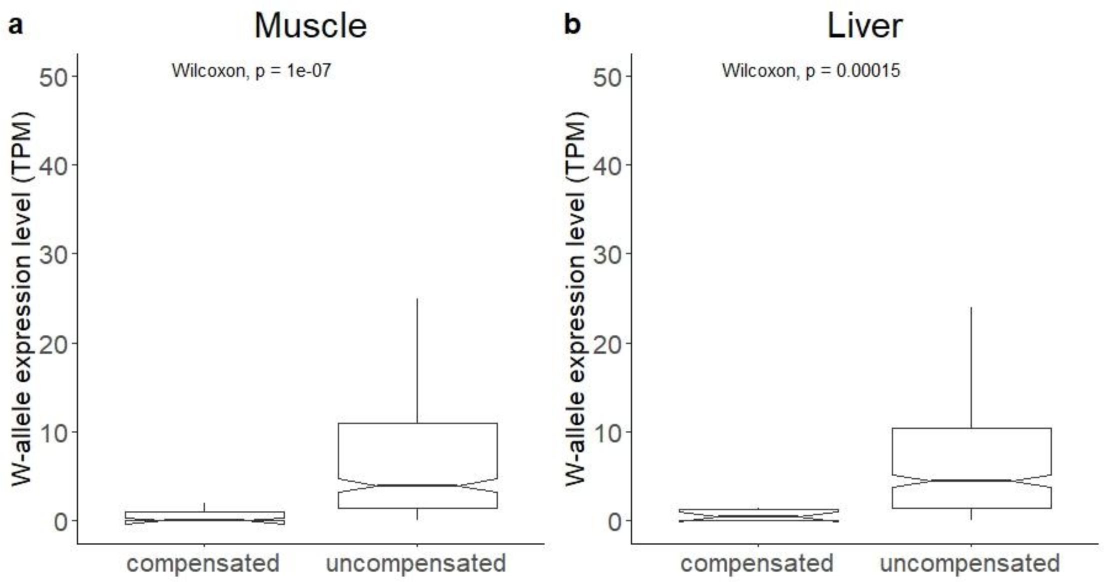

Fig. S28 Expression level estimates (TPM) of W-allele in *H. sajori* for compensated and uncompensated genes in the fully sex-linked region, in two tissues of females: (a) muscle and (b) liver. The horizontal lines within each box indicate the median values. The top and bottom of the box indicate the 25th and 75th percentiles, respectively, and whiskers extend 1.5 times the interquartile ranges. Mann–Whitney U tests of the values in the two tissues are both significant (the *P* values are shown).

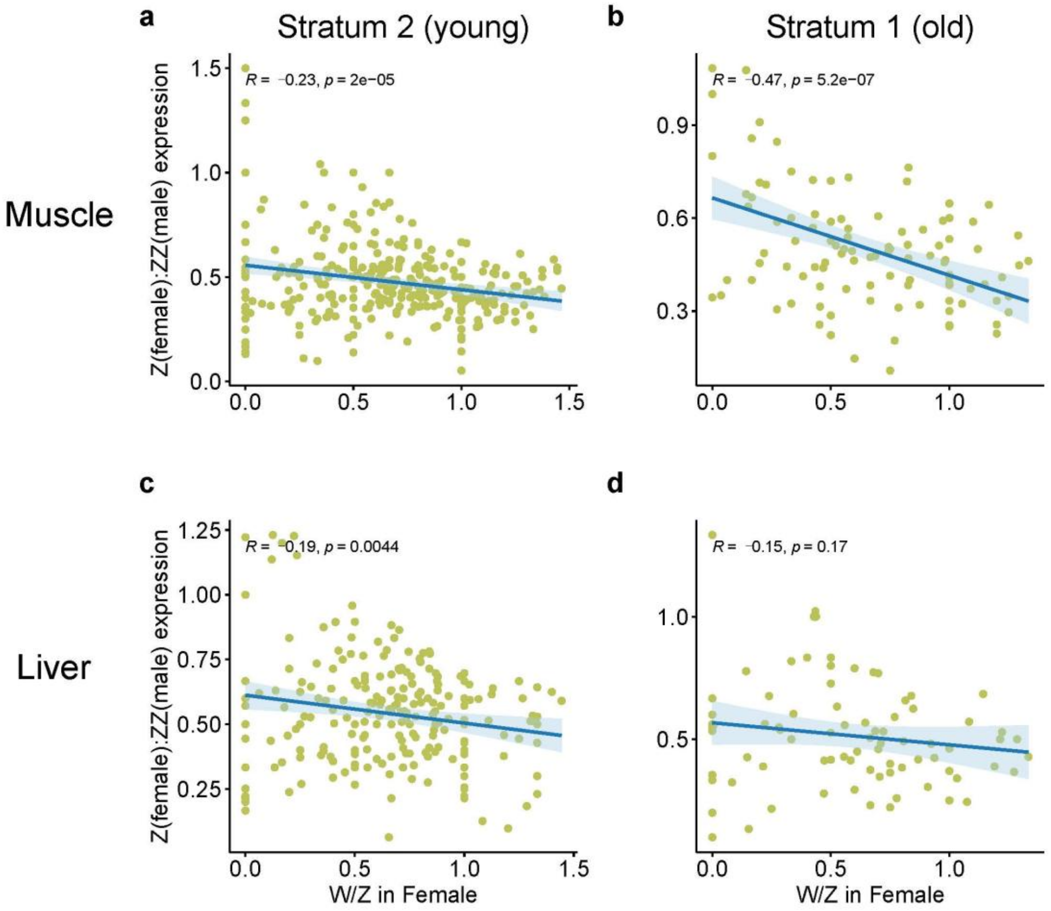

Fig. S29 Expression ratios of single Z-linked alleles in female *H. sajori* individuals, and from two Z chromosomes in males (the y axes), plotted against the ratios of expression of single W- versus Z- linked alleles in females. The results are shown for muscle (a, b) and liver (c, d), for the two strata separately. In the young stratum, genes with lower values of the W/Z ratio (x axis), tend to have higher expression of the Z-linked allele in females, compared with males.

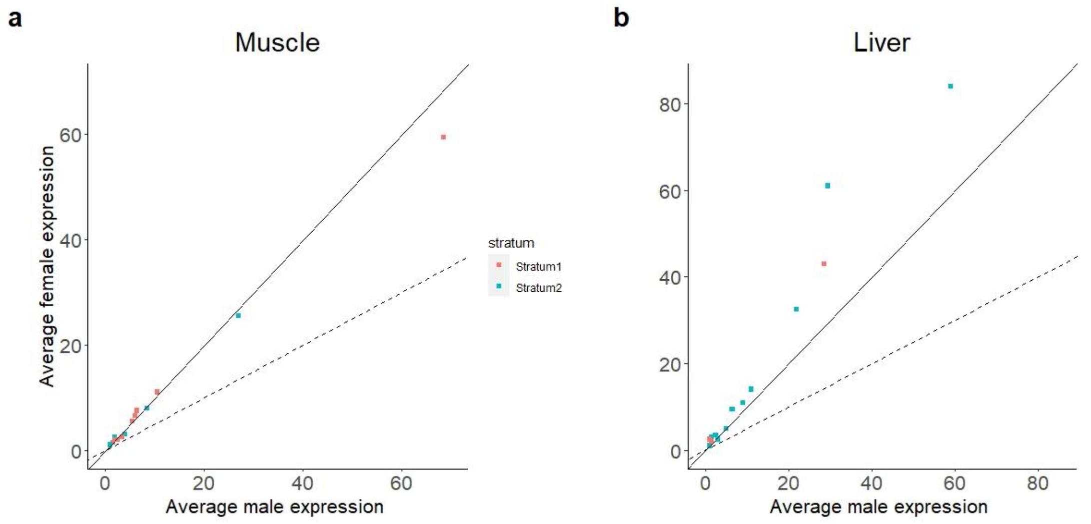

Fig. S30 Average normalized gene expression (TPM) in female vs. males (two of each sex) for possible dosage-compensated genes in muscle (a) and liver (b).

Tables S1 to S16

Table S1 Summary of whole genome resequencing results from six halfbeak species other than *H. intermedius* and *H. sajori*. These species were used in the phylogeny shown in Fig. 1A.

ID	Sex	Species	Platform	Clean_Reads	Clean_Bases	Q20(%)	Q30(%)	GC(%)
wzc22	female	*Rhynchorhamphus georgii*	BGISEQ DNBSEQ-T7	34763081	10.34517049	99.33	97.64	41.81
bzx8	male	*Hemiramphus far*	BGISEQ DNBSEQ-T7	39803979	11.85466267	99.31	97.58	41.9
nyc1	female	*Hemiramphus archipelagicus*	BGISEQ DNBSEQ-T7	36063900	10.73743864	99.23	97.29	42.14
zjf11	female	*H. limbatus*	BGISEQ DNBSEQ-T7	43847227	13.03128546	99.47	98.15	41.24
qzc9	female	*H. gernaerti*	BGISEQ DNBSEQ-T7	43395487	12.8866915	99.49	98.22	39.99
zzzx1	male	*H. paucirastris*	BGISEQ DNBSEQ-T7	38513886	11.50750673	99.34	97.67	41.27

Table S2 Information about the PacBio HiFi reads used for genome assemblies

ID	Sex	Species	Library	Platform	ccs Reads Num	Total ccs Bases (bp)	ccs Reads N50 (bp)	ccs Mean Length (bp)	ccs Longest Read (bp)
1	Female	*H. intermedius*	Pacbio HiFi	Sequel II	1,887,118	29,040,665,968	15,089	15,388	45,260
2	Male	*H. sajori*	Pacbio HiFi	Sequel II	1,604,805	24,519,294,093	15,630	15,278	47,217
3	Female	*H. sajori*	Pacbio HiFi	Revio	1,703,154	24,705,323,751	14,753	14,506	40,397

Table S3 Information about the whole-genome Hi-C library sequencing, genome short-read resequencing and transcriptome sequencing.

ID	Sex	Species	Library	Platform	Base Number	Q30 (%)
1	Female	*H. intermedius*	Hi-C	NovaSeq 6000	76,458,156,600	93.21
			DNA	NovaSeq 6000	93,016,270,200	91.79
			RNA	NovaSeq 6000	12,475,214,400	93.80
2	Male	*H. sajori*	Hi-C	NovaSeq 6000	114,111,944,838	91.79
			DNA	NovaSeq 6000	118,360,661,334	92.31
			RNA	NovaSeq 6000	11,067,403,040	93.46

Table S4 Summary of genome assemblies for male *H. sajori* and female *H. intermedius*.

Assemble	Male *H. sajori*	Female *H. intermedius*
Number of scaffolds	32	141
Number of contigs	487	923
Scaffold total length (bp)	817,670,212	883,980,797
Contig total length (bp)	817,442,712	883,589,797
Scaffold L50	8	8
Scaffold N50 (bp)	36,527,228	36,621,189
Contig L50	19	61
Contig N50 (bp)	14,937,171	3,981,673
Scaffold L90	18	18
Scaffold N90 (bp)	29,455,596	31,828,239
Contig L90	126	257
Contig N90 (bp)	746,289	704,865
Longest scaffold in length	79,088,643	94,951,990
Longest contig in length	44,017,971	16,012,805

Table S5 Chromosome lengths in the two halfbeak species assemblies. The chromosomes of *H. sajori* were assigned ID numbers corresponding to their assemble length from the longest (HsaChr1) to shortest, and the chromosomes of *H. intermedius* were numbered according to their homologies with those of *H. sajori*.

H. sajori		*H. intermedius*		Karyotype
Chr ID	Length (bp)	Chr ID	Length (bp)	M, metacentric; T, telocentric
HsaChr01	79,088,643	HinChr01	94,951,990	T
HsaChr02	76,457,183	HinChr02	76,741,408	T
HsaChr03	68,978,345	HinChr03	72,191,715	T
HsaChr04	56,829,879	HinChr04	58,764,018	T
HsaChr05	40,484,224	HinChr05	36,621,189	T
HsaChr06	39,559,161	HinChr06	40,331,375	T
HsaChr07	37,301,049	HinChr07	36,073,217	T
HsaChr08	36,527,228	HinChr08	38,369,422	T
HsaChr09	34,778,562	HinChr09	36,183,246	T
HsaChr10	34,063,109	HinChr10	35,016,511	T
HsaChr11	34,034,328	HinChr11	33,577,808	T
HsaChr12	33,988,532	HinChr12	34,946,077	T
HsaChr13	32,422,850	HinChr13	33,831,573	T
HsaChr14	32,218,397	HinChr14	33,487,562	T
HsaChr15	31,955,824	HinChr15	57,240,037	T
HsaChr16	31,126,729	HinChr16	32,914,135	M
HsaChr17	30,778,825	HinChr17	34,080,456	T
HsaChr18	29,455,596	HinChr18	30,546,249	T
HsaChr19	28,934,520	HinChr19	31,828,239	T

HsaChr20	28,030,558	HinChr20	31,822,134	T

Table S6 Identification of centromere regions using two methods, QuarTeT and Centier. For the QuarTeT analysis, only the most abundance tandem repeat regions are shown in the table.

	Quartet		Centier	
Chromosome	Range start	End	Range start	End
HsaChr01	77,267,528	78,401,238	74,510,000	79,009,999
HsaChr02	72,587,591	72,926,651	72,330,000	76,329,999
HsaChr03	1	392,138	500,000	3,499,999
HsaChr04	55,727,569	56,698,642	50,333,334	54,166,667
HsaChr05	1,182,244	2,331,979	1,050,000	5,549,999
HsaChr06	1	620,864	37,600,001	39,600,000
HsaChr07	2,976,413	4,794,536	32,933,334	36,766,667
HsaChr08	33,866,858	36,406,378	33,100,001	36,527,228
HsaChr09	33,393,820	34,712,704	833,333	4,666,666
HsaChr10	1	3,565,911	32,910,000	34,063,109
HsaChr11	33,020,608	33,780,374	32,200,001	34,034,328
HsaChr12	32,627,855	33,045,992	27,736,666	32,903,333
HsaChr13	27,999,310	28,364,660	27,700,001	32,422,850
HsaChr14	313,081	1,177,835	29,100,001	32,100,000
HsaChr15	1	584,188	1,166,666	6,833,333
HsaChr16	11,573,489	14,207,307	11,300,001	15,300,000
HsaChr17	30,425,229	30,639,092	26,400,001	30,778,825
HsaChr18	502,395	698,891	0	1,999,999
HsaChr19	28,112,898	28,711,020	27,400,001	28,934,520
HsaChr20	7,644,209	7,814,439	1	1,499,999

Table S7 The length and copy number of the top 5 abundant satellite repeats (100-300 bp) identified in TRASH.

ID	Length	RepeatsN	Sequence
**CenHsa_1**	**207**	**7414**	CAGCGGCTTCAGACGCTTAGAGTTGGAACGTTAGGTGGTTTTGGTGGAAAGCTGTGTAACATGCATTTTCTGTCCCAGCA<br>TCCTTAATTCTTCATGTTGATGGCATAAAACAGTGTTAGAGAGCAGTTTTAGGTGTTTTTGATTGAATTCCATAAGTTTTGC<br>AAAAAGCCCCAAAATCATGTTTTCTGGCCGAGAAACGTCCACATA
**CenHsa_2**	**277**	**6489**	CAGGGTCCGGCCTGGTTAGTACTTGGATGGGAGACCGCCTGGGAATACCAGGTGCTGTAAGCTTTTTATTTGGTAGGAAG<br>CATGACTTTGCCCTGAAATGAAATGATTAAAAGAAATGATTAAAAGGTGGCTTTAGGTCCGAAGACTGACTAATGGGAAG<br>ACGGCGTATAGCTGTTTGATGAATTTTGCATATAAAAGTCAGTTGGAAAGAAGGAACTGTAGCTTACGGCCATACCACCCT<br>GAACACGCCCGATCTCGTCTGATCTCGGAAGCTAAG
CenHsa_3	206	1805	ATGTTGGACGGTGCTGACTGTCCCTGGTCTGCTGCTGTCTGGTCTCTGTCCTCGTCGTTGTGTTCTGTCTCTAATGTTGGAT<br>GTGCTGACTGTCCCTGGTCTGCTGCTGTCTGGTCTCTGTCCTCGTCGTTGTGTTCTGTCTCTAATGTTGGATGCTGACTGTC<br>CTGCTGTCTGGTCTCTGTCCTCGTCGTTGTGTTCTGTCTCTA
CenHsa_4	186	729	TCGTTGTGGTTTGTACAGTTGTTATTGTGGTTGGTGTAGCAGTTGTTGTGGTTGGAGAGGGACTTGATGTGGTTAGTCCTG<br>CAGTTGTCGTGGTTGGTTCACCTATGGTTGTGGTTGAGGCAGCAGTTGTCATAGCTGGTGCAGTAGTAGTTGTCGCCGGG<br>GCTTCTCCGGTGGTTTGTTCTGCAA
CenHsa_5	294	442	CCTTTTTTGTTCTCCCAAGTGGACGGGGTGGTGGAAGGCAAAAAAAAGGAATTGGAAAGAGAGAAACAGCCGCCACAG<br>GGACTTTTATTCTGAAAATGACTTTTGCTTGACAACATTCACTTATTTAATGAATGAAACCAATATTTGCAGTAAATTTTAAT<br>TTTGTTTTTTTGTCTAGACTACATAAAGACTTTAATCCTGCAATGAAACTTTGTATTCATTAAATAAATGAATGTTGTCAAAG<br>TTTTAGCAAATTTTAGCAAAAATAGAGGATTTTTTTGAAAGTAAAAAAT

Table S8 Sampling information for the two halfbeak species.

Loacation	Sex	Species	Number of individuals	Date	Longitude	Latitude
Qingdao, China	male	*H. sajori*	15	2023.4.25	120.34	36.14
Qingdao, China	female	*H. sajori*	12	2023.4.25	120.34	36.14
Weihai, China	male	*H. sajori*	9	2023.5.27	122.16	37.53
Weihai, China	female	*H. sajori*	12	2023.5.27	122.16	37.53
Weishan Lake, China	male	*H. intermedius*	6	2023.6.15	117.24	34.57
Weishan Lake, China	female	*H. intermedius*	6	2023.6.15	117.24	34.57
Fuxian Lake, China	male	*H. intermedius*	6	2023.6.6	102.85	24.62
Fuxian Lake, China	female	*H. intermedius*	6	2023.6.6	102.85	24.62

Table S9 Whole genome resequencing data for samples from different populations of the two halfbeaks.

ID	Sex	Species	Platform	Clean_Reads	Clean_Bases	Q20(%)	Q30(%)	GC(%)
srx1	male	*H. sajori*	BGISEQ DNBSEQ-T7	38,882,382	11,599,331,440	97.39	92.98	40.53
srx2	male	*H. sajori*	BGISEQ DNBSEQ-T7	54,381,139	16,209,467,512	97.15	92.13	41.01
srx3	male	*H. sajori*	BGISEQ DNBSEQ-T7	38,325,220	11,436,157,896	97.46	93.05	40.64
srx4	male	*H. sajori*	BGISEQ DNBSEQ-T7	39,696,458	11,845,744,866	97.89	94.14	40.62
srx5	male	*H. sajori*	BGISEQ DNBSEQ-T7	50,395,109	15,024,740,936	97.61	93.45	40.58
srx6	male	*H. sajori*	BGISEQ DNBSEQ-T7	43,113,982	12,859,300,902	97.33	92.8	40.54
srx7	male	*H. sajori*	BGISEQ DNBSEQ-T7	30,226,857	9,014,041,838	97.13	92.15	40.67
srx8	male	*H. sajori*	BGISEQ DNBSEQ-T7	34,402,047	10,260,032,126	97.89	94.16	40.6
srx9	male	*H. sajori*	BGISEQ DNBSEQ-T7	38,706,982	11,541,542,370	97.69	93.62	40.51
ssx1	male	*H. sajori*	BGISEQ DNBSEQ-T7	44,646,652	13,305,967,788	97.35	92.91	40.54
ssx3	male	*H. sajori*	BGISEQ DNBSEQ-T7	53,972,800	16,076,307,623	97.87	94.23	40.52
ssx4	male	*H. sajori*	BGISEQ DNBSEQ-T7	42,772,060	12,752,538,788	97.55	93.42	40.7
ssx6	male	*H. sajori*	BGISEQ DNBSEQ-T7	44,813,173	13,355,763,810	97.55	93.41	40.43
ssx7	male	*H. sajori*	BGISEQ DNBSEQ-T7	38,281,292	11,413,129,340	97.53	93.37	40.63
ssx8	male	*H. sajori*	BGISEQ DNBSEQ-T7	55,069,581	16,420,695,316	97.58	93.46	40.55
ssx9	male	*H. sajori*	BGISEQ DNBSEQ-T7	34,468,220	10,276,383,116	97.7	93.79	40.49
ssx10	male	*H. sajori*	BGISEQ DNBSEQ-T7	37,038,096	11,041,232,256	97.38	92.91	40.42
ssx11	male	*H. sajori*	BGISEQ DNBSEQ-T7	43,305,205	12,905,304,704	97.65	93.66	40.69
ssx12	male	*H. sajori*	BGISEQ DNBSEQ-T7	37,124,436	11,076,775,214	97.24	92.41	40.66
ssx13	male	*H. sajori*	BGISEQ DNBSEQ-T7	41,932,355	12,495,593,964	97.81	93.97	40.71
ssx14	male	*H. sajori*	BGISEQ DNBSEQ-T7	34,182,694	10,191,566,342	97.17	92.18	40.68
ssx15	male	*H. sajori*	BGISEQ DNBSEQ-T7	41,817,107	12,468,517,706	96.87	91.8	40.66

ssx16	male	*H. sajori*	BGISEQ DNBSEQ-T7	38,550,701	11,501,462,176	97.12	92.27	40.42
ssx17	male	*H. sajori*	BGISEQ DNBSEQ-T7	43,428,120	12,959,071,274	97.28	92.66	40.57
src1	female	*H. sajori*	BGISEQ DNBSEQ-T7	36,701,067	10,945,704,762	97.48	93.07	40.48
src3	female	*H. sajori*	BGISEQ DNBSEQ-T7	38,630,838	11,521,000,364	97.44	93.12	40.52
src4	female	*H. sajori*	BGISEQ DNBSEQ-T7	41,990,900	12,518,211,376	97.3	92.25	40.64
src5	female	*H. sajori*	BGISEQ DNBSEQ-T7	48,405,878	14,429,894,990	97.29	92.26	40.53
src6	female	*H. sajori*	BGISEQ DNBSEQ-T7	43,106,528	12,855,124,236	97.16	91.88	40.5
src8	female	*H. sajori*	BGISEQ DNBSEQ-T7	36,185,733	10,791,364,958	97.34	92.9	40.52
src9	female	*H. sajori*	BGISEQ DNBSEQ-T7	46,142,131	13,762,844,332	97.52	93.31	40.72
src10	female	*H. sajori*	BGISEQ DNBSEQ-T7	41,716,582	12,438,102,314	97.22	92.09	40.57
src11	female	*H. sajori*	BGISEQ DNBSEQ-T7	41,986,552	12,521,601,192	97.45	93.13	40.56
src12	female	*H. sajori*	BGISEQ DNBSEQ-T7	43,811,902	13,073,206,704	97.46	93.18	40.73
src13	female	*H. sajori*	BGISEQ DNBSEQ-T7	47,160,719	14,004,937,864	97.92	94.37	40.28
src14	female	*H. sajori*	BGISEQ DNBSEQ-T7	47,294,852	14,099,960,420	97.6	93.52	40.59
ssc1	female	*H. sajori*	BGISEQ DNBSEQ-T7	45,497,551	13,552,960,552	97.5	93.27	40.56
ssc2	female	*H. sajori*	BGISEQ DNBSEQ-T7	51,223,484	15,271,364,354	97.87	94.29	40.65
ssc3	female	*H. sajori*	BGISEQ DNBSEQ-T7	51,381,136	15,289,690,174	97.31	92.78	40.7
ssc5	female	*H. sajori*	BGISEQ DNBSEQ-T7	33,698,987	10,024,053,176	97.14	92.37	40.76
ssc6	female	*H. sajori*	BGISEQ DNBSEQ-T7	51,395,136	15,279,541,976	97.87	94.27	40.71
ssc7	female	*H. sajori*	BGISEQ DNBSEQ-T7	41,589,142	12,386,577,662	97.78	93.89	40.59
ssc9	female	*H. sajori*	BGISEQ DNBSEQ-T7	43,015,361	12,827,311,302	96.89	91.8	40.37
ssc10	female	*H. sajori*	BGISEQ DNBSEQ-T7	44,102,451	13,147,692,262	97.57	93.43	40.62
ssc11	female	*H. sajori*	BGISEQ DNBSEQ-T7	37,655,392	11,193,139,116	97.82	94.16	40.47
ssc12	female	*H. sajori*	BGISEQ DNBSEQ-T7	55,276,091	16,466,033,849	97.58	93.47	40.66
ssc13	female	*H. sajori*	BGISEQ DNBSEQ-T7	56,550,019	16,805,950,006	97.7	93.8	40.61

ssc14	female	*H. sajori*	BGISEQ DNBSEQ-T7	49,120,504	14,608,768,728	97.76	93.97	40.61
jwx2	male	*H. intermedius*	BGISEQ DNBSEQ-T7	39,448,132	11,750,068,368	97.43	93.06	40.6
jwx3	male	*H. intermedius*	BGISEQ DNBSEQ-T7	43,575,261	12,988,863,658	97.11	92.33	40.47
jwx4	male	*H. intermedius*	BGISEQ DNBSEQ-T7	34,016,532	10,147,088,812	97.14	92.32	40.65
jwx5	male	*H. intermedius*	BGISEQ DNBSEQ-T7	41,048,943	12,224,089,246	98	94.62	40.52
jwx7	male	*H. intermedius*	BGISEQ DNBSEQ-T7	42,554,301	12,687,488,748	97.55	93.37	40.67
jwx8	male	*H. intermedius*	BGISEQ DNBSEQ-T7	45,290,364	13,484,478,944	97.51	93.29	40.49
jfx1	male	*H. intermedius*	BGISEQ DNBSEQ-T7	32,259,145	9,613,854,952	97.35	92.87	40.67
jfx2	male	*H. intermedius*	BGISEQ DNBSEQ-T7	38,134,557	11,370,441,186	97.55	93.39	40.47
jfx3	male	*H. intermedius*	BGISEQ DNBSEQ-T7	43,141,765	12,854,343,840	98.23	95.15	40.56
jfx4	male	*H. intermedius*	BGISEQ DNBSEQ-T7	35,597,968	10,614,965,228	97.43	92.96	40.68
jfx5	male	*H. intermedius*	BGISEQ DNBSEQ-T7	33,205,637	9,906,325,248	97.09	92.17	40.55
jfx6	male	*H. intermedius*	BGISEQ DNBSEQ-T7	40,113,195	11,952,684,348	98.07	94.71	40.3
jwc3	female	*H. intermedius*	BGISEQ DNBSEQ-T7	43,506,746	12,960,687,526	97.1	92.32	40.22
jwc4	female	*H. intermedius*	BGISEQ DNBSEQ-T7	69,869,607	20,729,213,156	97.17	92.2	40.76
jwc5	female	*H. intermedius*	BGISEQ DNBSEQ-T7	35,629,261	10,622,578,604	97.53	93.18	40.6
jwc6	female	*H. intermedius*	BGISEQ DNBSEQ-T7	46,978,462	13,980,531,766	97.58	93.48	40.45
jwc7	female	*H. intermedius*	BGISEQ DNBSEQ-T7	48,427,781	14,412,222,934	97.31	92.77	40.37
jwc8	female	*H. intermedius*	BGISEQ DNBSEQ-T7	43,488,477	12,956,960,814	97.45	93	40.55
jfc3	female	*H. intermedius*	BGISEQ DNBSEQ-T7	39,354,106	11,730,958,920	97.23	92.62	40.3
jfc4	female	*H. intermedius*	BGISEQ DNBSEQ-T7	38,976,328	11,626,217,614	97.28	92.72	40.48
jfc5	female	*H. intermedius*	BGISEQ DNBSEQ-T7	33,490,862	9,992,529,742	97.49	93.06	40.62
jfc6	female	*H. intermedius*	BGISEQ DNBSEQ-T7	44,278,866	13,196,066,226	97.14	92.45	40.51
jfc7	female	*H. intermedius*	BGISEQ DNBSEQ-T7	40,870,045	12,182,364,136	97.69	93.61	40.52
jfc8	female	*H. intermedius*	BGISEQ DNBSEQ-T7	45,332,509	13,497,891,346	97.76	93.82	40.45

Table S10 Estimated repeat contents (total repeat sequence length divided by total length for each region) of the entire *H. sajori* W and Z chromosomes, and the autosomes, showing the higher content of the W (especially for the older Stratum 1 of the W-linked region).

Species	Chromosome			total repeats (%)	DNA (%)	LTR (%)	LINE (%)	Simple (%)	Unknown (%)	Other (%)
	Autosomes			29.82	16.85	5.64	0.26	3.18	3.18	0.71
	HsaChr5 Z			30.97	16.32	5.54	0.21	3.39	4.43	1.08
		Z-PAR		36.75	17.90	3.27	0.14	4.78	8.64	2.03
		Z-SLR		27.51	15.86	6.03	0.23	2.57	2.08	0.75
			Stratum2	19.93	11.95	3.35	0.15	2.40	1.37	0.70
			Stratum1	42.63	23.64	11.36	0.39	2.89	3.50	0.85
	HsaChr5 W			40.56	19.50	12.25	0.08	2.79	5.28	0.66
		W-PAR		31.57	18.44	5.31	0.10	4.33	2.20	1.19
		W-SLR		42.30	19.71	13.59	0.07	2.49	5.88	0.56
			Stratum2	35.83	17.18	10.85	0.06	2.51	4.64	0.59
H. sajori			Stratum1	52.27	23.59	17.82	0.09	2.45	7.79	0.51
H. intermedius	HinChr1-4, 6-20			36.35	19.36	7.70	0.26	2.96	4.70	1.36
	HinChr5			33.05	18.73	5.67	0.19	3.06	4.41	1.00

Table S11 The five large inversions (>1kb) identified on chromosome 5 using phased PacBio HiFi reads of the female *H. sajori* individual,

ID	Chr	Start (bp)	End (bp)	Inversion length (bp)	Number of HiFi reads supporting the inversion
Inv1	chr05	12,699,568	12,702,125	2,557	7
Inv2	chr05	13,425,549	13,426,826	1,277	7
Inv3	chr05	18,807,482	20,518,460	1,710,978	8
Inv4	chr05	19,771,728	19,775,808	4,080	9
Inv5	chr05	23,178,158	36,004,388	12,826,230	5

Table S12 Details of 332 genes with non-functional copies on the W, or lost from the W. Abbreviations in the Effect column are as follows: um: unmapped; ex: at least one exon lost on genes; sl: start lost; sg: stop gain; fr: frame shift; ac: variants in splicing acceptor sites; do: variants in splicing donor sites.

Transcript ID	Effect	Gen ID	Chr	Start	End	Gene_name	Inferred gene product
Hsa_008673-T1	um	Hsa_008673	chr05	11556405	11574194	Uncharacterized	Solute carrier family 26 member 6-like
Hsa_008674-T1	ex	Hsa_008674	chr05	11576459	11599337	SLC26A6	solute carrier family 26 member 6
Hsa_008675-T1	fr	Hsa_008675	chr05	11615688	11645780	MGLL	Monoglyceride lipase
Hsa_008678-T1	fr	Hsa_008678	chr05	11717002	11726065	HYAL3_1	Hyaluronidase
Hsa_008682-T1	um	Hsa_008682	chr05	11746807	11747310	Null	hypothetical protein
Hsa_008689-T1	fr	Hsa_008689	chr05	12197230	12199235	Uncharacterized	Selenoprotein K
Hsa_008694-T1	sg	Hsa_008694	chr05	12320877	12329214	DCP1A	5-(N(7)-methylguanosine 5-triphospho)-[mRNA] hydrolase
Hsa_008695-T1	um	Hsa_008695	chr05	12334759	12338301	Uncharacterized	HECT domain-containing protein
Hsa_008696-T2	fr	Hsa_008696	chr05	12340571	12356545	TKT	Transketolase-like, variant 2
Hsa_008697-T1	fr	Hsa_008697	chr05	12356570	12361773	Uncharacterized	Putative oxidoreductase, chloroplastic
Hsa_008698-T1	fr	Hsa_008698	chr05	12371292	12374331	NSUN5	NOP2/Sun RNA methyltransferase 5
Hsa_38006-T1	ac;fr	Hsa_38006	chr05	12375947	12386165	LOC108891641	Uncharacterized LOC108891641
Hsa_008700-T1	do	Hsa_008700	chr05	12406947	12424359	L3MBTL1	L3MBTL histone methyl-lysine binding protein 1a
Hsa_008712-T1	fr	Hsa_008712	chr05	12579471	12589006	SHMT2_1	Serine hydroxymethyltransferase
Hsa_008715-T2	fr;sg	Hsa_008715	chr05	12605830	12621901	mbd6	Methyl-CpG-binding domain protein 5-like, variant 2
Hsa_008718-T1	sg;fr	Hsa_008718	chr05	12654962	12679277	LOC115585603	Uncharacterized LOC115585603
Hsa_008719-T1	fr	Hsa_008719	chr05	12680959	12689044	TGM2_3	protein-glutamine gamma-glutamyltransferase 2-like isoform X1
Hsa_008721-T1	ex	Hsa_008721	chr05	12723259	12726976	TAS1R1	Taste 1 receptor member 2
Hsa_008725-T1	do	Hsa_008725	chr05	12742428	12803379	UBR4	E3 ubiquitin-protein ligase UBR4 isoform X1
Hsa_008728-T1	sg;fr	Hsa_008728	chr05	12835617	12846149	OLA.11843	Putative oxidoreductase YteT
Hsa_008731-T1	do	Hsa_008731	chr05	12873102	12877878	YBX1	Y-box binding protein 1

Hsa_008739-T1	fr;sg;do	Hsa_008739	chr05	13129258	13135262	TAS1R2C	Taste receptor, type 1, member 2c
Hsa_008740-T1	do	Hsa_008740	chr05	13135716	13142225	RER1	Protein RER1
Hsa_008745-T1	do	Hsa_008745	chr05	13464325	13468500	CELA2A_2	chymotrypsin-like elastase family member 2A
Hsa_008753-T1	do	Hsa_008753	chr05	13767046	13778152	RIMKLA	N-acetylaspartylglutamate synthase
Hsa_008755-T2	ac;fr	Hsa_008755	chr05	13794784	13811123	CCDC30_1	Coiled-coil domain containing 30, variant 2
Hsa_008758-T1	sg	Hsa_008758	chr05	13847906	13857554	TTC34	Tetratricopeptide repeat domain 34
Hsa_008759-T1	sg;fr	Hsa_008759	chr05	13858317	13865568	ALAS2	5-aminolevulinate synthase
Hsa_008761-T1	ex	Hsa_008761	chr05	13896788	13905277	SLC7A5_2	Large neutral amino acids transporter small subunit 1-like
Hsa_008765-T1	do	Hsa_008765	chr05	14099566	14121806	SYCP2	synaptonemal complex protein 2 isoform X1
Hsa_008770-T1	um	Hsa_008770	chr05	14397157	14398554	G5714_009285	JmjC domain-containing protein
Hsa_008772-T1	fr	Hsa_008772	chr05	14502330	14554761	LOC114438435	contactin-3-like
Hsa_008776-T1	fr	Hsa_008776	chr05	14604683	14609938	DFFA	DNA fragmentation factor subunit alpha
Hsa_008777-T1	do	Hsa_008777	chr05	14610769	14662053	PEX14	Peroxisomal membrane protein PEX14
Hsa_008782-T1	do	Hsa_008782	chr05	14945504	15021849	KIF1B	Ubiquitin conjugation factor E4 B isoform X1 (Fragment)
Hsa_008788-T1	do	Hsa_008788	chr05	15148928	15159907	TMEM201	Transmembrane protein 201
Hsa_008791-T1	fr;sg	Hsa_008791	chr05	15277856	15285746	Uncharacterized	PGC-1 and ERR-induced regulator in muscle protein 1
Hsa_38009-T1	do	Hsa_38009	chr05	15309417	15320355	KLHL17	kelch-like protein 17 isoform X1
Hsa_38010-T1	fr	Hsa_38010	chr05	15346486	15347019	Null	hypothetical protein
Hsa_008798-T1	sg	Hsa_008798	chr05	15602017	15607234	CA6	Carbonic anhydrase 6
Hsa_008799-T1	ex	Hsa_008799	chr05	15607455	15615514	SLC2A5	solute carrier family 2, facilitated glucose transporter member 5
Hsa_008806-T3	do	Hsa_008806	chr05	15682164	15685783	EPR50_G00077590	FYVE-type domain-containing protein, variant 3
Hsa_008808-T1	ac;do	Hsa_008808	chr05	15691625	15696393	TRAF3IP3	TRAF3-interacting JNK-activating modulator-like isoform X1
Hsa_008810-T1	do	Hsa_008810	chr05	15703783	15710570	EPS8L3_1	Epidermal growth factor receptor kinase substrate 8-like protein 3
Hsa_008819-T1	fr;sg	Hsa_008819	chr05	15843927	15845962	Uncharacterized	Neugrin
Hsa_008824-T1	um	Hsa_008824	chr05	15918084	15918675	Null	hypothetical protein

Hsa_008826-T1	fr;ac	Hsa_008826	chr05	15939026	15958136	Null	hypothetical protein
Hsa_008830-T1	um	Hsa_008830	chr05	16021951	16023879	PROK1	Prokineticin-1
Hsa_008833-T1	do	Hsa_008833	chr05	16045665	16050069	MMP9	Matrix metalloproteinase-9
Hsa_008841-T1	ex	Hsa_008841	chr05	16272805	16284066	KIAA1324	UPF0577 protein KIAA1324 homolog isoform X1
Hsa_008843-T1	ac	Hsa_008843	chr05	16292690	16295326	LOC106525460	protein FAM107B-like
Hsa_008848-T1	um	Hsa_008848	chr05	16322338	16326074	Uncharacterized	RING-type E3 ubiquitin transferase
Hsa_008852-T1	do	Hsa_008852	chr05	16361083	16372214	PFKM	ATP-dependent 6-phosphofructokinase
Hsa_008857-T1	fr	Hsa_008857	chr05	16467290	16467676	Null	hypothetical protein
Hsa_008858-T1	sg	Hsa_008858	chr05	16470640	16473036	TMPRSS12	Peptidase S1 domain-containing protein
Hsa_008859-T1	ac	Hsa_008859	chr05	16476856	16520556	SCN8A_1	Sodium channel protein
Hsa_008861-T1	fr;do	Hsa_008861	chr05	16589096	16625785	STAT6	Signal transducer and activator of transcription
Hsa_008870-T1	ex	Hsa_008870	chr05	16749697	16757864	GALNT6_1	Polypeptide N-acetylgalactosaminyltransferase
Hsa_008871-T1	sg;fr	Hsa_008871	chr05	16758647	16763177	G6PD_1	Glucose-6-phosphate 1-dehydrogenase
Hsa_008873-T1	do	Hsa_008873	chr05	16770252	16771814	NAA10	N-terminal amino-acid N(alpha)-acetyltransferase NatA
Hsa_008874-T1	do;fr	Hsa_008874	chr05	16773468	16783952	arhgap4	Rho GTPase activating protein 4
Hsa_008880-T1	sl	Hsa_008880	chr05	16897239	16910197	PHF8	PHD finger protein 8
Hsa_008885-T1	do	Hsa_008885	chr05	17051812	17070068	CACNA1S_1	Voltage-dependent L-type calcium channel subunit alpha
Hsa_008888-T2	do	Hsa_008888	chr05	17113998	17124058	MYL6_1	Myosin light polypeptide 6, variant 2
Hsa_008891-T1	ac	Hsa_008891	chr05	17233971	17235747	TAC3	Tachykinin 3
Hsa_38012-T1	fr	Hsa_38012	chr05	17307921	17308866	Null	hypothetical protein
Hsa_008899-T2	fr	Hsa_008899	chr05	17371531	17383616	acvrl1	Receptor protein serine/threonine kinase, variant 2
Hsa_008901-T1	do	Hsa_008901	chr05	17401488	17438720	KMT2D	[Histone H3]-lysine(4) N-methyltransferase
Hsa_008909-T1	ac	Hsa_008909	chr05	17515425	17531114	ERBB3_1	Receptor protein-tyrosine kinase
Hsa_008917-T1	fr	Hsa_008917	chr05	17723465	17736852	FAIM2_1	protein lifeguard 2
Hsa_008918-T1	sg;fr	Hsa_008918	chr05	17737159	17739751	MFSD5	Molybdate-anion transporter

Hsa_008937-T1	fr	Hsa_008937	chr05	18015346	18019018	NFE2	Nuclear factor, erythroid 2
Hsa_008940-T1	sg	Hsa_008940	chr05	18051054	18059302	FQN60_001975	Zgc:
Hsa_008941-T1	ex	Hsa_008941	chr05	18136205	18137882	Null	hypothetical protein
Hsa_008944-T2	do	Hsa_008944	chr05	18370876	18385078	MOV10_1	RNA helicase, variant 2
Hsa_008948-T1	um	Hsa_008948	chr05	18445563	18447716	EBP	3-beta-hydroxysteroid-Delta(8), Delta(7)-isomerase
Hsa_38015-T1	do	Hsa_38015	chr05	18453484	18459418	LOC114439022	uncharacterized protein LOC114439022
Hsa_008952-T1	fr	Hsa_008952	chr05	18464807	18477496	LOC107390940	Obscurin-like
Hsa_008959-T1	sg	Hsa_008959	chr05	18626038	18634691	TNNT2_3	troponin T, cardiac muscle isoform X1
Hsa_008962-T1	do	Hsa_008962	chr05	18687910	18689137	LOC116058552	Adenosine receptor A1-like
Hsa_008967-T1	fr;ac	Hsa_008967	chr05	18762517	18774060	ETV7	Transcription factor ETV7
Hsa_008970-T1	do	Hsa_008970	chr05	18850240	18854604	Null	hypothetical protein
Hsa_008971-T1	ex	Hsa_008971	chr05	18858900	18869098	KCNA3_3	Potassium voltage-gated channel subfamily A member 3
Hsa_008976-T1	do	Hsa_008976	chr05	19096028	19100577	USP21	Ubiquitin carboxyl-terminal hydrolase
Hsa_008978-T1	fr;do	Hsa_008978	chr05	19123575	19125403	LRRC23	Rho-related GTP-binding protein RhoC
Hsa_008985-T1	ac	Hsa_008985	chr05	19215041	19349416	CACNA2D3_1	Voltage-dependent calcium channel subunit alpha-2/delta-3-like
Hsa_008987-T1	sg	Hsa_008987	chr05	19458502	19591011	erc2	ERC protein 2-like
Hsa_008992-T1	fr;sg	Hsa_008992	chr05	19635305	19641094	CYP3A4_1	Cytochrome P450 3A
Hsa_008994-T1	fr	Hsa_008994	chr05	19871637	19871951	Null	hypothetical protein
Hsa_009002-T1	do	Hsa_009002	chr05	19993620	20000291	AMHR2	Receptor protein serine/threonine kinase
Hsa_009005-T1	ex	Hsa_009005	chr05	20045683	20083200	FQA47_008169	Deoxynucleoside triphosphate triphosphohydrolase SAMHD1
Hsa_009006-T1	ex	Hsa_009006	chr05	20124332	20135686	SAMHD1	Deoxynucleoside triphosphate triphosphohydrolase SAMHD1
Hsa_009009-T1	um	Hsa_009009	chr05	20161069	20173299	TP53INP2	Tumor protein p53 inducible nuclear protein 2
Hsa_009010-T1	um	Hsa_009010	chr05	20175152	20184489	GGT7	Glutathione hydrolase
Hsa_009011-T1	um	Hsa_009011	chr05	20185032	20206697	NCOA6	Nuclear receptor coactivator 6
Hsa_38019-T1	um	Hsa_38019	chr05	20209488	20224941	TOP1_3	DNA topoisomerase I

Hsa_009012-T2	um	Hsa_009012	chr05	20226193	20258368	PLCG1_1	1-phosphatidylinositol 4,5-bisphosphate phosphodiesterase gamma, variant 2
Hsa_009013-T1	um	Hsa_009013	chr05	20259389	20272431	ZHX3	Zinc fingers and homeoboxes 3
Hsa_009014-T1	um	Hsa_009014	chr05	20281385	20283576	RAB5IF	RAB5 interacting factor
Hsa_009015-T1	um	Hsa_009015	chr05	20286258	20294655	DHX35	RNA helicase
Hsa_009019-T1	fr;ac	Hsa_009019	chr05	20890553	20891617	Null	hypothetical protein
Hsa_009030-T1	sg	Hsa_009030	chr05	21352718	21399617	SLC2A4RG	SLC2A4 regulator
Hsa_009031-T1	fr	Hsa_009031	chr05	21403746	21412507	LIME1	lck-interacting transmembrane adapter 1 isoform X1
Hsa_009035-T1	fr	Hsa_009035	chr05	21517157	21525950	YTHDF1_1	YTH N6-methyladenosine RNA binding protein 1
Hsa_009036-T1	fr	Hsa_009036	chr05	21559299	21559801	Null	hypothetical protein
Hsa_009038-T1	do	Hsa_009038	chr05	21614287	21622339	SLC17A9	solute carrier family 17 member 9
Hsa_009040-T1	ex	Hsa_009040	chr05	21632976	21669885	DNAH12	Dynein axonemal heavy chain 12
Hsa_009043-T1	do	Hsa_009043	chr05	21744603	21815750	GRM7_1	Metabotropic glutamate receptor 7-like
Hsa_009046-T1	do	Hsa_009046	chr05	21903407	21958446	DOCK3_1	Dedicator of cytokinesis protein 3
Hsa_009052-T1	do	Hsa_009052	chr05	22055516	22074456	ABTB1	Ankyrin repeat and BTB domain containing 1
Hsa_009051-T1	fr	Hsa_009051	chr05	22067334	22067747	Null	hypothetical protein
Hsa_009053-T1	do	Hsa_009053	chr05	22078082	22081797	Uncharacterized	Deoxyribonuclease
Hsa_009055-T1	ex	Hsa_009055	chr05	22093513	22098072	LOC115011228	NACHT, LRR and PYD domains-containing protein 12-like isoform X1
Hsa_009061-T1	sl;fr	Hsa_009061	chr05	22148214	22155426	YWHAB	14-3-3 protein beta/alpha-1
Hsa_009062-T1	sl;sg;fr	Hsa_009062	chr05	22157200	22173270	PABPC1L_1	Polyadenylate-binding protein
Hsa_009064-T1	fr	Hsa_009064	chr05	22178699	22224428	LOC108889499	Solute carrier family 12 member 5-like
Hsa_009076-T1	fr	Hsa_009076	chr05	22552452	22556441	OGG1	DNA-(apurinic or apyrimidinic site) lyase
Hsa_009078-T1	do	Hsa_009078	chr05	22579285	22581976	SULT1ST2_1	Sulfotransferase
Hsa_009079-T1	ex	Hsa_009079	chr05	22582220	22587850	Uncharacterized	Sulfotransferase

Hsa_009083-T1	ex	Hsa_009083	chr05	22622418	22636069	NR2C2	Nuclear receptor subfamily 2 group C member 2
Hsa_009084-T1	fr;do	Hsa_009084	chr05	22640351	22642709	MRPS25	Mitochondrial ribosomal protein S25
Hsa_009086-T1	fr	Hsa_009086	chr05	22659913	22672409	TRH	Pro-thyrotropin-releasing hormone
Hsa_009090-T1	sg;do	Hsa_009090	chr05	22835902	22864186	IFT122	Intraflagellar transport protein 122 homolog
Hsa_009091-T1	fr	Hsa_009091	chr05	22864658	22871051	MBD4	Methyl-CpG binding domain 4, DNA glycosylase
Hsa_009093-T1	fr;do	Hsa_009093	chr05	22882042	22892029	IL17RC	Interleukin 17 receptor C
Hsa_009094-T1	sg;ac	Hsa_009094	chr05	22892859	22902735	IL17RE	Interleukin-17 receptor E-like
Hsa_009096-T1	fr	Hsa_009096	chr05	22917617	22980396	WNK2_1	Non-specific serine/threonine protein kinase
Hsa_009097-T2	sg;fr	Hsa_009097	chr05	22991876	23005550	SUSD3	Sushi domain-containing protein, variant 2
Hsa_009099-T1	fr	Hsa_009099	chr05	23086145	23124987	BICD2_1	Protein bicaudal D homolog 2-like
Hsa_009101-T1	fr	Hsa_009101	chr05	23134753	23137152	FBXW12	F-box/WD repeat-containing protein 12
Hsa_009103-T1	fr	Hsa_009103	chr05	23175820	23176131	Null	hypothetical protein
Hsa_009104-T1	do	Hsa_009104	chr05	23176744	23199599	FBLN2	Fibulin 2
Hsa_009116-T1	do	Hsa_009116	chr05	23476927	23490816	ALAS1_1	5-aminolevulinate synthase
Hsa_009124-T1	fr	Hsa_009124	chr05	23640685	23646602	SS18L1	SS18L1 subunit of BAF chromatin remodeling complex
Hsa_009131-T1	do	Hsa_009131	chr05	24335995	24372848	BYE1	Death-inducer obliterator 1
Hsa_009134-T1	um	Hsa_009134	chr05	24487076	24487736	Null	hypothetical protein
Hsa_009139-T1	ex	Hsa_009139	chr05	24565926	24570706	PTGES3_2	Prostaglandin E synthase 3
Hsa_009141-T1	ac	Hsa_009141	chr05	24586681	24591294	PRIM1	DNA primase
Hsa_009142-T1	sg	Hsa_009142	chr05	24612744	24614367	Null	hypothetical protein
Hsa_009144-T1	fr	Hsa_009144	chr05	24629390	24654685	CACNB3_2	Calcium voltage-gated channel auxiliary subunit beta 3
Hsa_009147-T1	do	Hsa_009147	chr05	24720414	24730551	G6PD_2	Glucose-6-phosphate 1-dehydrogenase
Hsa_009148-T1	do	Hsa_009148	chr05	24730701	24735994	GPD1_1	Glycerol-3-phosphate dehydrogenase [NAD(+)]
Hsa_009151-T1	do	Hsa_009151	chr05	24801927	24816301	GLS2_1	Glutaminase
Hsa_009155-T1	do	Hsa_009155	chr05	24869018	24874459	PCBP2_1	Poly(rC)-binding protein 2-like

Hsa_009156-T1	fr	Hsa_009156	chr05	24899852	24913881	F7725_018255	Zinc finger protein 346
Hsa_009158-T1	fr;sg	Hsa_009158	chr05	25023488	25040919	PFKFB2_1	6-phosphofructo-2-kinase/fructose-2,6-bisphosphatase 2-like
Hsa_009162-T1	ac	Hsa_009162	chr05	25156610	25214187	ANKS1A_1	Ankyrin repeat and sterile alpha motif domain containing 1A
Hsa_38023-T1	fr	Hsa_38023	chr05	25175423	25176166	Null	hypothetical protein
Hsa_009170-T1	fr;do	Hsa_009170	chr05	25654632	25674222	IGFN1_1	Immunoglobulin-like and fibronectin type III domain-containing protein 1
Hsa_009176-T1	do	Hsa_009176	chr05	25791090	25799109	PPP4R2	Serine/threonine-protein phosphatase 4 regulatory subunit 2-A-like
Hsa_009178-T1	fr	Hsa_009178	chr05	25819743	25823941	EXN66_Car012821	DHQ_synthase domain-containing protein
Hsa_009184-T1	ac	Hsa_009184	chr05	25927371	25957864	TAFA1_1	Protein FAM19A1
Hsa_009185-T1	sg	Hsa_009185	chr05	26008605	26014728	Uncharacterized	Solute carrier family 16 member 7
Hsa_009187-T1	ex	Hsa_009187	chr05	26022430	26027606	Uncharacterized	Calcium-independent phospholipase A2-gamma-like
Hsa_009189-T1	ex	Hsa_009189	chr05	26050054	26053213	CHIT1_1	Chitinase
Hsa_009190-T1	fr	Hsa_009190	chr05	26054040	26063914	PM20D1	N-fatty-acyl-amino acid synthase/hydrolase PM20D1
Hsa_009191-T1	fr;sg	Hsa_009191	chr05	26064406	26070235	Uncharacterized	Zgc:92287
Hsa_009194-T1	fr	Hsa_009194	chr05	26104512	26108493	FBXO6	F-box only protein 6-like
Hsa_009200-T1	ac	Hsa_009200	chr05	26496478	26506947	MXRA8_1	Matrix remodeling-associated protein 8
Hsa_009202-T1	sl	Hsa_009202	chr05	26511529	26536516	RAP1GAP_1	RAP1 GTPase activating protein
Hsa_009205-T1	ac	Hsa_009205	chr05	26619965	26623770	APEX2	DNA-(apurinic or apyrimidinic site) endonuclease
Hsa_009217-T2	do	Hsa_009217	chr05	26819879	26864361	Uncharacterized	MAGI family member, X-linked b, variant 2
Hsa_009224-T1	fr	Hsa_009224	chr05	26927472	26940445	PLP2_1	Proteolipid protein 2
Hsa_009237-T1	ex	Hsa_009237	chr05	27102774	27111692	LOC115585045	Sodium/potassium-transporting ATPase subunit beta-1-interacting protein
Hsa_009239-T1	sg	Hsa_009239	chr05	27126699	27134071	LOC100697118	Solute carrier family 17 member 9-like
Hsa_009241-T1	do	Hsa_009241	chr05	27143891	27144777	DNAJC5_1	DnaJ (Hsp40) homolog, subfamily C, member 5aa

Hsa_009245-T2	do	Hsa_009245	chr05	27216239	27227516	EPR50_G00072890	DNA (cytosine-5-)-methyltransferase, variant 2
Hsa_009252-T1	ac;do	Hsa_009252	chr05	27355051	27362493	PTPN1	Tyrosine-protein phosphatase non-receptor type
Hsa_009263-T1	sg	Hsa_009263	chr05	27518156	27521380	OCSTAMP	osteoclast stimulatory transmembrane protein
Hsa_009268-T1	do	Hsa_009268	chr05	27560219	27574393	SPATS2	Spermatogenesis associated serine rich 2
Hsa_009278-T1	fr;do	Hsa_009278	chr05	27726335	27735047	RNF41	E3 ubiquitin-protein ligase NRDP1
Hsa_009284-T1	fr	Hsa_009284	chr05	27820449	27823400	Uncharacterized	Si:ch211-210c8.6
Hsa_009288-T3	fr	Hsa_009288	chr05	27848757	27858911	POU6F1	POU domain protein, variant 3
Hsa_009294-T1	ac;fr	Hsa_009294	chr05	27987261	28010572	D5F01_LYC07954	Death domain-containing protein
Hsa_009296-T1	do	Hsa_009296	chr05	28155450	28328869	PLXNA2_1	Plexin A2
Hsa_009303-T1	fr;sl	Hsa_009303	chr05	28431771	28551839	MAGI3_1	Membrane-associated guanylate kinase, WW and PDZ domain-containing protein 3
Hsa_009306-T1	fr;do	Hsa_009306	chr05	28586845	28703778	HSPG2	basement membrane-specific heparan sulfate proteoglycan core protein isoform X1
Hsa_009310-T1	do	Hsa_009310	chr05	28737330	28738394	Uncharacterized	Trypsin-3-like
Hsa_009312-T1	fr	Hsa_009312	chr05	28810814	28820774	MUL1	RING-type E3 ubiquitin transferase
Hsa_009313-T1	do	Hsa_009313	chr05	28826752	28848016	VWA5B1	von Willebrand factor A domain containing 5B1
Hsa_009315-T2	sg;sl	Hsa_009315	chr05	28876818	28879375	GLTPD1	Ceramide-1-phosphate transfer protein, variant 2
Hsa_009317-T1	sl	Hsa_009317	chr05	28893995	28895680	LOC106536887	lysophosphatidic acid receptor 6-like
Hsa_009319-T1	do	Hsa_009319	chr05	28939636	29010233	ACAP3_1	Arf-GAP with coiled-coil, ANK repeat and PH domain-containing protein
Hsa_009325-T1	ex	Hsa_009325	chr05	29236748	29247762	ZBTB48	telomere zinc finger-associated protein isoform X1
Hsa_009326-T1	sg	Hsa_009326	chr05	29248054	29255406	NOL9	polynucleotide 5-hydroxyl-kinase NOL9
Hsa_009327-T1	do	Hsa_009327	chr05	29296047	29327390	PLEKHG5	Pleckstrin homology and RhoGEF domain containing G5
Hsa_009329-T1	sg	Hsa_009329	chr05	29352205	29354929	Uncharacterized	ANK_REP_REGION domain-containing protein
Hsa_009332-T1	sg;fr	Hsa_009332	chr05	29374662	29414175	CBLN4_1	Formin-like protein 13

Hsa_009339-T1	fr	Hsa_009339	chr05	29574998	29613515	TSHZ2	Teashirt zinc finger homeobox 2
Hsa_009341-T1	um	Hsa_009341	chr05	29721753	29730620	sall4	Spalt like transcription factor 4
Hsa_009342-T1	um	Hsa_009342	chr05	29730380	29760310	ATP9A	Phospholipid-transporting ATPase
Hsa_009343-T1	um	Hsa_009343	chr05	29773172	29787209	NFATC2	Nuclear factor of activated T cells 2
Hsa_009344-T1	um	Hsa_009344	chr05	29823082	29834836	KCNG1	Potassium voltage-gated channel modifier subfamily G member 1
Hsa_009345-T1	um	Hsa_009345	chr05	29836248	29883424	MICAL1	F-actin monooxygenase
Hsa_009346-T1	um	Hsa_009346	chr05	29889142	29898334	CAMK1G_1	Calcium/calmodulin-dependent protein kinase IGb
Hsa_009347-T1	um	Hsa_009347	chr05	29903632	29908278	TAF8	Transcription initiation factor TFIID subunit 8
Hsa_009348-T1	um	Hsa_009348	chr05	29907686	29910272	PIFO	Primary cilia formation
Hsa_009349-T1	um	Hsa_009349	chr05	29911306	29917528	ATP6AP1_1	V-type proton ATPase subunit S1
Hsa_009350-T1	um	Hsa_009350	chr05	29918752	29923931	GDI1_1	Rab GDP dissociation inhibitor
Hsa_009351-T1	um	Hsa_009351	chr05	29923998	29950261	ITIH6	Inter-alpha-trypsin inhibitor heavy chain family member 6
Hsa_009352-T1	um	Hsa_009352	chr05	29950134	29969808	PFKFB1_1	6PF2K domain-containing protein
Hsa_009355-T1	ex	Hsa_009355	chr05	30023925	30055995	ARHGEF10L_1	Rho guanine nucleotide exchange factor 10-like protein
Hsa_009357-T1	do	Hsa_009357	chr05	30074777	30123167	VPS13D	LOW QUALITY PROTEIN: vacuolar protein sorting-associated protein 13D
Hsa_009359-T1	fr;sg	Hsa_009359	chr05	30136230	30136574	Null	hypothetical protein
Hsa_009360-T1	fr	Hsa_009360	chr05	30137814	30180957	EPHB2_1	Receptor protein-tyrosine kinase
Hsa_009367-T1	ex	Hsa_009367	chr05	30494531	30509750	POFUT1	GDP-fucose protein O-fucosyltransferase 1
Hsa_009368-T1	um	Hsa_009368	chr05	30520821	30522175	KLHL7_1	Kelch-like family member 7
Hsa_009369-T1	um	Hsa_009369	chr05	30539008	30540321	KLHL7_2	Kelch-like family member 7
Hsa_009370-T1	um	Hsa_009370	chr05	30576522	30577876	KLHL7_3	Kelch-like family member 7
Hsa_009371-T1	um	Hsa_009371	chr05	30609473	30610827	KLHL7_4	Kelch-like family member 7
Hsa_009372-T1	um	Hsa_009372	chr05	30624700	30626013	KLHL7_5	Kelch-like family member 7
Hsa_009373-T1	um	Hsa_009373	chr05	30643515	30644828	KLHL7_6	Kelch-like family member 7

Hsa_009374-T1	um	Hsa_009374	chr05	30656033	30657469	KLHL7_7	Kelch-like family member 7
Hsa_009375-T1	um	Hsa_009375	chr05	30669085	30670603	KLHL7_8	Kelch-like family member 7
Hsa_009376-T1	um	Hsa_009376	chr05	30688130	30689484	KLHL7_9	Kelch-like family member 7
Hsa_009377-T1	um	Hsa_009377	chr05	30706187	30707179	KLHL7_10	Kelch-like family member 7
Hsa_009378-T1	um	Hsa_009378	chr05	30723895	30724887	KLHL7_11	Kelch-like family member 7
Hsa_009379-T1	um	Hsa_009379	chr05	30741479	30742471	KLHL7_12	Kelch-like family member 7
Hsa_009380-T1	um	Hsa_009380	chr05	30759758	30761112	KLHL7_13	Kelch-like family member 7
Hsa_009381-T1	um	Hsa_009381	chr05	30775073	30776427	KLHL7_14	Kelch-like family member 7
Hsa_009382-T1	um	Hsa_009382	chr05	30801944	30810539	KLHL7_15	Kelch-like family member 7
Hsa_009383-T1	um	Hsa_009383	chr05	30815054	30816367	KLHL7_16	Kelch-like family member 7
Hsa_009384-T1	um	Hsa_009384	chr05	30834252	30835560	KLHL7_17	Kelch-like family member 7
Hsa_009385-T1	um	Hsa_009385	chr05	30853477	30854790	KLHL7_18	Kelch-like family member 7
Hsa_009386-T1	um	Hsa_009386	chr05	30873019	30874332	KLHL7_19	Kelch-like family member 7
Hsa_009387-T1	um	Hsa_009387	chr05	30911759	30913113	KLHL7_20	Kelch-like family member 7
Hsa_009388-T1	um	Hsa_009388	chr05	30924751	30926064	KLHL7_21	Kelch-like family member 7
Hsa_009389-T1	um	Hsa_009389	chr05	30956298	30957693	KLHL7_22	Kelch-like family member 7
Hsa_009390-T1	um	Hsa_009390	chr05	30970755	30974798	Null	hypothetical protein
Hsa_009391-T1	um	Hsa_009391	chr05	30983284	30984597	KLHL7_23	Kelch-like family member 7
Hsa_009392-T1	um	Hsa_009392	chr05	30996166	30997684	KLHL7_24	Kelch-like family member 7
Hsa_009412-T1	fr	Hsa_009412	chr05	31298503	31377514	KIF3B	Kinesin-like protein
Hsa_009414-T1	fr	Hsa_009414	chr05	31404067	31455908	MYT1_1	Myelin transcription factor 1
Hsa_009415-T1	fr;sg	Hsa_009415	chr05	31523009	31543032	TBC1D22B	TBC1 domain family member 22B
Hsa_009417-T2	fr;sg;do	Hsa_009417	chr05	31562958	31583139	CCH79_00016163	Chloroplast protein-transporting ATPase, variant 2
Hsa_009419-T1	sg;fr;ac	Hsa_009419	chr05	31722591	31732889	MFN2_1	Mitofusin 2
Hsa_009420-T1	fr;ac	Hsa_009420	chr05	31736184	31746462	MFN2_2	mitofusin-2

Hsa_009422-T1	fr	Hsa_009422	chr05	31791152	31793457	Null	hypothetical protein
Hsa_009426-T1	ex	Hsa_009426	chr05	31865033	31871569	LOC108900483	putative beta-lactamase-like 1
Hsa_009427-T1	ex	Hsa_009427	chr05	31894705	31905206	FOXP3	forkhead box protein P3-like
Hsa_009429-T1	fr;sg	Hsa_009429	chr05	31935011	31939536	Uncharacterized	Tripartite motif-containing protein 35-like
Hsa_009430-T1	fr	Hsa_009430	chr05	31942449	31944061	Uncharacterized	Tripartite motif-containing protein 35-like
Hsa_009431-T1	fr;sg	Hsa_009431	chr05	31947420	31952595	Uncharacterized	Tripartite motif-containing protein 35-like
Hsa_009438-T1	ex	Hsa_009438	chr05	32231689	32250872	OJAV_G00136050	IG domain-containing protein
Hsa_009442-T1	fr	Hsa_009442	chr05	32351787	32356328	gata1	erythroid transcription factor-like
Hsa_009446-T1	fr	Hsa_009446	chr05	32406632	32421924	Uncharacterized	Leucine-rich repeat neuronal protein 1-like
Hsa_009447-T1	um	Hsa_009447	chr05	32434349	32456938	OTUD5	Ubiquitinyl hydrolase 1
Hsa_009448-T1	um	Hsa_009448	chr05	32461067	32464136	PIM2	Serine/threonine-protein kinase
Hsa_009449-T1	um	Hsa_009449	chr05	32466648	32482558	SLC35A2	Solute carrier family 35 member A2
Hsa_009450-T1	um	Hsa_009450	chr05	32498448	32548986	SLC6A8_3	Transporter
Hsa_009451-T1	um	Hsa_009451	chr05	32563307	32590084	BCAP31	Endoplasmic reticulum transmembrane protein
Hsa_38036-T1	sg;fr	Hsa_38036	chr05	32563985	32564625	LOC109899784	Equilibrative nucleoside transporter 1
Hsa_38037-T1	um	Hsa_38037	chr05	32579200	32579888	Null	hypothetical protein
Hsa_009452-T1	fr	Hsa_009452	chr05	32635847	32755150	PLXNA3_1	plexin-A3
Hsa_009453-T1	um	Hsa_009453	chr05	32743885	32746195	Null	hypothetical protein
Hsa_009454-T1	um	Hsa_009454	chr05	32774540	32776521	Null	hypothetical protein
Hsa_009455-T2	um	Hsa_009455	chr05	32776624	32808293	PTPN18	Tyrosine-protein phosphatase non-receptor type 18, variant 2
Hsa_009461-T1	fr	Hsa_009461	chr05	33012517	33014256	Null	hypothetical protein
Hsa_009462-T1	ac	Hsa_009462	chr05	33016297	33043318	ST14_2	Putative suppressor of tumorigenicity 14 protein-like
Hsa_009463-T1	fr	Hsa_009463	chr05	33057428	33086786	PTPN7	Protein-tyrosine-phosphatase
Hsa_009466-T1	fr;sg	Hsa_009466	chr05	33160643	33243962	ITPR3	Inositol 1,4,5-trisphosphate receptor
Hsa_009467-T1	fr	Hsa_009467	chr05	33277869	33314918	MYH7B_1	Myosin-7B-like

Hsa_009468-T1	sg;fr	Hsa_009468	chr05	33314929	33319917	MYH7B_2	Myosin-7B-like
Hsa_009470-T1	do;fr	Hsa_009470	chr05	33360645	33452576	Null	hypothetical protein
Hsa_009475-T1	sg	Hsa_009475	chr05	33595015	33596982	Null	hypothetical protein
Hsa_009478-T1	fr;ac	Hsa_009478	chr05	33651867	33683499	PI16	peptidase inhibitor 16-like
Hsa_009479-T1	fr;ac	Hsa_009479	chr05	33709801	33717908	EDEM2	alpha-1,2-Mannosidase
Hsa_009481-T1	sg	Hsa_009481	chr05	33778907	33790358	PFLUV_G00064670	Polymeric immunoglobulin receptor-like
Hsa_009483-T1	fr;sg	Hsa_009483	chr05	33903807	33910239	SMAX5B_014657	Putative IQ motif and SEC7 domain-containing protein 2-like (Fragment)
Hsa_009489-T1	fr	Hsa_009489	chr05	34114977	34121676	E1301_Tti021365	Zinc finger protein 501-like
Hsa_009492-T1	fr	Hsa_009492	chr05	34172107	34197065	CLCN6	Chloride channel protein
Hsa_009493-T1	fr	Hsa_009493	chr05	34201814	34209747	Uncharacterized	Rhamnose-binding lectin
Hsa_009497-T1	do	Hsa_009497	chr05	34310393	34324720	CCDC22	Coiled-coil domain-containing protein 22
Hsa_009504-T1	ac	Hsa_009504	chr05	34421175	34439401	PFLUV_G00063490	Guanylate cyclase domain-containing protein
Hsa_009507-T1	do;fr	Hsa_009507	chr05	34492071	34504808	Uncharacterized	Pentaxin
Hsa_009510-T1	fr;sg;do	Hsa_009510	chr05	34811807	34821213	FAAP20	UBZ2-type domain-containing protein
Hsa_009514-T1	sg	Hsa_009514	chr05	35247925	35399688	PLCH2_2	Phosphoinositide phospholipase C
Hsa_009515-T1	ex	Hsa_009515	chr05	35421074	35425653	Null	hypothetical protein
Hsa_009517-T1	do	Hsa_009517	chr05	35448709	35467282	STK38	Serine/threonine kinase 38
Hsa_009519-T1	sg	Hsa_009519	chr05	35535072	35544156	LOC115585494	Insulin-like growth factor I
Hsa_009522-T1	fr	Hsa_009522	chr05	35567726	35582670	LRIF1	Ligand dependent nuclear receptor interacting factor 1
Hsa_009524-T1	fr;do	Hsa_009524	chr05	35600334	35602472	LOC114431859	uncharacterized protein LOC114431859
Hsa_009525-T1	sg	Hsa_009525	chr05	35605407	35613624	MTCH1	Mitochondrial carrier 1
Hsa_009527-T1	sg	Hsa_009527	chr05	35705269	35706354	PDYN	Proenkephalin-B
Hsa_009528-T1	ac	Hsa_009528	chr05	35718181	35729625	TPX2_1	TPX2 microtubule nucleation factor
Hsa_009529-T1	ac	Hsa_009529	chr05	35739745	35748282	TPX2_2	TPX2 microtubule nucleation factor

Hsa_009530-T1	fr;ac	Hsa_009530	chr05	35753329	35763331	TPX2_3	TPX2 microtubule nucleation factor
Hsa_009532-T1	do;fr	Hsa_009532	chr05	35783845	35790291	Uncharacterized	Nudix hydrolase 20, chloroplastic-like
Hsa_009533-T1	ac	Hsa_009533	chr05	35790097	35800305	GMEB2	Glucocorticoid modulatory element binding protein 2
Hsa_009534-T1	ac	Hsa_009534	chr05	35806333	35818918	TPX2_5	TPX2 microtubule nucleation factor
Hsa_009535-T1	ac	Hsa_009535	chr05	36118339	36130988	TPX2_6	TPX2 microtubule nucleation factor
Hsa_009537-T1	ex	Hsa_009537	chr05	36131713	36253493	PLOD1	Procollagen-lysine 5-dioxygenase
Hsa_009536-T1	ex	Hsa_009536	chr05	36140199	36152179	TPX2_7	TPX2 microtubule nucleation factor
Hsa_009538-T1	fr	Hsa_009538	chr05	36256256	36264482	KIAA2013	KIAA2013 ortholog
Hsa_009539-T1	sg;fr	Hsa_009539	chr05	36267716	36268057	Null	hypothetical protein
Hsa_009541-T1	fr	Hsa_009541	chr05	36268076	36276885	PHC2_1	Polyhomeotic-like protein 2
Hsa_009540-T1	fr	Hsa_009540	chr05	36268303	36268626	Null	hypothetical protein
Hsa_009542-T1	fr;do	Hsa_009542	chr05	36275804	36288078	D5F01_LYC23798	DBD_Tnp_Mut domain-containing protein
Hsa_009543-T1	um	Hsa_009543	chr05	36288523	36295687	CFAP20_3	cilia- and flagella-associated protein 20 isoform X1
Hsa_009544-T1	um	Hsa_009544	chr05	36298508	36301572	Null	hypothetical protein
Hsa_009546-T1	do	Hsa_009546	chr05	36382710	36391730	COMMD7	COMM domain containing 7
Hsa_009547-T1	fr	Hsa_009547	chr05	36405544	36412083	OJAV_G00071480	Protein FAM107B
Hsa_009550-T1	fr	Hsa_009550	chr05	36434507	36437845	Uncharacterized	Zinc finger protein 14-like
Hsa_009551-T1	fr	Hsa_009551	chr05	36438533	36438856	Null	hypothetical protein
Hsa_009553-T1	fr	Hsa_009553	chr05	36446679	36450176	PLPBP	Pyridoxal phosphate homeostasis protein
Hsa_009557-T1	fr	Hsa_009557	chr05	36489560	36490448	Null	hypothetical protein
Hsa_38040-T1	sl	Hsa_38040	chr05	36499394	36500099	Null	hypothetical protein
Hsa_009559-T1	fr;ac	Hsa_009559	chr05	36522432	36529468	LOC116058000	Alpha-1,3-mannosyl-glycoprotein 4-beta-N-acetylglucosaminyltransferase C-like
Hsa_009562-T1	do;sg;fr	Hsa_009562	chr05	36555360	36583216	EXN66_Car012156	Cyclin-Q
Hsa_009565-T1	um	Hsa_009565	chr05	36655438	36666639	HAUS7_1	HAUS augmin-like complex subunit 7

Hsa_009566-T1	um	Hsa_009566	chr05	36667044	36668964	MRPL49_1	39S ribosomal protein L49, mitochondrial
Hsa_009567-T1	um	Hsa_009567	chr05	36669180	36672451	HAUS7_2	HAUS augmin-like complex subunit 7
Hsa_009568-T1	fr	Hsa_009568	chr05	36672843	36673376	MRPL49_2	39S ribosomal protein L49, mitochondrial
Hsa_009570-T1	do	Hsa_009570	chr05	36676799	36682458	Uncharacterized	Ninjurin-1-like
Hsa_009571-T1	do	Hsa_009571	chr05	36689362	36736085	FLNA	filamin-A isoform X1
Hsa_009572-T1	ex	Hsa_009572	chr05	36792551	36798238	Null	hypothetical protein
Hsa_009573-T1	ex	Hsa_009573	chr05	36836786	36854878	EXN66_Car013054	DNA (cytosine-5-)-methyltransferase
Hsa_009574-T1	fr	Hsa_009574	chr05	36869347	36882061	MAPRE1	Microtubule-associated protein RP/EB family member 1
Hsa_009575-T1	um	Hsa_009575	chr05	36898200	36918136	ACSS2_1	Propionate--CoA ligase
Hsa_009576-T1	um	Hsa_009576	chr05	36922068	36928976	TAZ	Tafazzin family protein
Hsa_009577-T1	um	Hsa_009577	chr05	36933545	36949085	ATP6AP1_2	ATPase H+ transporting accessory protein 1
Hsa_009578-T1	um	Hsa_009578	chr05	36950123	36955257	NDNL2	Uncharacterized protein
Hsa_009579-T1	do;fr	Hsa_009579	chr05	36955963	36961549	PFKFB1_2	6-phosphofructo-2-kinase/fructose-2,6-biphosphatase 1
Hsa_009580-T1	um	Hsa_009580	chr05	36961622	36973736	PFKFB1_3	6-phosphofructo-2-kinase/fructose-2, 6-bisphosphatase-like isoform X1
Hsa_009581-T1	um	Hsa_009581	chr05	36983300	37029892	L1CAM_4	neural cell adhesion molecule L1.1-like isoform X1
Hsa_009582-T1	um	Hsa_009582	chr05	37050206	37053579	Null	hypothetical protein
Hsa_009583-T1	um	Hsa_009583	chr05	37072145	37078767	OJAV_G00070790	LIM zinc-binding domain-containing protein
Hsa_009584-T1	um	Hsa_009584	chr05	37089504	37096534	PFLUV_G00053050	IG domain-containing protein
Hsa_009585-T1	um	Hsa_009585	chr05	37111493	37148610	CABLES2_3	CDK5 and ABL1 enzyme substrate 2-like
Hsa_009586-T1	um	Hsa_009586	chr05	37157548	37177700	CCM2L	CCM2 like scaffold protein
Hsa_009587-T1	ex	Hsa_009587	chr05	37198225	37280027	XKR7_2	XK-related protein
Hsa_009588-T1	um	Hsa_009588	chr05	37314202	37328662	Null	hypothetical protein

Table S13 Summary of the transcriptome sequencing data used for dosage compensation analysis in *H. sajori*.

Id	Sex	Tissue	Obtained Reads	OBtained Base(bp)	Q20(%)	Q30(%)	GC(%)
SSX01	Male	Muscle	24,274,268	7,258,959,178	98.1	94.74	50.16
SSX01	Male	Liver	24,971,568	7,471,647,322	98.33	95.15	49.54
SSX02	Male	Muscle	19,904,762	5,951,395,686	98.22	95	50.12
SSX02	Male	Liver	21,938,826	6,558,895,534	98.28	95.04	48.96
SSC01	Female	Muscle	22,051,203	6,594,959,692	97.97	94.42	50.62
SSC01	Female	Liver	24,080,835	7,192,245,526	98.43	95.39	48.53
SSC02	Female	Muscle	20,581,940	6,154,736,394	98.4	95.38	50.91
SSC02	Female	Liver	22,345,704	6,684,369,160	98.25	95.02	49.48

Table S14 *P* values of Hartigan's dip test for unimodality corresponding to Fig. 4E-L.

		Computing methods	
Tissue	W/Z expression ratio in females	Linear interpolation	Monte Carlo simulation with 10,000 replicates
muscle	0	0.0001047	< 2.2e-16
	0-0.256	0.6776	0.6737
	0.256-0.667	0.9412	0.9438
	>0.667	0.3307	0.3276
liver	0	0.04233	0.0412
	0-0.256	0.5651	0.5565
	0.256-0.667	0.2495	0.2449
	>0.667	0.195	0.1999

Table S15 Summary information for genes inferred to show possible dosage compensation in muscle tissue. Abbreviations in the Effect column are the same as Table S12.

Gene ID	Gene name	Product	Effect	Zf/ZZmale	Wf/Zf	Male expression	Female expression	Stratum
Hsa_008719	TGM2_3	protein-glutamine gamma-glutamyltransferase 2-like isoform X1	fr	0.87	0.09	27	25.5	Stratum2
Hsa_008821	AHCYL1_1	Adenosylhomocysteinase like 1	NA	1.00	0.00	1	1	Stratum2
Hsa_38016	NTF7	Neurotrophin-7	NA	1.00	0.00	1	1	Stratum2
Hsa_008975	ARHGEF3L	rho guanine nucleotide exchange factor (GEF) 3, like	NA	1.25	0.00	2	2.5	Stratum2
Hsa_008986	WNT5A	Protein Wnt	NA	0.75	0.00	2	2	Stratum2
Hsa_009192	Uncharacterized	IG domain-containing protein	NA	0.75	0.00	4	3	Stratum2
Hsa_009194	FBXO6	F-box only protein 6-like	fr	0.82	0.07	8.5	8	Stratum2
Hsa_009315	GLTPD1	Ceramide-1-phosphate transfer protein, variant 2	sg;sl	0.91	0.20	5.5	5.5	Stratum1
Hsa_009363	HCK	Tyrosine-protein kinase	NA	1.00	0.00	1.5	1.5	Stratum1
Hsa_009364	Null	hypothetical protein	NA	1.08	0.14	6.5	7.5	Stratum1
Hsa_009366	PLAGL2	PLAG1 like zinc finger 2	NA	1.00	0.00	1	1	Stratum1
Hsa_009455	PTPN18	Tyrosine-protein phosphatase non-receptor type 18, variant 2	um	0.71	0.20	3.5	2.5	Stratum1
Hsa_009456	PLXNA3_2	Plexin A3	NA	1.08	0.00	6	6.5	Stratum1
Hsa_009463	PTPN7	Protein-tyrosine-phosphatase	fr	1.00	0.00	2	2	Stratum1
Hsa_009473	LOC114439067	Deoxyribonuclease	NA	0.80	0.00	2.5	2	Stratum1

Hsa_009539	Null	hypothetical protein	sg;fr	0.71	0.23	68.5	59.5	Stratum1
Hsa_009561	FAM58A	Cyclin-Q	NA	0.86	0.17	10.5	11	Stratum1

Table S16 Summary information for genes inferred to show possible dosage compensation in liver tissue. Abbreviations in the Effect column are the same as Table S12.

Gene ID	Gene name	Product	Effect	Zf/ZZmale	Wf/Zf	Male expression	Female expression	Stratum
Hsa_008817	SYPL2	Synaptophysin-like protein 1	NA	1.23	0.22	22	32.5	Stratum2
Hsa_008842	CUNH1ORF194	Testis expressed 36	NA	1.00	0.00	5	5	Stratum2
Hsa_008850	DEF6	DEF6 guanine nucleotide exchange factor	NA	1.00	0.00	1	1	Stratum2
Hsa_008853	LARP4_1	la-related protein 4 isoform X1	NA	1.15	0.24	59	84	Stratum2
Hsa_008999	SLC6A8_1	Transporter, variant 2	NA	0.83	0.20	3	2.5	Stratum2
Hsa_009059	KCTD6	Potassium channel tetramerization domain containing 6	NA	1.22	0.00	9	11	Stratum2
Hsa_009060	OGNB	Mimecan	NA	1.67	0.00	1.5	3	Stratum2
Hsa_009076	OGG1	DNA-(apurinic or apyrimidinic site) lyase	fr	1.23	0.13	6.5	9.5	Stratum2
Hsa_009151	GLS2_1	Glutaminase	do	1.66	0.24	29.5	61	Stratum2
Hsa_009152	COL2A1_1	Collagen, type II, alpha 1a	NA	1.67	0.00	1.5	2.5	Stratum2
Hsa_009207	WAS	WASP actin nucleation promoting factor	NA	1.20	0.17	2.5	3.5	Stratum2
Hsa_009268	SPATS2	Spermatogenesis associated serine rich 2	do	1.14	0.12	11	14	Stratum2
Hsa_009445	ELK1	ETS transcription factor ELK1	NA	1.33	0.00	1.5	2	Stratum1

Hsa_009496	MTHFR	Methylenetetrahydrofolate reductase, variant 2	NA	1.46	0.02	28.5	43	Stratum1
Hsa_009504	PFLUV_G000634 90	Guanylate cyclase domain-containing protein	ac	2.00	0.00	1	2.5	Stratum1

References

Cech JN, Peichel CL. 2015. Identification of the centromeric repeat in the threespine stickleback fish (*Gasterosteus aculeatus*). Chromosome Research. 23:767-779.

Melters DP, Bradnam KR, Young HA, Telis N, May MR, Ruby JG, Sebra R, Peluso P, Eid J, Rank D. 2013. Comparative analysis of tandem repeats from hundreds of species reveals unique insights into centromere evolution. Genome Biology. 14:1-20.

Ranallo-Benavidez TR, Jaron KS, Schatz MC. 2020. GenomeScope 2.0 and Smudgeplot for reference-free profiling of polyploid genomes. Nature Communications. 11:1432.

Viñas A, Abuín M, Pardo BG, Martí P, Sánchez L. 2004. Characterization of a new Hpa I centromeric satellite DNA in *Salmo salar*. Genetica. 121:81-87.

Waterhouse A, Bertoni M, Bienert S, Studer G, Tauriello G, Gumienny R, Heer FT, de Beer TAP, Rempfer C, Bordoli L. 2018. SWISS-MODEL: homology modelling of protein structures and complexes. Nucleic Acids Research. 46:W296-W303.

Wintersinger JA, Wasmuth JD. 2015. Kablammo: an interactive, web-based BLAST results visualizer. Bioinformatics. 31:1305-1306.
